# Selenoxanthylyl radicals – characterization and conPET catalysis

**DOI:** 10.1039/d6cc06178h

**Published:** 2026-10-27

**Authors:** Marc E. Lennon, Till Schreyer, Suwanan Uipanit, Javier Mateos

**Affiliations:** a Institute of Organic Chemistry, University of Vienna Vienna 1090 Austria javier.mateos@univie.ac.at; b Unit for Advanced Magnetic Resonance (AMR), Department of Chemistry and Center of Excellence for Innovation in Chemistry, Faculty of Science, Kasetsart University 50 Ladyao Road, Chatuchak District Bangkok 10900 Thailand

## Abstract

Selenoxanthylium photocatalysts are designed based on their ground-state properties to enable low-energy photon photocatalysis, although reductive quenching of their excited state generates persistent selenoxanthylyl radicals. Herein, we report the characterization of selenoxanthylyl radicals by EPR, cyclic voltammetry, UV-Vis spectroscopy, and TD-DFT calculations, together with their application in consecutive photoinduced electron-transfer (conPET) catalysis.

## Abstract 2

Selenoxanthylyl radicals are characterized by spectroscopy and electrochemistry. Their photoexcitation enables consecutive photoinduced electron-transfer (conPET) catalysis for the reductive detosylation of azaheterocycles.

## Graphical abstract

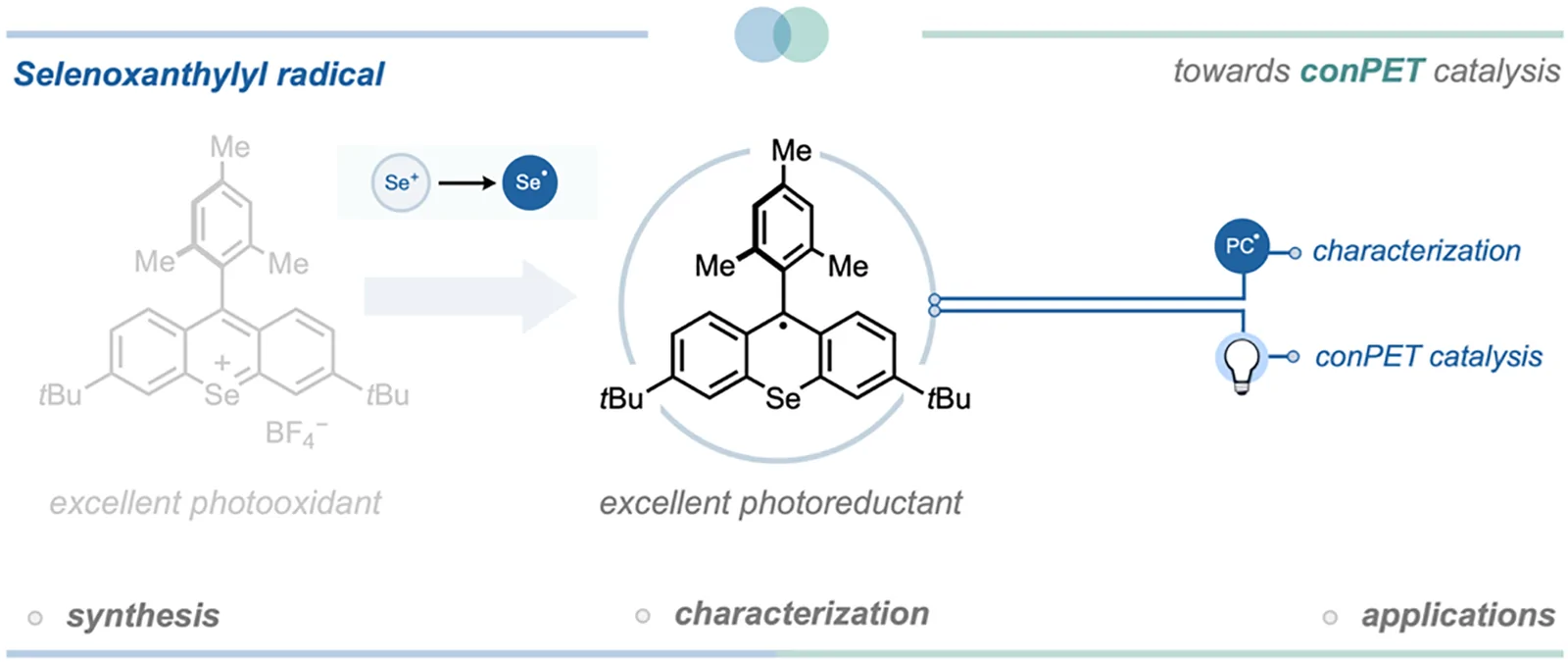

Reductive quenching photocatalysis exploits the excited-state reduction potential 
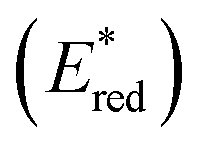
 of photocatalysts to promote substrate oxidation through single electron transfer, generating reduced catalyst intermediates (Fig. 1A).^1^ Cationic organic photocatalysts exhibiting high excited-state reduction potentials 
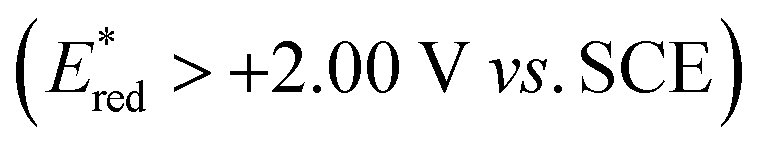
 have found widespread application in oxidative photocatalysis.^2–4^ Although catalyst development has largely been guided by the properties of the cationic ground state,^5^ reduction generates persistent neutral radicals.^6–8^ Radical intermediates are not innocent spectators: (i) their stability towards reoxidation governs catalyst turnover,^9^ and (ii) their persistence allows them to participate in further photochemical reactions.^10^ In particular, photoexcitation of neutral radicals enables consecutive photoinduced electron-transfer (conPET) processes.^11^

**Fig. 1:**
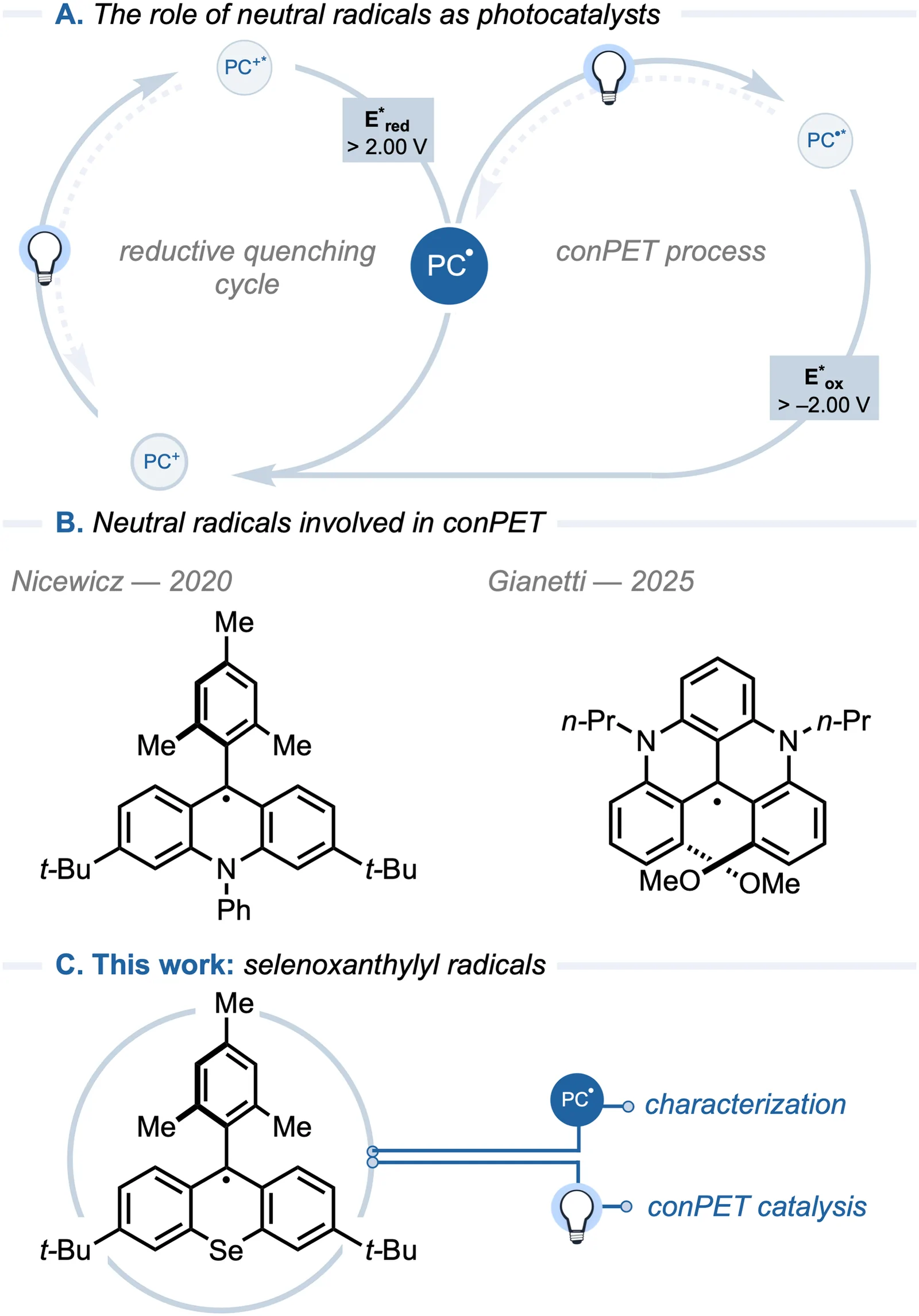
Neutral radicals in reductive photocatalysis. (A) Reductive quenching photoredox cycle and conPET catalysis. (B) Representative neutral organic radicals employed in conPET catalysis. (C) Selenoxanthylyl radicals: characterization and application in conPET catalysis.

Herein, we report the synthesis, characterization, and application of selenoxanthylyl radicals in conPET catalysis (Fig. 1C). Selenoxanthylyl radicals were characterized by cyclic voltammetry, EPR, and UV-Vis spectroscopy; TD-DFT calculations permit rationalization of the spectroscopic features. The resulting insights deepen our understanding of key intermediates in oxidative photocatalysis and expand the synthetic potential of selenoxanthylium photocatalysts by enabling reductive photocatalysis, as demonstrated by the detosylation of heteroaromatics.

One-electron reduction of selenoxanthylium tetrafluoroborate 1(BF_4_) with cobaltocene affords selenoxanthylyl radical 2 (Fig. 2A). Addition of cobaltocene to an acetonitrile solution of 1(BF_4_) results in an immediate colour change from crimson to light yellow. The difference between the cobaltocenium/cobaltocene (*E*° = −1.30 V *vs*. Fc/Fc^+^ in MeCN)^12^ and selenoxanthylium/selenoxanthylyl (*E*° = −0.45 V *vs*. Fc/Fc^+^ in MeCN; Fig. 2B) redox couples makes electron transfer thermodynamically favourable. Radical 2 can be prepared in an Ar-filled glovebox and remains stable for more than 12 h under inert conditions, with no observable decomposition. Exposure to air slowly regenerates selenoxanthylium 1. To confirm the reoxidation of radical 2, we treated it with stoichiometric NOBF_4_ and confirmed the formation of 1(BF_4_) by ^1^H NMR spectroscopy (see Supporting Information, pages S22 and S23). The observed persistence of radical 2 allows characterization *in situ* without prior isolation, as cobaltocenium does not exhibit UV-Vis absorptions or EPR signals that interfere with the characterization of 2 (see SI pp. S21–S26).^13^

**Fig. 2:**
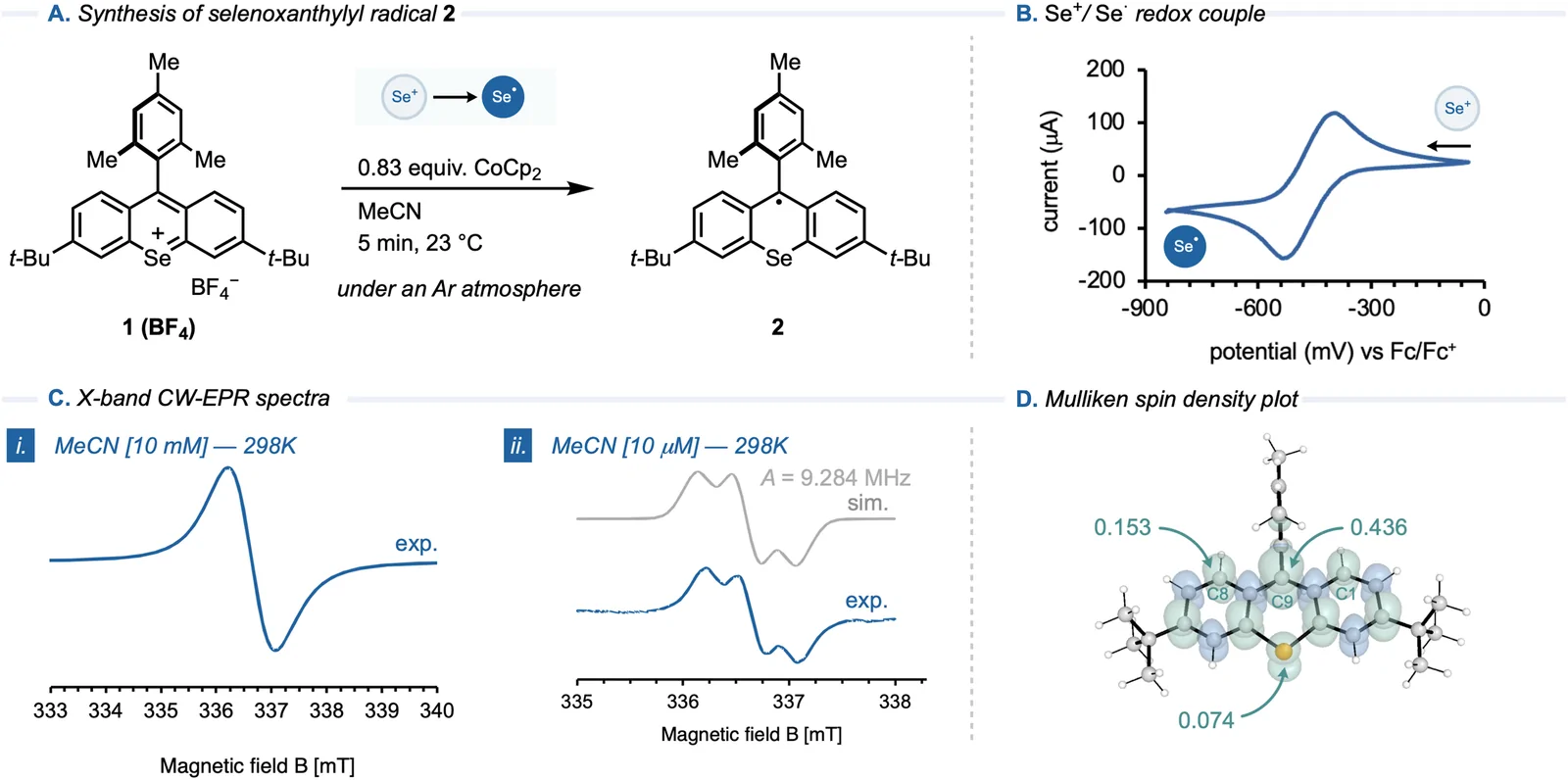
Generation and characterization of selenoxanthylyl radical 2. (A) Reduction of selenoxanthylium salt 1(BF_4_) with cobaltocene affords selenoxanthylyl radical 2. (B) Cyclic voltammogram in MeCN of the Se(iii)/Se(ii) redox couple. (C) Experimental and simulated X-band CW-EPR spectra of radical 2 recorded at 298 K (i) 10 mM and (ii) 10 µM. (D) Mulliken spin-density distribution and spin-density isosurface (isovalue = 0.002) calculated for radical 2 at the ωB97X-D4(SMD, Acetonitrile)/def2-TZVP‖ωB97X-D4(SMD, Acetonitrile)/def2-SVP level of theory.

Continuous-wave X-band (9.45 GHz) EPR spectroscopy confirms the paramagnetic nature of radical 2 (Fig. 2C). The MeCN solution of 2 at 298 K and 10 mM concentration displays an isotropic signal. A diluted (10 µM) MeCN solution exhibits resolved hyperfine coupling to the two equivalent ^1^H nuclei at C1 and C8 (*A* = 9.284 MHz), closely resembling the spectra reported for acridinyl radicals.^8^ DFT calculations rationalize the observed hyperfine pattern through the Mulliken spin-density distribution (Fig. 2D). The unpaired electron is predominantly localized at the benzylic carbon C9 (*ρ* = 0.436), with additional spin density at carbons C1 and C8 (*ρ* = 0.153 each). Selenium also bears a smaller spin density (*ρ* = 0.074). The low spin density at selenium, combined with the low natural abundance of ^77^Se (7.63%),^14^ may account for the absence of resolved hyperfine coupling to ^77^Se under the experimental conditions.

We characterized radical 2 by UV-Vis spectroscopy and rationalized the different absorption bands with TD-DFT calculations.^15^ The combined experimental and computational data account for the observed colour change (Fig. 3A). Selenoxanthylium 1(BF_4_) displays an intense absorption band centred at 560 nm that arises from a transition localized on the heterocyclic core and enables red-light photocatalysis.^16^ Radical 2 exhibits weaker absorption bands between 450 and 600 nm (see SI pp. S24–S25). We attribute the marked change in the absorption profile to disruption of the aromatic π-system of the selenoxanthylium core. Two absorption bands appear in the near-UV/violet region (Fig. 3B): (i) a band centred at 420 nm (*ε*_max_ = 2000 M^−1^ cm^−1^) assigned to the *D*_0_ → *D*_3_ (SOMO → LUMO+1) and *D*_0_ → *D*_4_ (SOMO → LUMO+2) transitions with comparable oscillator strengths, and (ii) an intense band centred at 360 nm (*ε*_max_ = 25 000 M^−1^ cm^−1^) assigned the *D*_0_ → *D*_9_ transition (dominated by a HOMO−3 → SOMO excitation with substantial mixing).

**Fig. 3:**
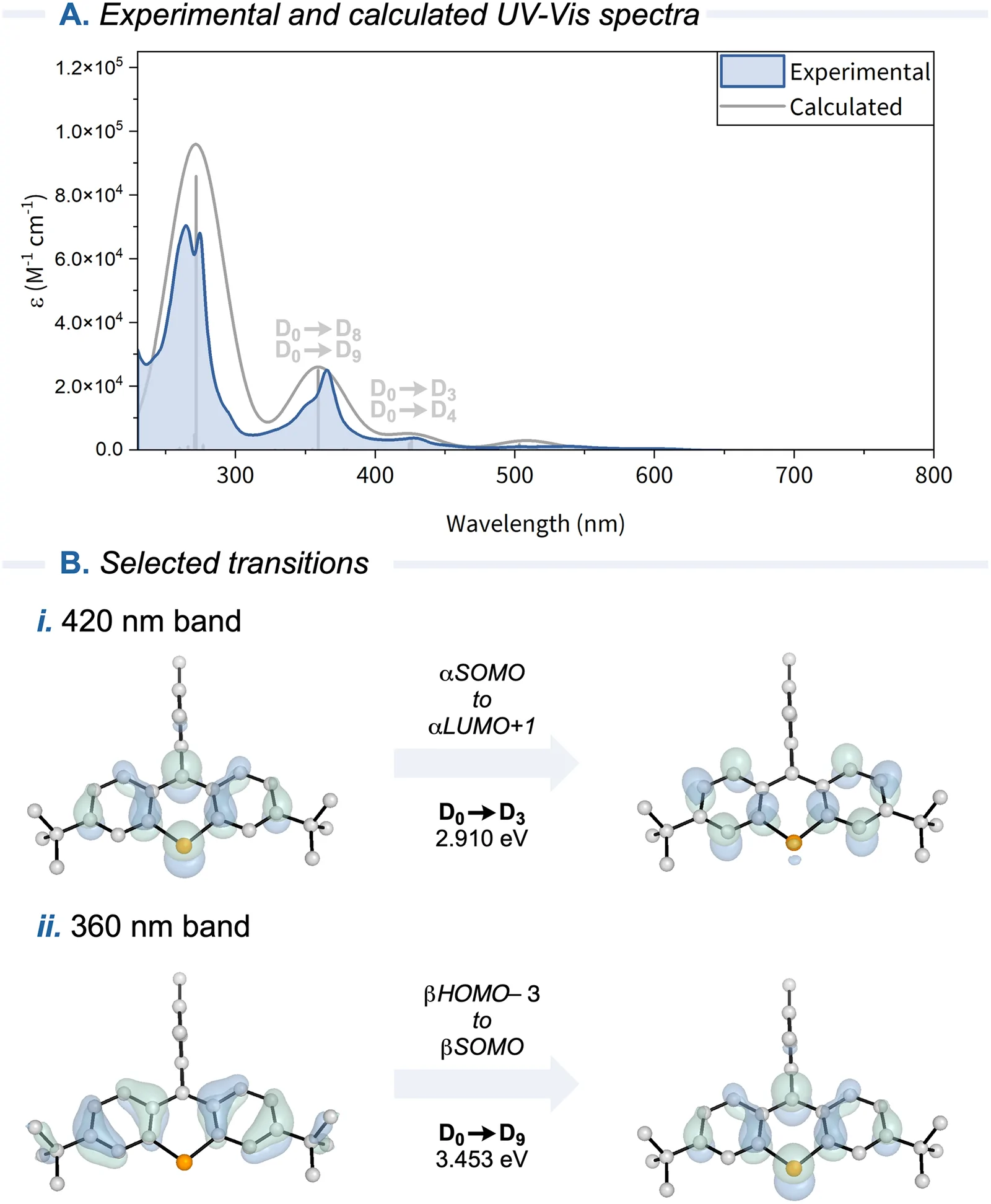
UV-Vis characterization of selenoxanthylyl radical 2. (A) Experimental (blue) and calculated (grey) UV-Vis absorption spectra of radical 2. TD-DFT calculations were performed at the TPSSh-D4/X2C-TZVPall level of theory (RIJCOSX, X2C/J, SMD/Acetonitrile). (B) Selected electronic transitions responsible for the absorption bands at 420 and 360 nm together with the corresponding molecular orbitals. Hydrogen atoms omitted for clarity. Orbital isosurfaces are plotted at an isovalue of 0.04.

Photoexcitation of the *D*_0_ → *D*_9_ transition has proven productive in acridinyl radical photocatalysis, whereas excitation of lower-energy transitions is unproductive.^8,11^ We wondered whether similar behaviour would be observed for selenoxanthylyl radical 2, prompting us to investigate the possibility of catalytic conPET reactions, known for acridinium photocatalysts in the presence of a sacrificial reductant.^17^

We selected the conPET-mediated detosylation of N–Ts azaheterocycles reported by Nicewicz and co-workers as a benchmark transformation (Fig. 4).^17^ Irradiation of selenoxanthylium 1(BF_4_) at 390 nm in the presence of DIPEA as the electron donor affords the detosylation of a range of N–Ts azaheterocycles. Benzimidazole (3), indoles (4 and 5), indazole (6), and carbazole (7) all undergo deprotection to give the corresponding N–H heterocycles. The successful detosylation of these substrates establishes selenoxanthylium salts as competent catalysts for conPET catalysis.

**Fig. 4:**
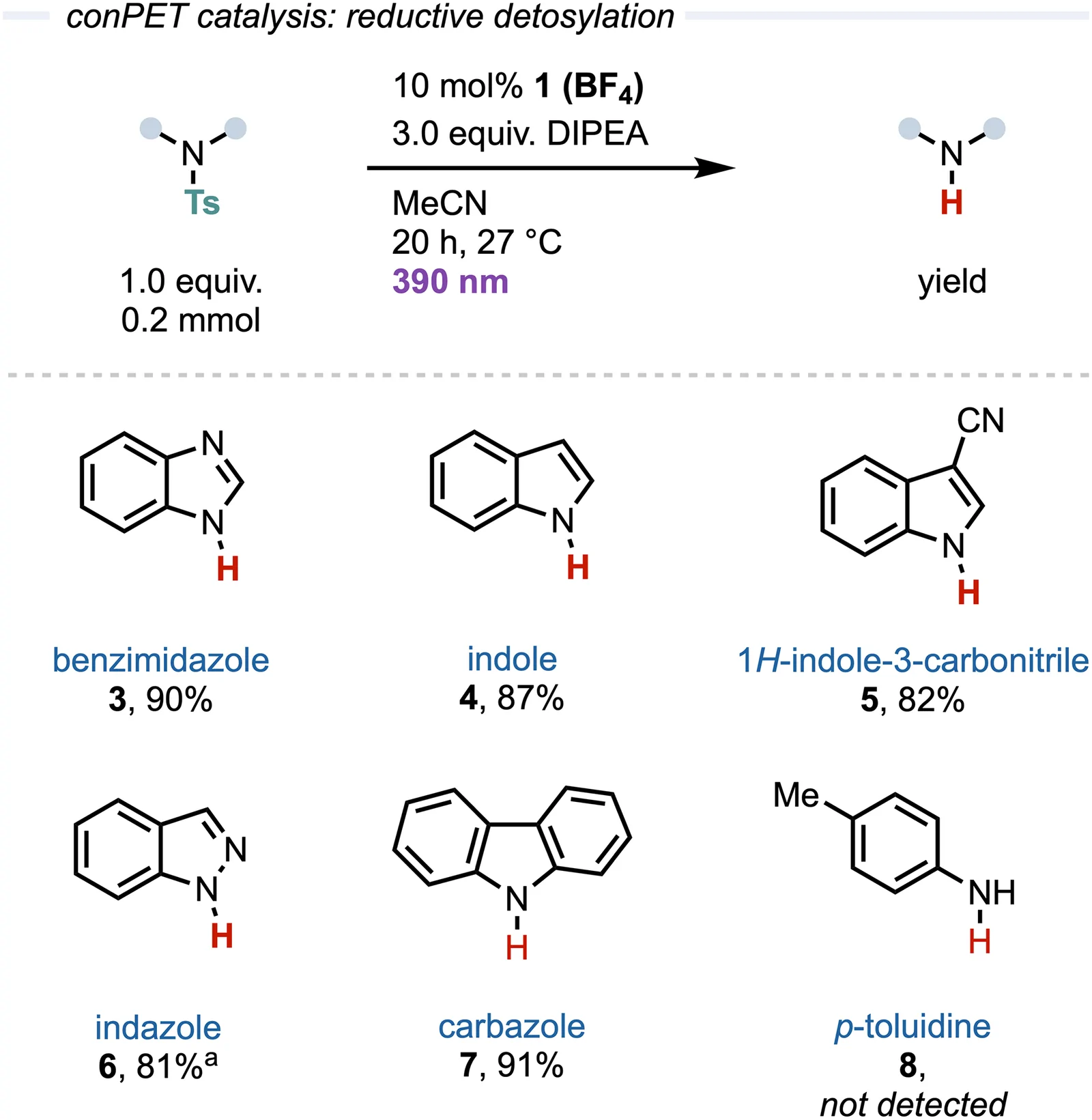
Towards conPET catalysis with selenoxanthylyl radicals. Photocatalytic detosylation of N–Ts azaheterocycles. Reaction conditions: substrate (0.20 mmol, 1.0 equiv.), 1(BF_4_) (10 mol%), DIPEA (3.0 equiv.), MeCN, 390 nm irradiation, 27 °C, 16 h. Isolated yields. For experimental details, see the SI pp. S14–S15. ^a^ Reaction carried out on 0.10 mmol scale.

The limitations of the selenoxanthylyl radical as a photoreductant became apparent in the detosylation of N–Ts anilines (8), which remained unreactive under the reaction conditions (see Supporting Information p. S18).^17^ Irradiation of an equimolar mixture of radical 2 and 4-chlorobenzonitrile at 390 nm for 16 h also afforded no detectable dehalogenation (see Supporting Information p. S18). Both observations are consistent with a less negative excited-state oxidation potential for the selenoxanthylyl radical compared with the acridinyl radical.

In conclusion, we prepared selenoxanthylyl radical 2 from selenoxanthylium photocatalyst 1(BF_4_). *In situ* reduction enabled characterization of the radical by EPR, UV-Vis spectroscopy, and TD-DFT calculations. The spectroscopic and electronic features closely resemble those reported for acridinyl radicals. Accordingly, selenoxanthylium photocatalysts can also participate in conPET processes, as demonstrated by the detosylation of azaheterocycles. Beyond providing insight into key intermediates in selenoxanthylium photocatalysis, this work establishes selenoxanthylium salts as reductive photocatalysts in addition to their established role as red-light organic photocatalysts for oxidative transformations.

## Author contributions

M. E. L. prepared radical 2. M. E. L. and T. S. characterized radical 2. M. E. L. and S. U. performed the photocatalytic reactions. J. M. performed the TD-DFT calculations, secured funding, directed the project, and wrote the manuscript with contributions from all the authors. All authors have given approval to the final version of the manuscript.

## Conflicts of interest

The authors declare no conflict of interest.

## Supplementary Material

CC-OLF-D6CC06178H-s001

## Data Availability

The data supporting this article have been included as part of the supplementary information (SI). Supplementary information: additional experimental details, materials, methods, theoretical calculations, and characterization data (^1^H, ^13^C, ^19^F and ^77^Se NMR, and HRMS) for all new compounds. See DOI: https://doi.org/10.1039/d6cc06178h. The data that support the findings of this study are available from the corresponding author upon reasonable request.
