# Supplementary material for: Selenoxanthylyl radicals – characterization and conPET catalysis
Source: Chem Commun (Camb). 2026 Oct 27. Online ahead of print. doi: 10.1039/d6cc06178h (PMC13725700; doi:10.1039/d6cc06178h)
Supplement: Supplementary file 1 [file CC-OLF-D6CC06178H-s001.pdf]

Supplementary Information (SI) for Chemical Communications.
This journal is © The Royal Society of Chemistry

SUPPORTING INFORMATION

Selenoxanthylyl Radicals – Characterization and conPET Catalysis

Marc E. Lennon,[a] Till Schreyer,[a] Suwanan Uipanit,[ab] Javier Mateos [a] *

[a.] Institute of Organic Chemistry, University of Vienna, Vienna 1090, Austria

[b]Unit for Advanced Magnetic Resonance (AMR), Department of Chemistry and Center of Excellence for Innovation in Chemistry, Faculty of Science, Kasetsart University, 50 Ladyao Road, Chatuchak District, Bangkok, 10900, Thailand

*E-mail: javier.mateos@univie.ac.at

Table of Contents

Table of Contents 1

Materials and Methods 3

Solvents 3

Chromatography 3

Spectroscopy and instruments 3

Computational Data 5

Starting materials 5

Safety statement 5

Author contributions 6

Experimental procedures and data 7

Preparation of *N*-tosyl azacycles and tosylamides 7

ConPET catalysis 14

Light source for photoreactions 14

Photoreactor configuration 14

General procedure for conPET photocatalysis 15

Characterization data of conPET products 15

Limitations 18

Selenoxanthylyl radical synthesis 19

3,6-Di-tert-butyl-9-mesitylxanthylyl radical (**2**) 19

Selenoxanthylyl characterization 21

Cyclic voltammetry 21

EPR spectroscopy 21

Reoxidation study 22

Spectrophotometry 24

Computational Data 27

Comparison between TD-DFT and experimental absorption spectra 27

Selenoxanthylyl radical **2** 27

Coordinates for selenoxanthylyl radical **2** 30

NMR SPECTROSCOPIC DATA 33

REFERENCES 63

Materials and Methods

All air- and moisture-insensitive reactions were carried out under ambient atmosphere in round-bottom flasks, screw-cap vials fitted with rubber septum inserts, or Schlenk flasks. Reaction progress was monitored by thin-layer chromatography (TLC). Concentration under reduced pressure was performed by rotary evaporation at 40 °C and an appropriate pressure. Purified compounds were further dried under high vacuum (0.10–0.005 mbar). Yields refer to isolated and spectroscopically pure compounds, unless otherwise stated.

Solvents

Technical heptane and EtOAc were distilled using a 20 L rotary evaporator. Dry CH_3CN was purchased from Acros Organics, transferred to a flame-dried storage Schlenk flask under an atmosphere of dry argon, and degassed by sparging with dry argon (15 mins) followed by three freeze-pump-thaw (FPT) cycles with dry argon as the refill gas. Deuterated solvents were purchased from Deutero GmbH. Reactions were performed using technical solvents obtained from Acros Organics, TCI, and Sigma-Aldrich, unless otherwise stated.

Chromatography

Thin layer chromatography (TLC) was performed on aluminum plates coated with silica gel 60 F_{254} with 0.20 mm thickness purchased from Macherey-Nagel®. Chromatograms were visualized by fluorescence quenching with UV light at 254 nm or by staining using potassium permanganate or phosphomolybdic acid stains followed by heating. Flash column chromatography was performed using silica gel 60 (230-400 mesh, Macherey-Nagel®).

Spectroscopy and instruments

NMR spectroscopy

^{1}H NMR, ^{13}C NMR, ^{19}F NMR, and ^{77}Se spectra were recorded using a Bruker AV-400, AV-500, AV-600 or AV-700 spectrometer at 300 K. Chemical shifts (δ) are given in parts per million (ppm), referenced to the solvent peak: for $CDCl_3$, defined at δ = 7.26 ppm (^{1}H NMR) and δ = 77.16 ppm (^{13}C NMR); for DMSO-d_6, defined at δ = 2.50 ppm (^{1}H NMR) and δ = 39.52 ppm (^{13}C NMR). Coupling constants (J) are reported in Hertz (Hz). ^{1}H NMR splitting patterns are designated as singlet (s), doublet (d), triplet (t), quartet (q), quintet (quint.) or a combination thereof, as they appeared in the spectrum. Splitting patterns that could not be interpreted or easily visualized are designated as multiplet (m) or broad (br). NMR shifts of heteronuclei were referenced using their respective Ξ value using the *xiref* au program in Bruker Topspin following IUPAC recommendations.[1]

Mass spectrometry

Mass spectra were obtained using a Bruker maXis UHR-TOF spectrometer (70 eV), using electrospray ionization (ESI) or atmospheric-pressure chemical ionization (APCI) or an Agilent 7200B GC/Q-TOF spectrometer (70 eV), using electron ionization (EI). The sum formulas of the detected ions were determined using Bruker Compass DataAnalysis 5.3 based on the mass accuracy ($\Delta m/z \leq 5$ ppm) and isotopic pattern matching (SmartFormula algorithm).

Cyclic voltammetry

Cyclic voltammetry was measured using an Ossila potentiostat, an Ag/$AgNO_3$ (0.10 M in CH_3CN) reference electrode, a glassy carbon disc working electrode, and a platinum disk counter electrode (purchased from “ALS Electrochemistry & Spectroelectrochemistry”). All measurements were carried out in CH_3CN with 0.10 M NBu_4PF_6 as supporting electrolyte if not stated otherwise. The electrolyte was purchased from BLDPharm®, recrystallized twice from ethanol, dried *in vacuo* for 24h ($5\cdot10^{-3}$ bar), and stored in a desiccator. Voltammograms were referenced to the Fc/Fc^+ redox couple as internal standard. If Fc/Fc^+ was found to react with the target analyte, the voltammogram was first measured against the Fc/Fc^+ calibrated reference electrode (Ag/$AgNO_3$) and then externally referenced to the Fc/Fc^+ redox couple (+0.053 V in CH_3CN). Conversion of the Fc/Fc^+ referenced potentials to SCE was done by adding the potentials reported by Pavlishchuk and Addison (Fc/Fc^+: 0.380 V vs SCE).[2]

UV-Vis absorption and emission spectroscopy

UV-Vis absorption spectroscopy was recorded using an Agilent Cary 5000 UV vis–NIR Spectrophotometer running in double beam mode with a matched pair of quartz absorbance cuvettes (1 × 1 cm). All absorption measurements were performed at room temperature unless specified otherwise. The molar attenuation coefficient (ε) was determined by dissolving a known amount of compound in an argon-filled glovebox and diluting the resulting stock solution to achieve 5 solutions with concentrations ranging from $5.2\cdot10^{-6}$ M to $8.3\cdot10^{-5}$ M. Cuvettes were closed with a PTFE stopper and subsequently sealed using electric tape before removal from the glovebox. The plot of absorbance versus concentration was fitted with a linear function and the molar attenuation coefficient was obtained from the slope. Emission spectra were recorded on an Agilent Technologies Cary Eclipse G9800A spectrofluorometer equipped with a Xenon flash lamp (80 Hz). The sample concentration was adjusted such that the maximum absorbance did not exceed 0.2 to avoid an inner filter effect.

EPR spectroscopy

Continuous-wave (CW) X-band (9.45 GHz) EPR spectra were recorded on a Bruker Magnettech ESR5000 spectrometer equipped with an X-band high-sensitivity TE102 rectangular resonator. Measurements were performed at room temperature (*ca.* 298 K). Samples (20 µL) were prepared in an argon-filled glovebox by drawing analyte solution into a microcapillary tube (Hirschmann Ringcaps) and then sealing the tube termini with plugs of *Critoseal* putty (Noxygen). Unless stated otherwise, CW X-band EPR spectra were recorded

using a field modulation frequency of 100 kHz and a microwave power of 10 mW. The modulation amplitude was 0.2 mT. Simulations were carried out using Easyspin[3] and MATLAB was used for further analysis.

Computational Data

All calculations employed the ORCA 6.0.1 package.[4] Geometry optimizations were carried out at the ωB97X-D4/def2-SVP level of theory,[5] including Grimme's D4 dispersion correction.[6] Tight convergence criteria (TightSCF, TightOpt) and a fine integration grid (DefGrid3) were applied. Vibrational frequency calculations confirmed all stationary points as minima and provided zero-point energies and thermal corrections. Single-point energies were computed at the ωB97X-D4/def2-TZVP level with solvation effects included via the SMD model (acetonitrile). The ORCA 6.0.1 software was applied for the spin density distribution calculations (ωB97X-D3/def2-TZVP,CPCM(Acetonitrile)//ωB97X-D/def2-SVP,SMD(Acetonitrile).[4]

Time-dependent DFT (TD-DFT) calculations were subsequently carried out as single-point calculations on the optimized geometries using five exchange–correlation functionals (ωB97X-D4, CAM-B3LYP-D4, PBE0-D4, B3LYP-D4, and TPSSh-D4) in combination with the def2-TZVP basis set. The performance of each functional was benchmarked against the experimental UV–Vis absorption spectra, and TPSSh-D4 was selected for the analysis of the excited-state properties, canonical molecular orbitals involved in the dominant electronic transitions, and spin-orbit coupling matrix elements (SOCMEs). Scalar relativistic effects were included through the exact two-component (X2C) Hamiltonian.[7,8] Calculated absorption intensities include electric dipole, magnetic dipole, and electric quadrupole contributions.

Molecular graphics were generated using the *PyMOL Molecular Graphics System, Version 3.1.8*.

Starting materials

All substrates were used as received from commercial suppliers (Sigma-Aldrich, TCI, Fluorochem, or BLDPharm), unless otherwise stated.

Safety statement

The procedures reported in this work are intended for use only by individuals with proper training in experimental chemistry. All hazardous materials (solid, liquid, or gaseous) should be handled using standard work procedures such as those described in Chapter 4 of "Prudent Practices in the Laboratory".[9] All chemical waste should be disposed of in accordance with local regulations. For general guidelines for the management of chemical waste, see Chapter 8 of "Prudent Practices in the Laboratory".[9]

During this study, no explosions or violent decompositions occurred. A summary of the possible risks and hazards is described below:

Caution: When performing reactions in pressurized systems (such as closed vials, pressure tubes, and autoclaves), a blast shield must be used to minimize personal damage in case of an accident.

Caution: High-intensity LED sources can pose significant hazards, particularly due to emission capable of causing eye and skin damage. Direct or reflected exposure should be strictly avoided. Experiments should be conducted using appropriate shielding or enclosed setups, and wavelength-specific protective eyewear is recommended. In addition, thermal management is required, as high-power LEDs may generate substantial heat.

Author contributions

Conceptualization: Javier Mateos conceptualized the project.

Investigation: All the authors contributed to the investigation of this work.

Methodology: The synthetic methodology reported in this work was developed by Marc E. Lennon and Suwanan Uipanit.

DFT calculations: The calculations reported in this work were performed by Javier Mateos.

Radical synthesis: The selenoxanthylyl radical was synthesized by Marc E. Lennon.

Characterization: UV-Vis absorption, photoluminescence and cyclic voltammetry studies were performed by Till Schreyer. EPR studies were performed by Marc E. Lennon.

Writing: The original draft was written by Javier Mateos. All authors contributed to the final version of this work.

Direction: Javier Mateos directed the project and secured the funding.

All authors have given approval to the final version of the manuscript.

Experimental procedures and data

Preparation of *N*-tosyl azacycles and tosylamides

*1-Tosyl-1*H-*benzo[*d*]imidazole (**S1**)*

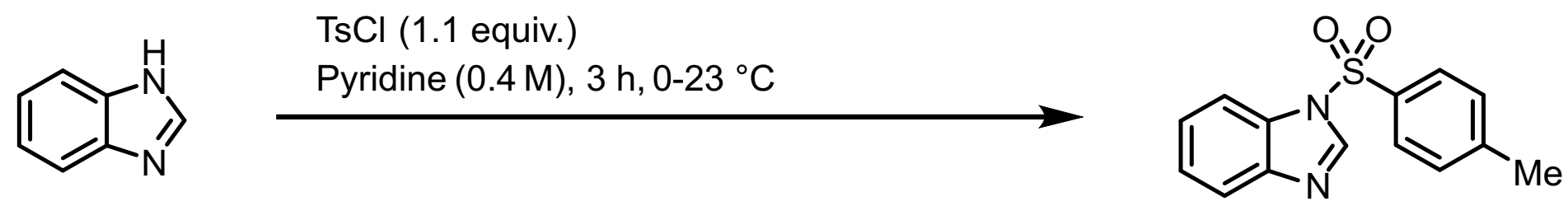

S1

Adapted from the procedure reported by Nicewicz and coworkers.[10] In a 50 mL single-necked round-bottom flask equipped with a magnetic stir bar, benzimidazole (591 mg, 5.0 mmol, 1.0 equiv.) was dissolved in pyridine (12.5 mL, 0.4 M) and cooled to 0 °C. Tosyl chloride (1.08 g, 5.5 mmol, 1.1 equiv.) was added with stirring and the reaction was allowed to warm to r.t. over 3 h. The mixture was concentrated by rotary evaporation under reduced pressure and then poured into water. The aqueous mixture was extracted with CH_2Cl_2 (3x 20 mL), and the combined organic phases were dried over Na_2SO_4, filtered through a cotton plug, and concentrated under reduced pressure. The residue was purified by flash column chromatography on silica gel (heptane/EtOAc) to afford the title compound as a colourless solid in 91% yield (1.24 g, 4.55 mmol).

R*f* = 0.26 (4:1 heptane/EtOAc).

NMR Spectroscopy:

^{1}H NMR (400 MHz, $CDCl_3$, 23 ºC, δ): 8.38 (s, 1H), 7.90–7.83 (m, 3H), 7.79–7.73 (m, 1H), 7.42–7.32 (m, 2H), 7.32–7.27 (m, 2H), 2.37 (s, 3H).

^{13}C NMR (101 MHz, $CDCl_3$, 23 ºC, δ): 146.3, 144.1, 141.4, 134.7, 130.9, 130.5, 127.4, 125.7, 124.9, 121.2, 112.6, 21.8.

HRMS-ESI(m/z) calc'd for $C_{14}H_{13}N_2O_2S$ $[M+H]^+$, 273.0692; found, 273.0687; deviation: –1.8 ppm.

*1-Tosyl-1*H-*indole (**S2**)*

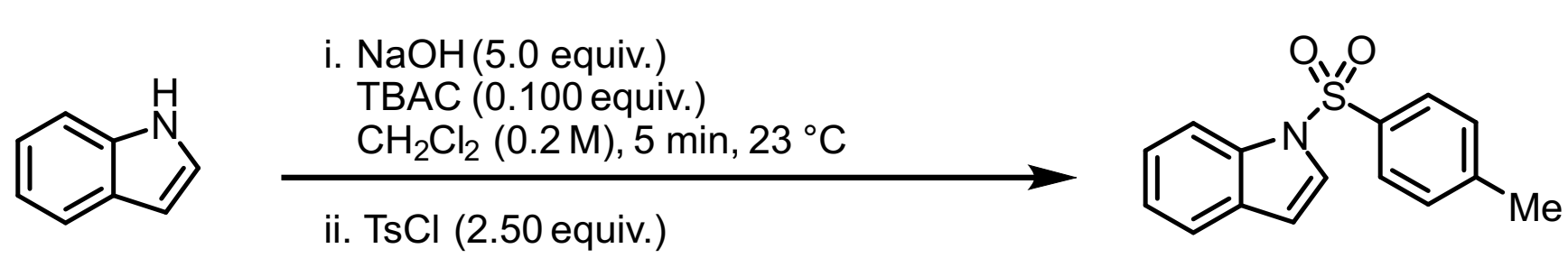

S2

Procedure

Adapted with modifications from the procedure reported by Zhu and coworkers.[11] In a 100 mL single-necked round-bottom flask equipped with a magnetic stir bar, indole (1.20 g, 10.0 mmol, 1.00 equiv.) was dissolved

in CH_2Cl_2 (50 mL, 0.2 M). Tetrabutylammonium chloride (287 mg, 1.00 mmol, 0.100 equiv.) was added, followed by freshly pulverized solid NaOH (2.0 g, 50 mmol, 5.0 equiv.). The reaction was stirred for 5 min, then tosyl chloride (4.81 g, 25.0 mmol, 2.50 equiv.) was added and stirring was continued at room temperature. When complete consumption of the indole was observed by TLC, the reaction was quenched with 1 M HCl. The aqueous mixture was extracted with CH_2Cl_2 (3x 20 mL), and the combined organic phases were washed sequentially with sat. aq. $NaHCO_3$ (20 mL) and water (20 mL), then dried over $MgSO_4$, filtered over cotton, and concentrated by rotary evaporation under reduced pressure. The crude residue was purified by flash column chromatography (heptane/EtOAc) to afford the title compound as a colourless solid in 39% yield (1.06 g, 3.92 mmol).

R*f* = 0.33 (10:1 heptane/EtOAc).

NMR Spectroscopy:

^{1}H NMR (400 MHz, $CDCl_3$, 23 ºC, δ): δ 8.00 (dq, *J* = 8.4, 0.9 Hz, 1H), 7.80–7.73 (m, 2H), 7.57 (d, *J* = 3.7 Hz, 1H), 7.55–7.49 (m, 1H), 7.31 (ddd, *J* = 8.4, 7.2, 1.3 Hz, 1H), 7.26–7.16 (m, 3H), 6.65 (dd, *J* = 3.6, 0.9 Hz, 1H), 2.33 (s, 3H).

^{13}C NMR (101 MHz, $CDCl_3$, 23 ºC, δ): 145.0, 135.5, 135.0, 130.9, 130.0, 126.9, 126.6, 126.5, 124.7, 123.4, 121.5, 113.7, 109.2, 21.7.

HRMS-ESI(m/z) calc'd for $C_{15}H_{13}NO_2SNa$ $[M+Na]^+$, 294.0559; found, 294.0561; deviation: +0.7 ppm.

*1-Tosyl-1*H*-indole-3-carbonitrile (**S3**)*

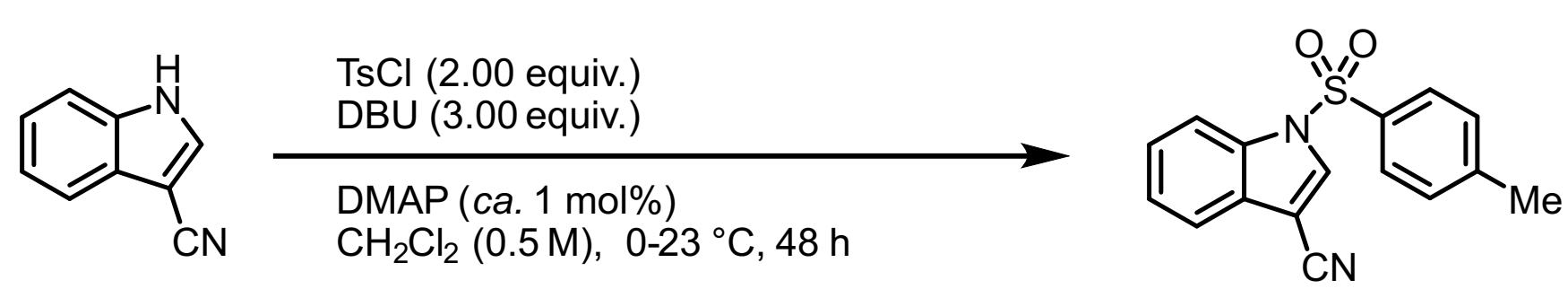

S3

In a 50 mL single-necked round-bottom flask equipped with a magnetic stir bar, 1*H*-indole-3-carbonitrile (711 mg, 5.00 mmol, 1.00 equiv.) was dissolved in CH_2Cl_2 (10 mL, 0.5 M). DBU (2.24 mL, 15.0 mmol, 3.00 equiv.) was added and the reaction was cooled to 0 °C. Tosyl chloride (1.97 g, 10.0 mmol, 2.00 equiv.) was then added, followed by a microspatula tip of DMAP. The reaction was allowed to warm slowly to r.t., stirred for 48 h, then quenched with 1 M HCl. The aqueous mixture was extracted with CH_2Cl_2 (3x 20 mL), and the combined organic phases were washed sequentially with sat. aq. $NaHCO_3$ (20 mL) and water (20 mL), then dried over $MgSO_4$, filtered over cotton, and concentrated by rotary evaporation under reduced pressure. The crude residue was purified by recrystallisation from hot CH_2Cl_2 to afford the title compound as a straw-coloured solid in 30% yield (441 mg, 1.49 mmol).

R*f* = 0.41 (4:1 heptane/EtOAc).

NMR Spectroscopy:

^{1}**H NMR** (400 MHz, $CDCl_3$, 23 ºC, δ): 8.10 (s, 1H), 8.00 (dt, *J* = 8.3, 1.0 Hz, 1H), 7.85–7.80 (m, 2H), 7.69 (ddd, *J* = 7.8, 1.3, 0.8 Hz, 1H), 7.44 (ddd, *J* = 8.3, 7.3, 1.3 Hz, 1H), 7.38 (ddd, *J* = 8.3, 7.3, 1.0 Hz, 1H), 7.32–7.28 (m, 2H), 2.38 (s, 3H).

^{13}**C NMR** (101 MHz, $CDCl_3$, 23 ºC, δ): 146.5, 134.3, 133.8, 133.3, 130.5, 128.5, 127.4, 126.7, 124.9, 120.4, 113.9, 113.6, 93.8, 21.8.

HRMS-ESI(m/z) calc'd for $C_{16}H_{11}N_2O_2S$ $[M–H]^–$, 295.0547; found, 295.0550; deviation: +1.0 ppm.

*1-Tosyl-1*H*-indazole (**S4**)*

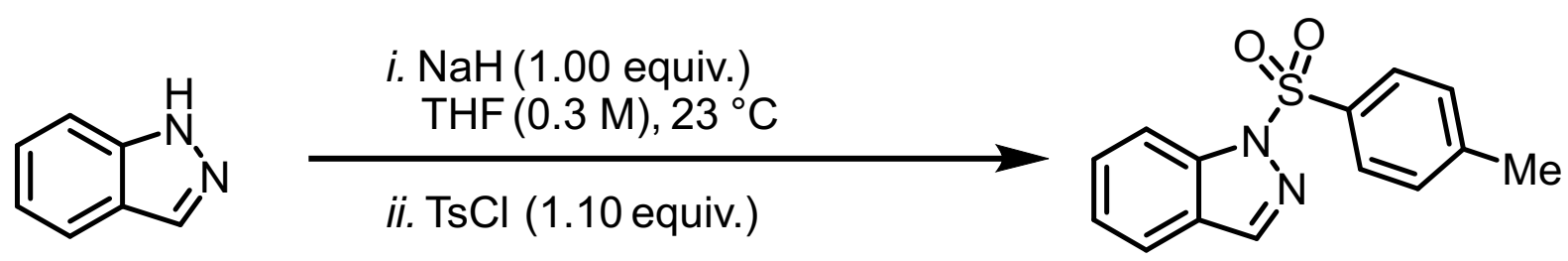

S4

In an oven-dried Schlenk tube equipped with a magnetic stir bar, under an atmosphere of dry argon, indazole (365 mg, 3.00 mmol, 1.00 equiv.) was dissolved in THF (10 mL, 0.3 M). Sodium hydride (60% dispersion in mineral oil, 120 mg, 3.00 mmol, 1.00 equiv.) was added in small portions with vigorous stirring. After effervescence subsided, tosyl chloride (635 mg, 3.30 mmol, 1.10 equiv.) was added in small portions. When complete consumption of the indazole was observed by TLC, the reaction was quenched with 1 M HCl. The aqueous mixture was extracted with CH_2Cl_2 (3x 20 mL), and the combined organic phases were washed sequentially with sat. aq. $NaHCO_3$ (20 mL) and water (20 mL), then dried over $MgSO_4$, filtered over cotton, and concentrated by rotary evaporation under reduced pressure. The crude residue was purified by flash column chromatography (heptane/EtOAc) to afford the title compound in as a colourless solid in 52% yield (429 mg, 1.57 mmol).

$\mathbf{R}_f$ = 0.23 (4:1 heptane/EtOAc).

NMR Spectroscopy:

^{1}**H NMR** (400 MHz, $CDCl_3$, 23 ºC, δ): 8.22 (dq, *J* = 8.5, 0.9 Hz, 1H), 8.18 (d, *J* = 0.9 Hz, 1H), 7.89–7.84 (m, 2H), 7.68 (dt, *J* = 8.0, 1.0 Hz, 1H), 7.56 (ddd, *J* = 8.4, 7.1, 1.0 Hz, 1H), 7.32 (ddd, *J* = 8.0, 7.1, 0.9 Hz, 1H), 7.26–7.20 (m, 2H), 2.35 (s, 3H).

^{13}**C NMR** (101 MHz, $CDCl_3$, 23 ºC, δ): 145.5, 141.4, 140.4, 134.8, 130.0, 129.4, 127.7, 126.0, 124.3, 121.5, 113.3, 21.8.

HRMS-ESI(m/z) calc'd for $C_{14}H_{13}N_2O_2S$ $[M+H]^+$, 273.0692; found, 273.0688; deviation: –1.5 ppm.

*9-Tosyl-9*H*-carbazole (**S5**)*

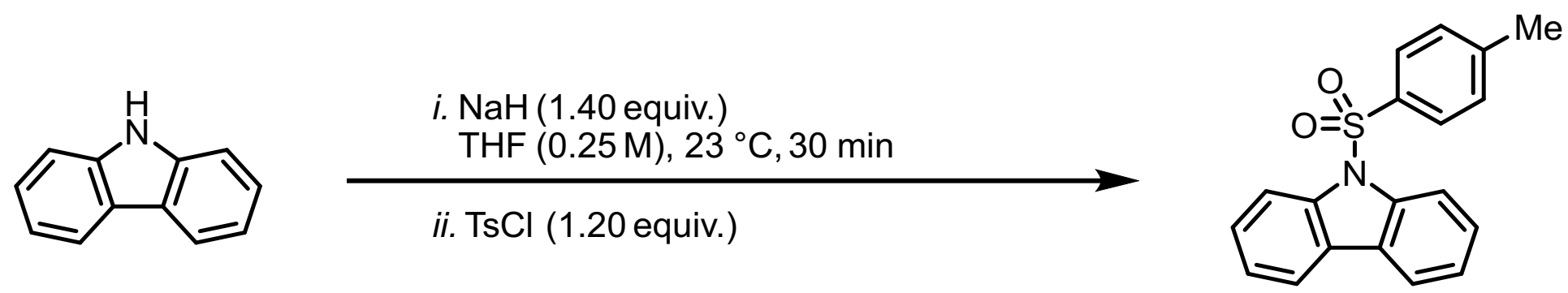

S5

In a flame-dried 50 mL Schlenk flask under an atmosphere of dry argon, carbazole (862 mg, 5.00 mmol, 5.00 equiv.) was dissolved in 20 mL anhydrous THF (0.25 M). Sodium hydride (60% dispersion in mineral oil, 280 mg, 7.00 mmol, 1.40 equiv.) was added in small portions with vigorous stirring. After effervescence ceased, the mixture was stirred for an additional 30 mins. Tosyl chloride (1.16 g, 6.00 mmol, 1.20 equiv.) was then added portionwise. The reaction was monitored by TLC and, upon complete consumption of the starting material, was quenched with water, then acidified with 3 M hydrochloric acid and extracted with CH_2Cl_2 (3x 30 mL). The combined organic extracts were dried over $MgSO_4$, filtered over cotton, and concentrated by rotary evaporation under reduced pressure. The crude residue was purified by flash column chromatography to afford the title compound as a colourless solid in 79% yield (1.26 g, 3.93 mmol).

R*f* = 0.37 (10:1 heptane/EtOAc).

NMR Spectroscopy:

^{1}H NMR (400 MHz, $CDCl_3$, 23 ºC, δ): 8.34 (dt, *J* = 8.4, 0.8 Hz, 2H), 7.90 (ddd, *J* = 7.6, 1.3, 0.8 Hz, 2H), 7.77–7.64 (m, 2H), 7.49 (ddd, *J* = 8.4, 7.3, 1.3 Hz, 2H), 7.36 (td, *J* = 7.6, 1.0 Hz, 2H), 7.17–7.00 (m, 2H), 2.25 (s, 3H).

^{13}C NMR (101 MHz, $CDCl_3$, 23 ºC, δ): 145.0, 138.5, 135.1, 129.8, 127.5, 126.6, 126.5, 124.0, 120.1, 115.3, 21.6.

HRMS-ESI(m/z) calc'd for $C_{19}H_1NO_2SNa$ $[M+Na]^+$, 344.0716; found, 344.0715; deviation: -0.3 ppm.

*4-Methyl-*N*-phenyl-*N*-tosylbenzenesulfonamide (**S6**)*

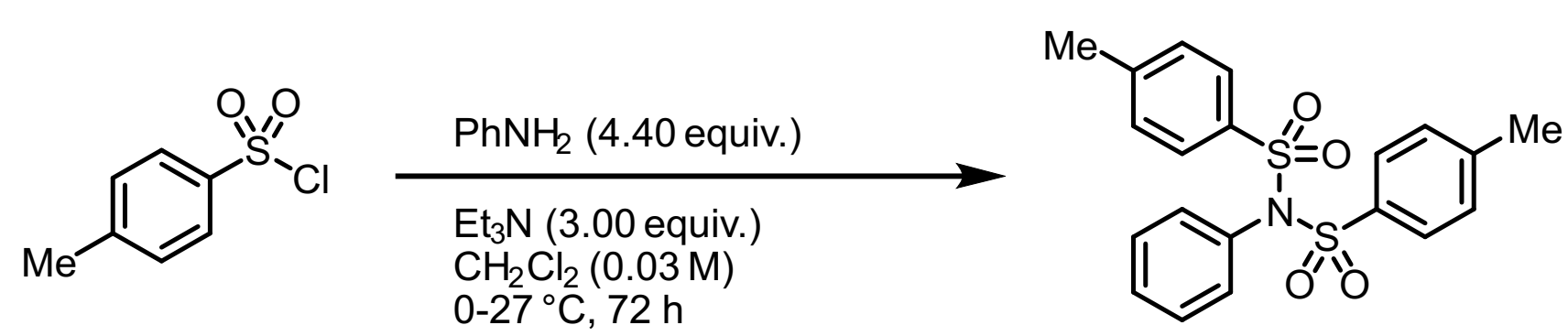

S6

In a 100 mL single-necked round-bottom flask equipped with a magnetic stir bar, aniline (421 mg, 4.40 mmol, 4.40 equiv.) and triethylamine (304 mg, 0.42 mL, 3.00 mmol, 3.00 equiv.) were dissolved in CH_2Cl_2 (20 mL) and cooled to 0 °C. A solution of tosyl chloride (197 mg, 1.00 mmol, 1.00 equiv.) in CH_2Cl_2 (10 mL) was added dropwise to the aniline solution with vigorous stirring (final concentration: 0.03 M w.r.t. TsCl) and

the reaction mixture was allowed to warm slowly to r.t. over 72 h, after which time TLC indicated complete consumption of TsCl. The reaction mixture was partitioned between $CHCl_3$ (20 mL) and 2 M HCl (20 mL) and the aqueous phase extracted once with further $CHCl_3$. The combined organic phases were dried over MgSO4, filtered through a cotton plug, and concentrated by rotary evaporation under reduced pressure. The residue was purified by flash column chromatograph on silica gel (heptane/EtOAc) to afford the title compound as a colourless solid in 30% yield (59.6 mg, 0.148 mmol).

R*f* = 0.41 (4:1 heptane/EtOAc).

NMR Spectroscopy:

^{1}H NMR (400 MHz, $CDCl_3$, 23 ºC, δ): 7.85–7.78 (m, 4H), 7.47–7.40 (m, 1H), 7.38–7.30 (m, 6H), 7.06–7.00 (m, 2H), 2.47 (s, 6H).

^{13}C NMR (101 MHz, $CDCl_3$, 23 ºC, δ): 145.1, 136.8, 134.6, 131.7, 130.3, 129.7, 129.3, 128.7, 21.9.

HRMS-ESI(m/z) calc'd for $C_{20}H_{19}NO_4S_2Na$ $[M+Na]^+$, 424.0648; found, 424.0661; deviation: +3.1 ppm.

*4-Methyl-*N*-phenylbenzenesulfonamide (**S7**)*

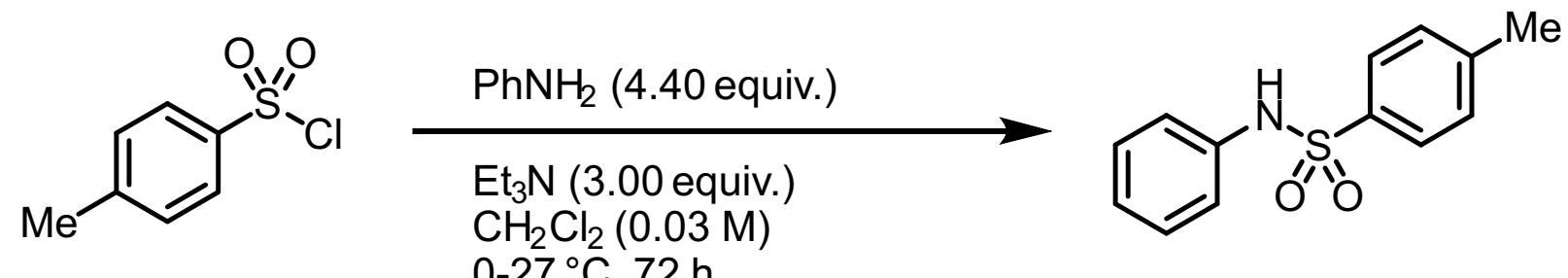

S7

In a 100 mL single-necked round-bottom flask equipped with a magnetic stir bar, aniline (421 mg, 0.41 mL, 4.4 mmol, 4.4 equiv.) and triethylamine (304 mg, 0.42 mL, 3.0 mmol, 3.0 equiv.) were dissolved in CH_2Cl_2 (20 mL) and cooled to 0 °C. A solution of tosyl chloride (197 mg, 1.0 mmol, 1.0 equiv.) in CH_2Cl_2 (10 mL) was added dropwise to the aniline solution with vigorous stirring (final concentration: 0.03 M w.r.t. TsCl) and the reaction was allowed to warm slowly to r.t. over 72 h, after which time TLC indicated complete consumption of TsCl. The reaction mixture was partitioned between $CHCl_3$ (20 mL) and 2 M HCl (20 mL) and the aqueous phase extracted once with further $CHCl_3$. The combined organic phases were dried over $MgSO_4$, filtered through a cotton plug, and concentrated by rotary evaporation under reduced pressure. The residue was purified by flash column chromatography on silica gel (heptane/EtOAc) to afford the title compound as a colourless solid in 38% yield (93.4 mg, 0.379 mmol).

R*f* = 0.18 (4:1 heptane/EtOAc).

NMR Spectroscopy:

^{1}H NMR (400 MHz, $CDCl_3$, 23 ºC, δ): 7.69–7.63 (m, 2H), 7.26–7.19 (m, 4H), 7.13–7.04 (m, 3H), 6.82 (s, 1H), 2.37 (s, 3H).

^{13}C NMR (101 MHz, $CDCl_3$, 23 ºC, δ): ^{13}C NMR (101 MHz, $CDCl_3$) δ 144.0, 136.7, 136.2, 129.8, 129.5,

127.4, 125.5, 121.7, 21.7.

HRMS-ESI(m/z) calc'd for $C_{13}H_{14}NO_2S$ $[M+H]^+$, 248.0740; found, 248.0740; deviation: 0.0 ppm.

*4-Methyl-*N*-(*p*-tolyl)benzenesulfonamide (**S8**)*

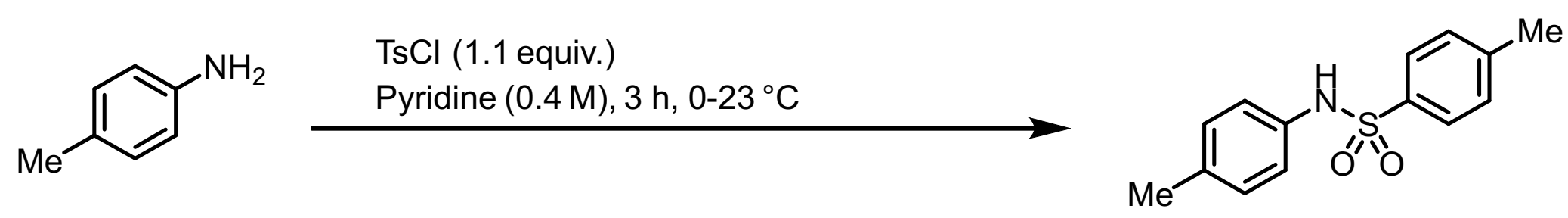

S8

Adapted from the procedure reported by Nicewicz and coworkers.[10] In a 50 mL single-necked round-bottom flask equipped with a magnetic stir bar, *para*-toluidine (536 mg, 5.00 mmol, 1.00 equiv.) was dissolved in pyridine (12.5 mL, 0.4 M) and cooled to 0 °C. Tosyl chloride (1.08 g, 5.50 mmol, 1.10 equiv.) was added with stirring and the reaction was allowed to warm to r.t. over 3 h. The pyridine was then removed by rotary evaporation, and the residue was poured into water. The aqueous mixture was extracted with CH_2Cl_2 (3x 20 mL), and the combined organic phases were dried over Na_2SO_4, filtered through a cotton plug, and concentrated under reduced pressure. The residue was purified by flash column chromatography on silica gel (heptane/EtOAc) to afford the title compound as a colourless solid in 95% yield (1.23 g, 4.72 mmol).

R_f = 0.20 (4:1 heptane/EtOAc).

NMR Spectroscopy:

^{1}H NMR (400 MHz, $CDCl_3$, 23 ºC, δ): 7.69–7.62 (m, 2H), 7.24–7.18 (m, 2H), 7.05–6.93 (m, 5H), 2.37 (s, 3H), 2.26 (s, 3H).

^{13}C NMR (101 MHz, $CDCl_3$, 23 ºC, δ): 143.8, 136.2, 135.4, 133.9, 129.9, 129.7, 127.4, 122.3, 21.6, 20.9.

HRMS-ESI(m/z) calc'd for $C_{14}H_{14}NO_2S$ $[M–H]^–$, 260.0750; found, 260.0747; deviation: –1.1 ppm.

N*-(4-(*tert*-Butyl)phenyl)-4-methylbenzenesulfonamide (**S9**)*

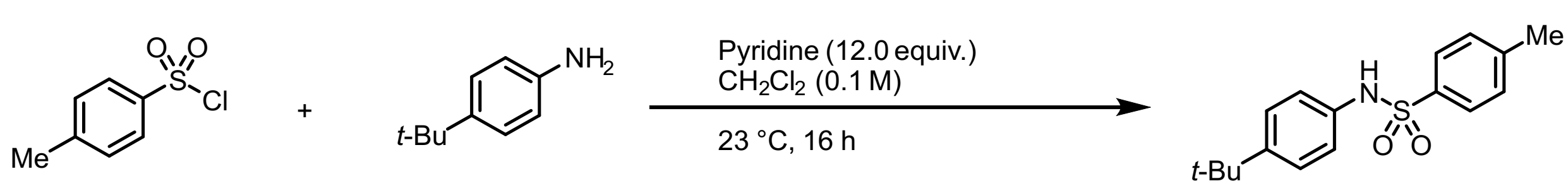

1.00 equiv. 5.00 equiv. **S9**

In a 50 mL single-necked round-bottom flask equipped with a magnetic stir bar, 4-*tert*-butylaniline (751 mg, 0.800 mL, 5.00 mmol, 5.00 equiv.) and pyridine (0.980 mL, 12.0 mmol, 12.0 equiv.) were dissolved in CH_2Cl_2 (10 mL). Tosyl chloride (197 mg, 1.00 mmol, 1.00 equiv.) was added to the aniline solution with vigorous stirring (final concentration: 0.1 M w.r.t. TsCl) and the reaction was allowed to stir overnight. The reaction mixture was partitioned between $CHCl_3$ (20 mL) and 2 M HCl (20 mL) and the aqueous phase extracted

once with further $CHCl_3$. The combined organic phases were washed with further 2 M HCl (2x 20 mL) and sat. aq. $NaHCO_3$ (20 mL) then dried over $MgSO_4$, filtered through a cotton plug, and concentrated by rotary evaporation under reduced pressure. The residue was purified by flash column chromatography on silica gel (heptane/EtOAc) to afford the title compound as a pale pink solid in 99% yield (302 mg, 0.993 mmol).

R*f* = 0.22 (4:1 heptane/EtOAc).

NMR Spectroscopy:

> **^{1}H NMR** (400 MHz, $CDCl_3$, 23 ºC, δ): 7.69–7.63 (m, 2H), 7.26–7.19 (m, 4H), 7.00–6.94 (m, 2H), 6.59 (s, 1H), 2.39 (s, 3H), 1.26 (s, 9H).
>
> **^{13}C NMR** (101 MHz, $CDCl_3$, 23 ºC, δ): 148.7, 143.9, 136.6, 133.8, 129.7, 127.4, 126.3, 121.8, 34.5, 31.4, 21.7.

HRMS-ESI(m/z) calc'd for $C_{17}H_{22}NO_2S$ $[M+H]^+$, 304.1366; found, 304.1358; deviation: –2.6 ppm.

*2-Tosyl-1,2,3,4-tetrahydroisoquinoline (**S10**)*

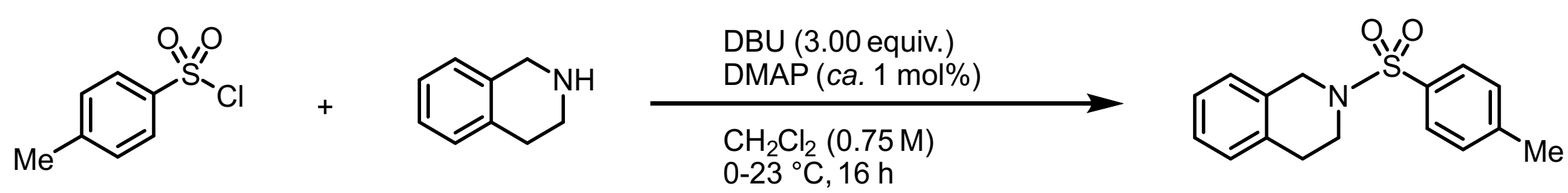

In a 50 mL single-necked round-bottom flask equipped with a magnetic stir bar, 1,2,3,4-tetrahydroisoquinoline (1.01 g, 7.50 mmol, 1.50 equiv.) was dissolved in CH_2Cl_2 (10 mL, 0.75 M). DBU (2.24 mL, 15.0 mmol, 3.00 equiv.) was added and the reaction was cooled to 0 °C. Tosyl chloride (963 mg, 5.00 mmol, 1.00 equiv.) was then added, followed by a microspatula tip of DMAP. The reaction was allowed to warm slowly to r.t. overnight, then quenched with 1 M HCl. The aqueous mixture was extracted with CH_2Cl_2 (3x 20 mL), and the combined organic phases were washed sequentially with sat. aq. $NaHCO_3$ (20 mL) and water (20 mL), then dried over $MgSO_4$, filtered over cotton, and concentrated by rotary evaporation under reduced pressure. The crude residue was purified by flash column chromatography (heptane/EtOAc) to afford the title compound as a colourless solid in 37% yield (794 mg, 2.76 mmol).

R*f* = 0.42 (3:1 heptane/EtOAc)

NMR Spectroscopy:

> **^{1}H NMR** (400 MHz, $CDCl_3$, 23 ºC, δ): 7.76–7.69 (m, 2H), 7.35–7.30 (m, 2H), 7.18–7.11 (m, 2H), 7.11–6.98 (m, 2H), 4.25 (s, 2H), 3.35 (t, *J* = 5.9 Hz, 2H), 2.93 (t, *J* = 5.9 Hz, 2H), 2.42 (s, 3H).
>
> **^{13}C NMR** (101 MHz, $CDCl_3$, 23 ºC, δ): 143.8, 133.4, 133.2, 131.8, 129.8, 128.9, 127.9, 126.9, 126.5, 126.5, 47.7, 43.9, 29.0, 21.7.

HRMS-ESI(m/z) calc'd for $C_{16}H_{18}NO_2S$ $[M+H]^+$, 288.1053; found, 288.1053; deviation: 0.0 ppm.

ConPET catalysis

Light source for photoreactions

EvoluChem 18W LEDs (390PF) were used for photoreactions. The LED emission spectrum is shown in **Figure S1**.

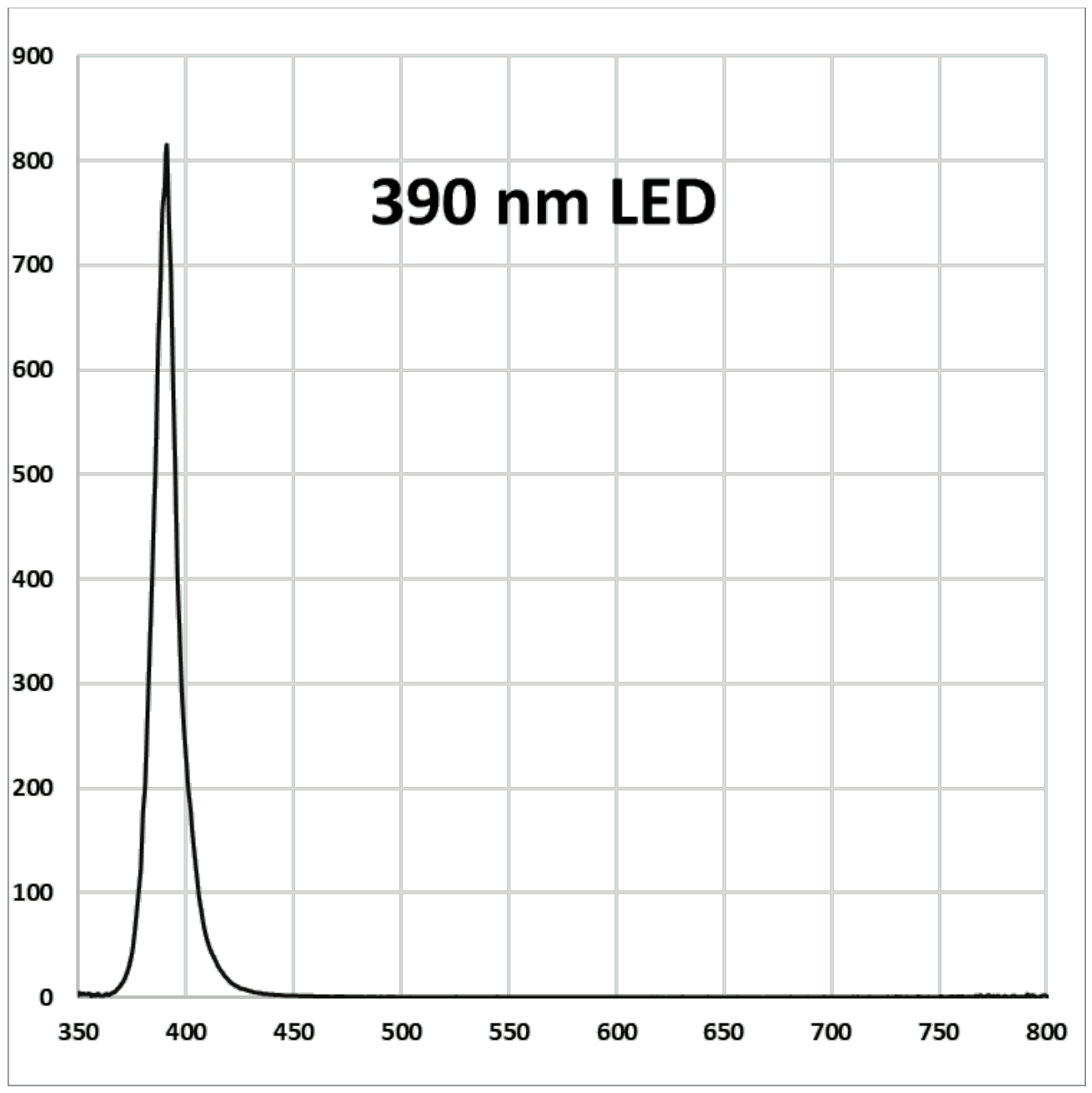

Figure S1. LED emission spectrum, measured by the manufacturer. The original emission spectra can be accessed on the following link (accessed on 03/09/2026): https://hepatochem.com

Photoreactor configuration

Pictures of the 3D printed photoreactor used for the reactions carried out in 4 mL scintillation vials are shown in **Figure S2**. During the experiments the temperature in the inner space of the reactor was 27±1 °C with a working cooling fan and 39±1 °C without cooling.

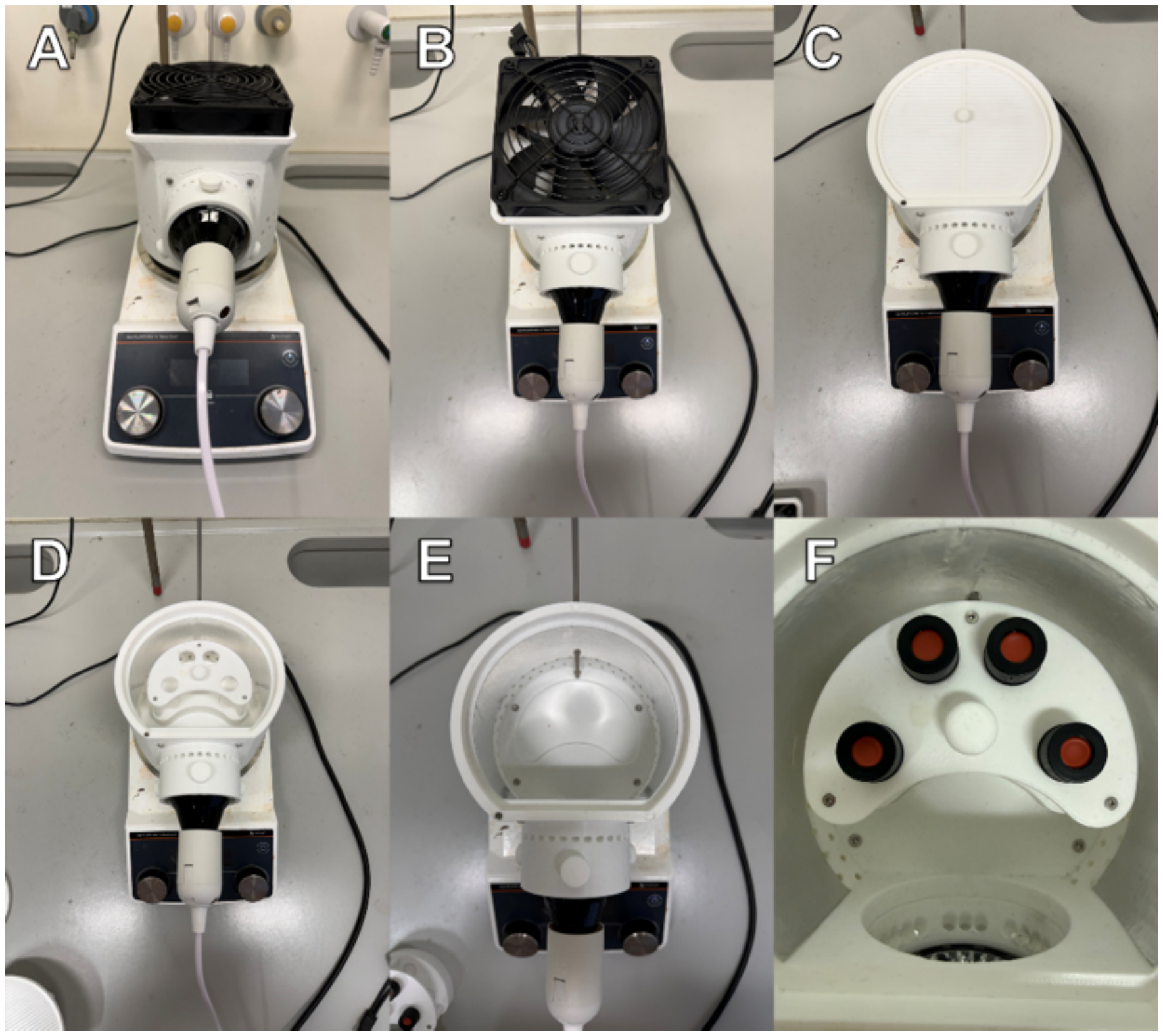

Figure S2. Photoreactor used for reactions in 4 mL borosilicate scintillation vials: **A.** Front view of reactor ready for usage; **B.** Top view of reactor ready for usage; **C.** Reactor without a cooling fan; **D.** Reactor without protecting screen; **E.** View of the reactor without the vial stand. The image also shows the position of the probe for temperature control; **F.** Inner space of the reactor equipped with four 4 mL borosilicate scintillation vials.

General procedure for conPET photocatalysis

A 4 mL scintillation vial was charged sequentially with tosylamide (1.00 equiv.), **1(BF_4)** (10.0 mol%), a teflon-lined magnetic stir bar, CH_3CN (0.1 M), and DIPEA (3.00 equiv.). The vial was closed with a screw cap containing a rubber septum insert, then sparged with dry argon (5 mins) via an inlet needle and bleed needle, both inserted through the septum. The bleed and inlet needles were sequentially removed, and the vial was sealed with PVC electrical tape. The reaction mixture was irradiated with a 390 nm 18 W LED lamp for 20 h while stirring at 1000 rpm. The vial was then unsealed and the reaction mixture loaded onto silica with washings of CH_2Cl_2, then purified by flash column chromatography on silica gel.

Characterization data of conPET products

*Benzimidazole (**3**)*

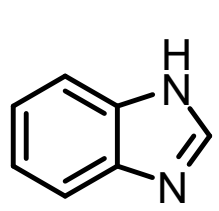

Synthesized according to the General procedure for conPET photocatalysis on 0.200 mmol scale, and isolated by flash column chromatography (Et_2O/MeOH) as a colourless solid in 90% yield (21.3 mg, 0.180 mmol).

R*f* = 0.21 (100:1 Et_2O/MeOH).

NMR Spectroscopy:

^{1}H NMR (400 MHz, DMSO-d6, 23 ºC, δ): 8.21 (s, 1H), 7.63–7.52 (m, 2H), 7.19 (dt, *J* = 6.0, 3.2 Hz, 2H).

^{13}C NMR (101 MHz, DMSO-d6, 23 ºC, δ): 141.9, 138.0, 121.7, 115.3.

HRMS-ESI(m/z) calc'd for $C_7H_5N_2$ $[M–H]^-$, 117.0458; found, 117.0458; deviation: 0.0 ppm.

*Indole (**4**)*

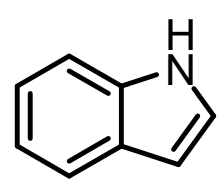

Synthesized according to the General procedure for conPET photocatalysis on 0.200 mmol scale, and isolated by flash column chromatography (pentane/Et_2O) as a colourless solid in 87% yield (20.5 mg, 0.175 mmol).

R*f* = 0.50 (3:1 pentane/Et_2O).

NMR Spectroscopy:

^{1}H NMR (400 MHz, $CDCl_3$, 23 ºC, δ): 8.11 (s, 1H), 7.71 (dq, *J* = 7.8, 1.0 Hz, 1H), 7.43 (dq, *J* = 8.1, 1.1 Hz, 1H), 7.30–7.21 (m, 2H), 7.18 (ddd, *J* = 8.1, 7.0, 1.1 Hz, 1H), 6.61 (ddd, *J* = 3.1, 2.1, 1.0 Hz, 1H).

^{13}C NMR (151 MHz, DMSO-d6, 23 ºC, δ): 135.8, 127.6, 125.2, 120.8, 120.0, 118.7, 111.4, 100.9.

HRMS-ESI(m/z) calc'd for C_8H_6N $[M–H]^-$, 116.0506; found, 116.0504; deviation: –1.7 ppm.

*1*H-*Indole-3-carbonitrile (**5**)*

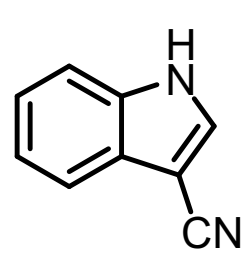

Synthesized according to the General procedure for conPET photocatalysis on 0.200 mmol scale, and isolated by flash column chromatography (heptane/EtOAc) as a light brown solid in 82% yield (23.4 mg, 0.165 mmol).

R*f* = 0.079 (4:1 heptane/EtOAc).

NMR Spectroscopy:

^{1}H NMR (400 MHz, DMSO-d6, 23 ºC, δ): δ 12.19 (s, 1H), 8.24 (d, *J* = 2.9 Hz, 1H), 7.63 (dd, *J* = 7.4, 1.4 Hz, 1H), 7.55 (dt, *J* = 8.1, 1.1 Hz, 1H), 7.28 (ddd, *J* = 8.2, 7.1, 1.4 Hz, 1H), 7.23 (td, *J* = 7.4, 1.1 Hz, 1H).

^{13}C NMR (101 MHz, DMSO-d6, 23 ºC, δ): 135.2, 134.5, 126.7, 123.3, 121.6, 118.4, 116.4, 112.9, 84.2.

HRMS-ESI(m/z) calc'd for $C_9H_6N_2Na$ $[M+Na]^+$, 165.0423; found, 165.0423; deviation: 0.0 ppm.

*Indazole (**6**)*

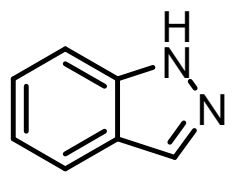

Synthesized according to the General procedure for conPET photocatalysis on 0.100 mmol scale, and isolated by flash column chromatography (heptane/EtOAc) as a light brown solid in 81% yield (9.60 mg, 0.0813 mmol).

R*f* = 0.48 (1:1 heptane/EtOAc).

NMR Spectroscopy:

^{1}H NMR (400 MHz, $CDCl_3$, 23 ºC, δ): 8.14 (s, 1H), 7.78 (dt, *J* = 8.2, 1.0 Hz, 1H), 7.52 (dd, *J* = 8.4, 1.0 Hz, 1H), 7.41 (ddd, *J* = 8.2, 6.9, 1.0 Hz, 1H), 7.23–7.14 (m, 1H).

^{13}C NMR (101 MHz, $CDCl_3$, 23 ºC, δ): 140.2, 135.0, 127.0, 123.4, 121.2, 121.1, 109.9.

HRMS-ESI(m/z) calc'd for $C_7H_7N_2$ $[M+H]^+$, 119.0604; found, 119.0607; deviation: +2.5 ppm.

*Carbazole (**7**)*

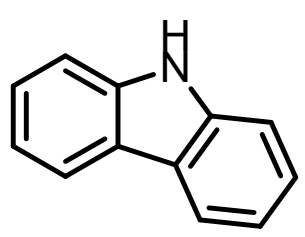

Synthesized according to the General procedure for conPET photocatalysis on 0.200 mmol scale, and isolated by flash column chromatography (heptane/EtOAc) as a colourless solid in 91% yield (30.3 mg, 0.181 mmol).

R*f* = 0.38 (5:1 heptane/EtOAc).

NMR Spectroscopy:

^{1}H NMR (600 MHz, DMSO, 23 ºC, δ) 11.23 (s, 1H), 8.10 (dd, *J* = 7.7, 1.2 Hz, 2H), 7.50 – 7.45 (m, 2H), 7.37 (ddd, *J* = 8.1, 7.1, 1.2 Hz, 2H), 7.15 (ddd, *J* = 8.1, 7.1, 1.0 Hz, 2H).

^{13}C NMR (151 MHz, DMSO-d6, 23 ºC, δ): 139.7, 125.5, 122.4, 120.1, 118.4, 110.9.

HRMS-ESI(m/z) calc'd for $C_{12}H_{10}N$ $[M+H]^+$, 168.0808; found, 168.0808; deviation: 0.0 ppm.

Limitations

Aliphatic *N*-tosylamines were found to be unreactive under the standard conPET photocatalytic conditions specified above, as were *N*-tosyl- and *N*,*N*-ditosylanilines. These substrate classes reportedly detosylate in high yields under acridinyl conPET catalysis.[10] This reactivity difference is consistent a less negative excited-state oxidation potential for the selenoxanthylyl radical compared with the acridinyl radical.

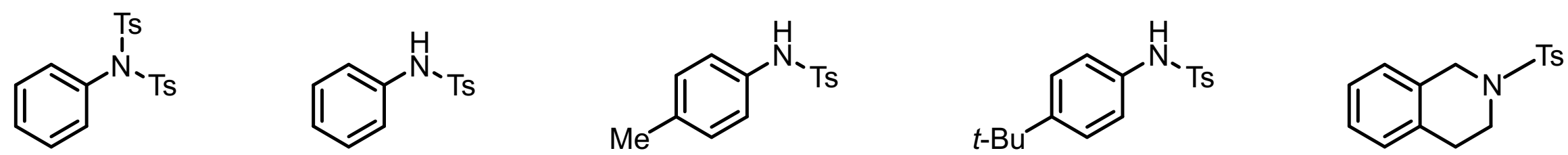

S6 **S7** **S8** **S9** **S10**

Similarly, irradiation of an equimolar mixture of radical **2** and 4-chlorobenzonitrile, in the absence of a sacrificial reductant but under otherwise unchanged conditions, did not afford the dehalogenation product in detectable quantities.

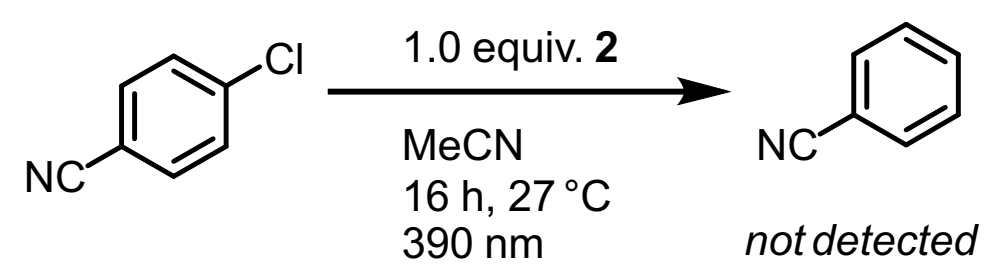

In the presence of 3 molar equivalents of DIPEA, the NMR yield of dechlorination could be improved to 15%. However, the absence of dehalogenation without DIPEA and the possibility of a competing halogen-atom transfer (XAT) pathway prevent us from assigning the reaction unequivocally to a conPET mechanism.

SELENOXANTHYLYL RADICAL SYNTHESIS

3,6-Di-tert-butyl-9-mesitylxanthylyl radical (2)

Procedure 1

The following procedure was used for EPR and UV-vis studies of radical **2**.

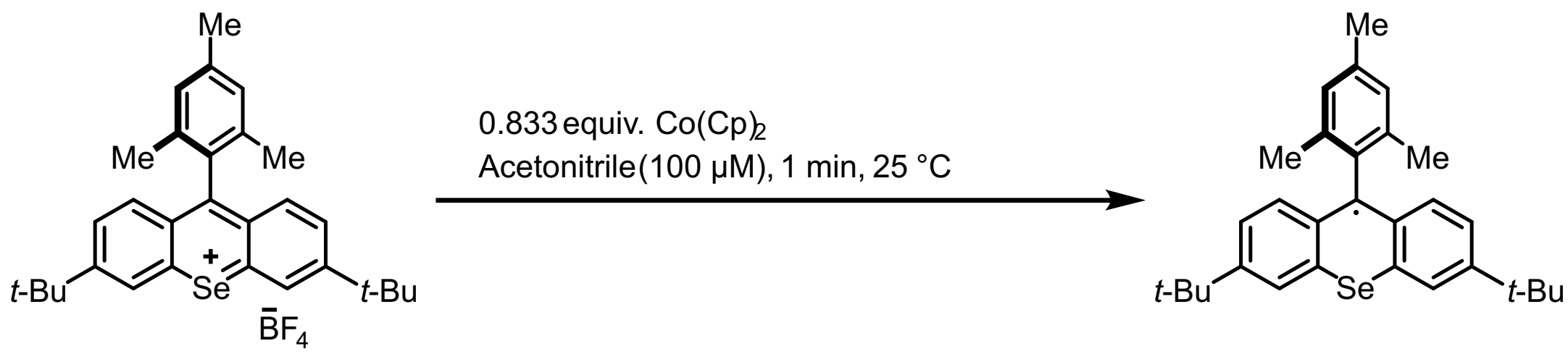

1(BF$_4$) **2**

In an argon-filled glovebox, cobaltocene (9.55 mg, 2.00 equiv., 50.0 μmol) and selenoxanthylium **1(BF$_4$)** (14.0 mg, 1.00 equiv., 25.0 μmol) were each dissolved in 2.50 mL dry, degassed acetonitrile (see *Materials and Methods* section) to give 20.0 mM and 10.0 mM stock solutions, respectively. The solution of **1(BF$_4$)** was diluted to 100 μM by making 60.0 μL (600 nmol) up to 6.00 mL. Cobaltocene solution (25.0 μL, 500 nmol) was then added to the diluted solution of **1(BF$_4$)**, and the resulting mixture was swirled gently. A change of colour from crimson to light yellow was immediately observed. The solution was then diluted with further dry, degassed acetonitrile as appropriate for EPR and UV-vis studies.

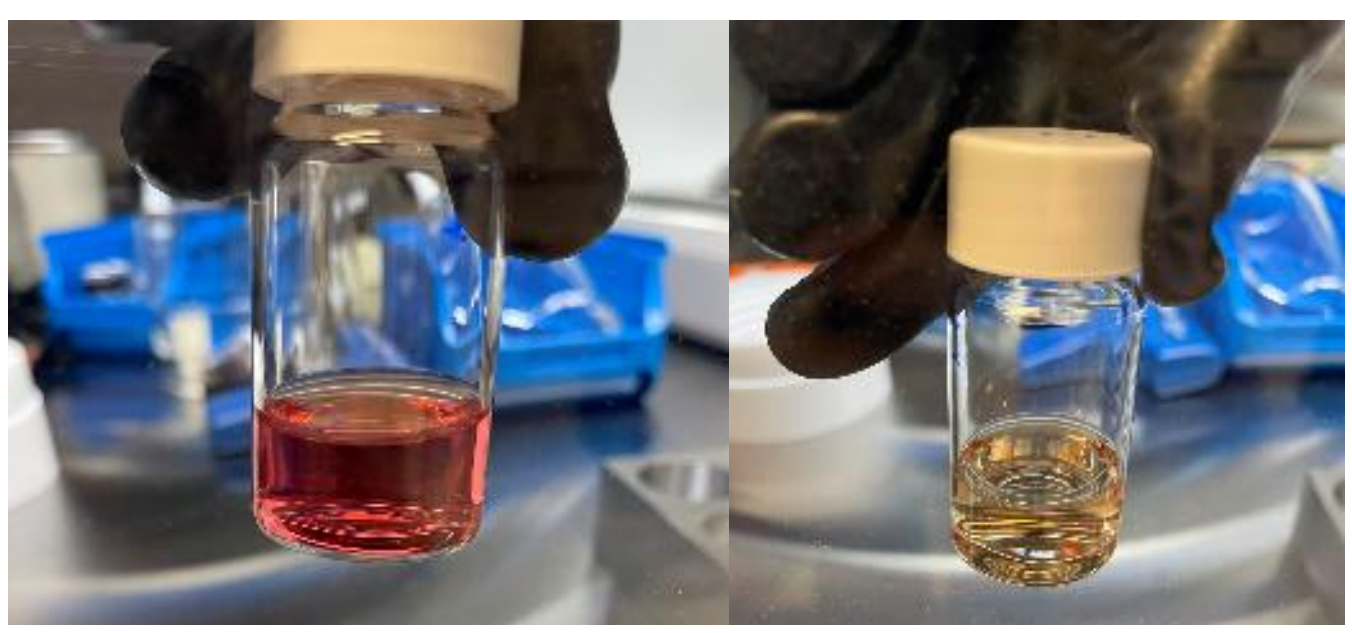

Figure S3. *Left*: 100 μM solution of **1(BF$_4$)** in acetonitrile. *Right*: 100 μM solution of **2** in acetonitrile, resulting from reduction of **1(BF$_4$)** with cobaltocene. The characteristic colour change is visible.

Procedure 2

The EPR signature of the solution resulting from the following procedure was identical to that observed with *Procedure 1*.

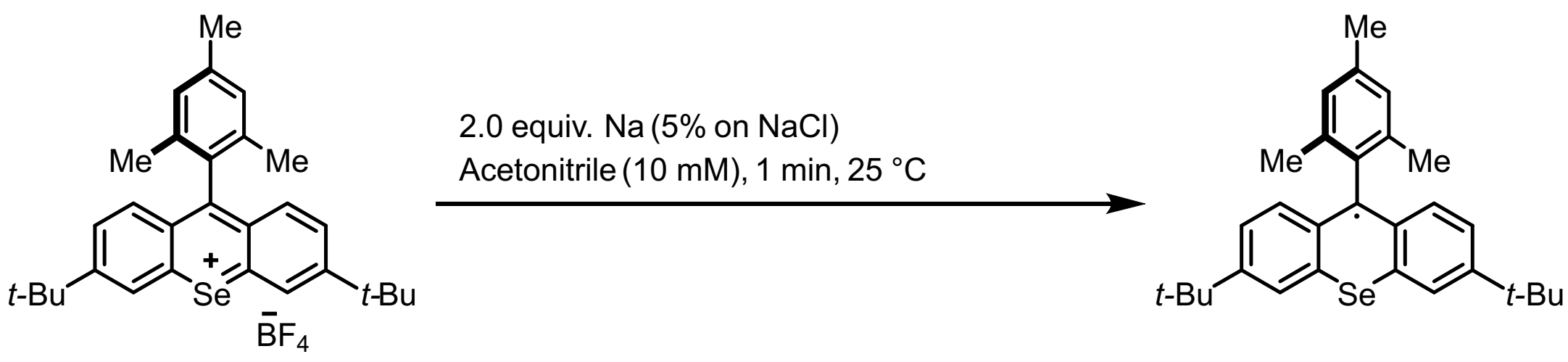

Preparation of Na/NaCl

A 5% wt/wt dispersion of sodium on sodium chloride was prepared using a slightly modified version of the procedure reported by Jones and coworkers.[12] Sodium chloride was oven-dried for 3 days at 130 °C, then ground to a fine powder, using a domestic kitchen blender over *ca.* 30 min. A portion of the resulting powder (80 g) was subsequently transferred to a 1 L Schlenk flask containing a large magnetic stir bar and dried under vacuum, with stirring, for 3 h at *ca.* 300 °C (using a heat gun; approximate temperature determined using a hand-held infrared thermometer). The flask was allowed to cool under vacuum and then refilled with dry argon. Freshly cut sodium metal (4.00 g, 174 mmol) was added, and the flask was gently heated to *ca.* 125 °C with constant stirring. Once the sodium melted, rapid stirring and agitation continued until no bulk metal was visible, leaving a freely flowing pale grey powder which was allowed to cool to room temperature, transferred to an argon-filled glovebox, and stored there at room temperature before use (yield 84 g).

Reduction using Na/NaCl

In an argon-filled glovebox, an oven-dried 4 mL scintillation vial was charged sequentially with selenoxanthylium **1(BF$_4$)** (7.0 mg, 1.0 equiv., 12.5 μmol) and dry, degassed acetonitrile (1.25 mL, 10 mM). Upon complete dissolution of the solids, Na/NaCl (5% wt/wt, 11.5 mg, 25 μmol, 2.0 equiv.) was added in a single portion and the reaction mixture was stirred with a stainless steel microspatula for 1 minute, by which time the colour of the mixture had changed from crimson to light brown. A filter was prepared by compacting a round of oven-dried glass fibre filter paper (MN GF-1, 0.7 μm retention) in the shoulder of an oven-dried glass Pasteur pipette. The solid residues were removed by gravity filtration through this filter, eluting with further dry, degassed acetonitrile to ensure quantitative transfer of the selenoxanthylyl radical. The resulting solution was light brown, turning bronze upon dilution to 10 μM with further acetonitrile.

SELENOXANTHYLYL CHARACTERIZATION

CYCLIC VOLTAMMETRY

Reduction – Redox couple ([4a]$^+$/[4a]$^·$)

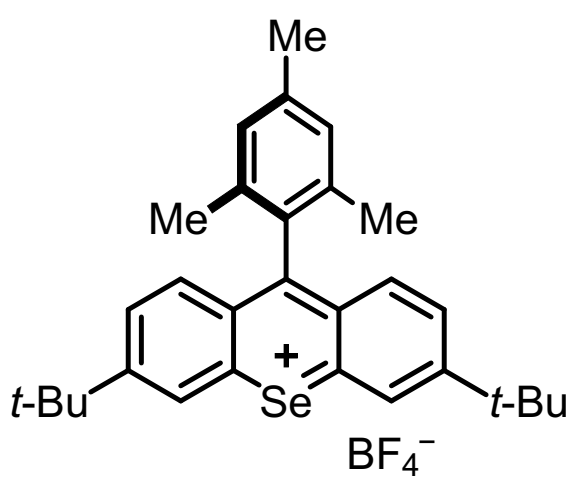

1(BF$_4$)

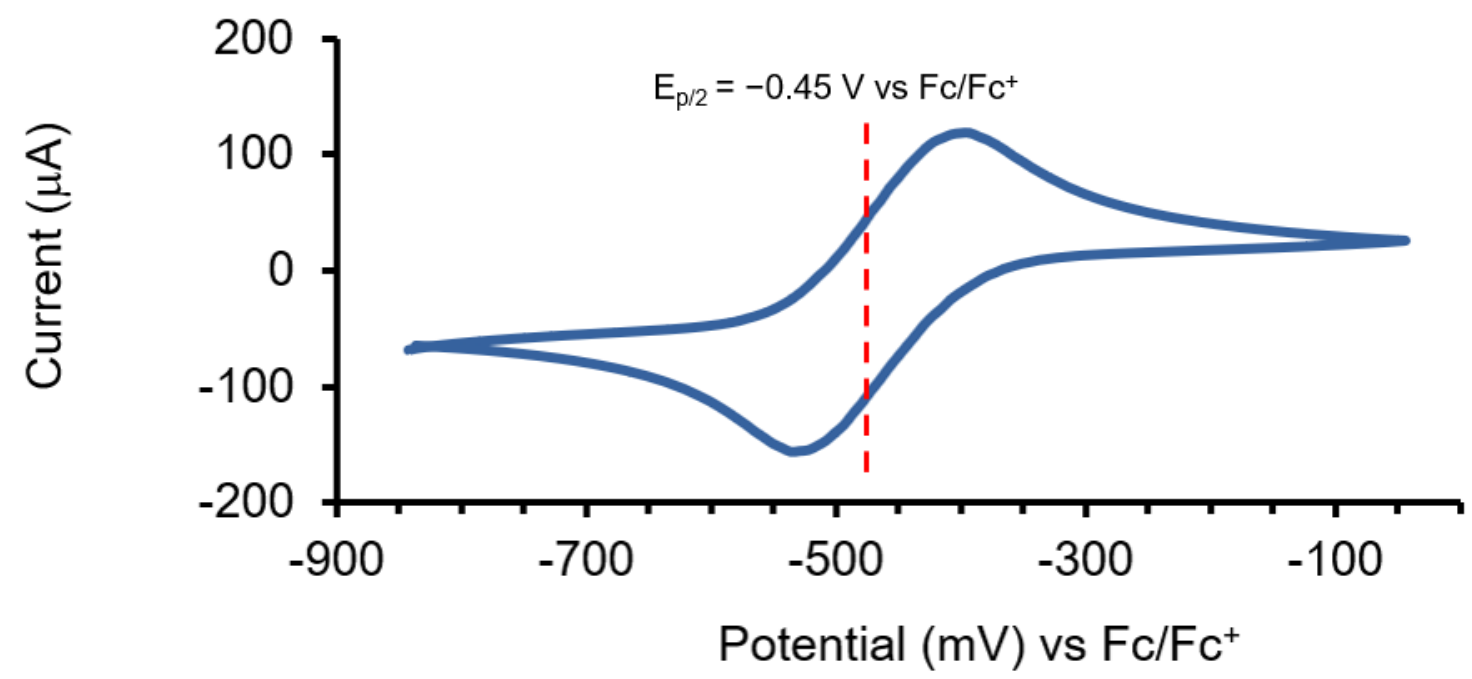

Figure S4. Cathodic CV of **1(BF$_4$)** in a 0.10 M NBu_4PF_6 CH_3CN solution. GC electrode. Scan rate: 100 mV/s, potential referred to Ag/$AgNO_3$ (0.10 M in CH_3CN) using Fc/Fc$^+$ as internal reference at 298 K with a platinum disk as counter electrode.

$E_{p/2}$ = −0.45 V vs Fc/Fc$^+$ = −0.07 V vs. SCE (in CH_3CN)

EPR SPECTROSCOPY

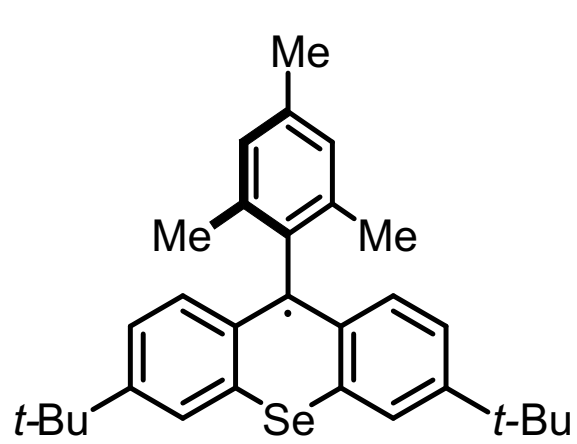

2

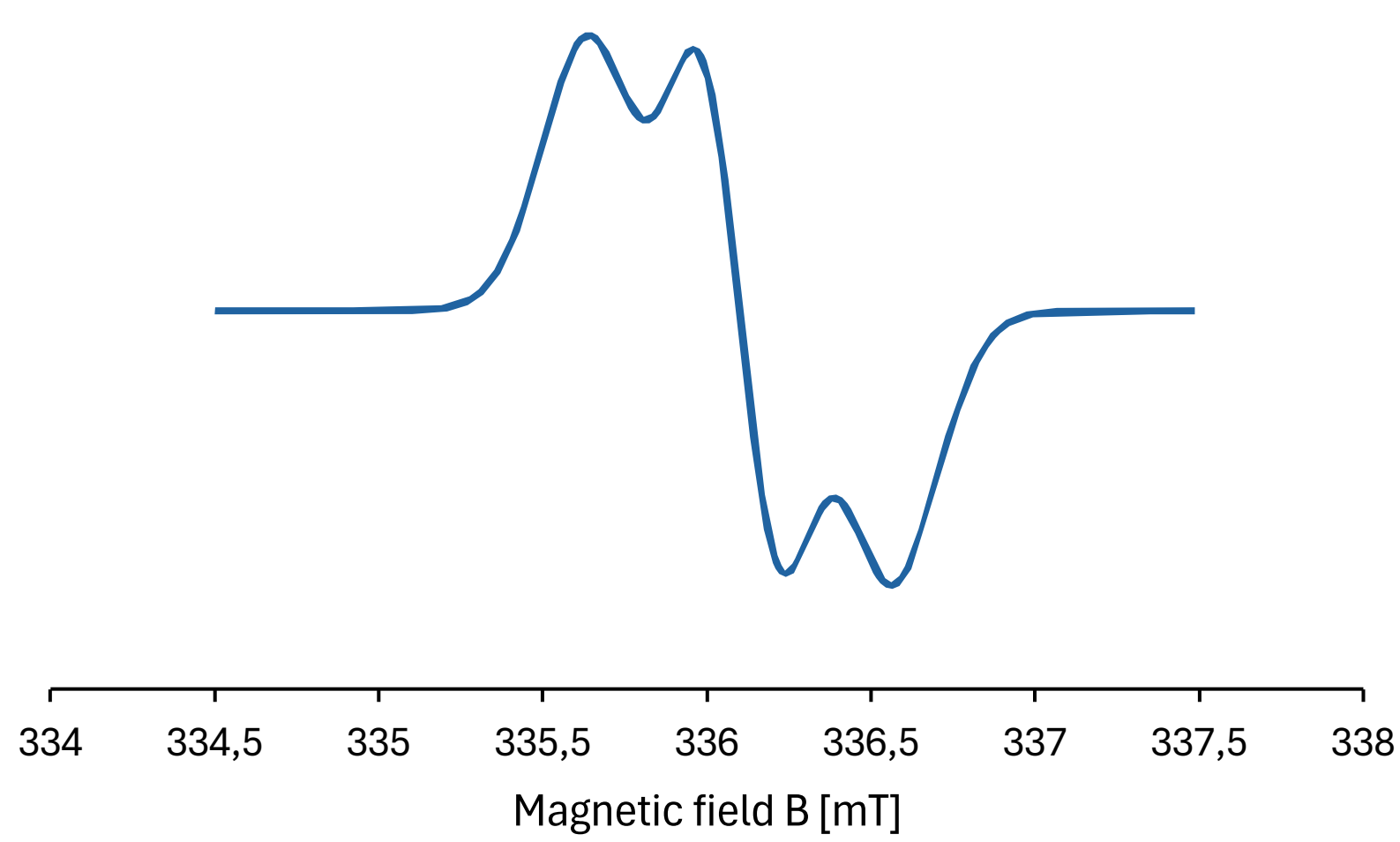

Figure S5. EPR spectrum of **2** in a 10 μM CH_3CN solution, recorded over a magnetic-field range of 334.5–337.5 mT with a sweep time of 60 s and 30 scans per spectrum.

The EPR spectrum was simulated using EasySpin (garlic) with g = 2.0057, hyperfine coupling to two equivalent ^{1}H nuclei (A = 9.284 MHz), a peak-to-peak linewidth of 0.32 mT, and a microwave frequency of 9.45 GHz.

The MATLAB code used for the simulation is as follows:

```
Sys.g = 2.0057;
Sys.Nucs = '1H';
Sys.n = 2;
Sys.A = 9.284;        % MHz
Sys.lwpp = 0.32;      % mT

Exp.mwFreq = 9.45;    % GHz
Exp.Range = [335 338]; % mT
Exp.nPoints = 2000;

[B,spc] = garlic(Sys,Exp);
plot(B,spc);
```

Reoxidation study

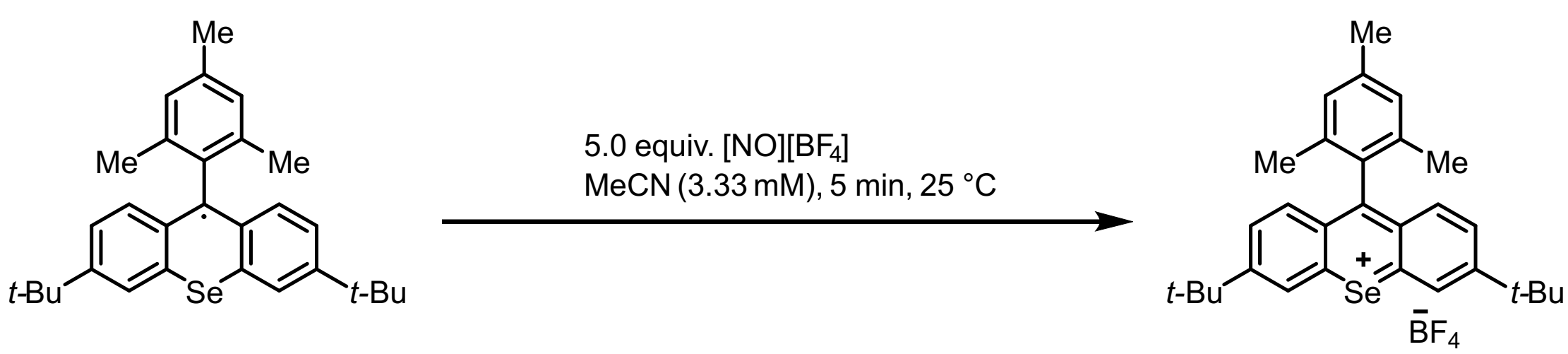

2 **1(BF$_4$)**

In an argon-filled glovebox, 6.67 mM solution of radical **2** (prepared by reduction of **1(BF$_4$)** with cobaltocene, *vide supra*) was diluted to 3.33 mM with acetonitrile-d3 and transferred to an oven-dried J-Young tube. The sample was an orange-brown solution, and its ^{1}H NMR spectrum was almost silent in the aromatic region. The sample was then transferred to a vial and treated with nitrosonium tetrafluoroborate (5.0 equiv.) for 5 min, during which time a change of colour from orange-brown to scarlet was observed. The sample was returned to the NMR tube and remeasured, and its ^{1}H NMR spectrum was found to contain the characteristic aromatic signals of **1(BF$_4$)**.

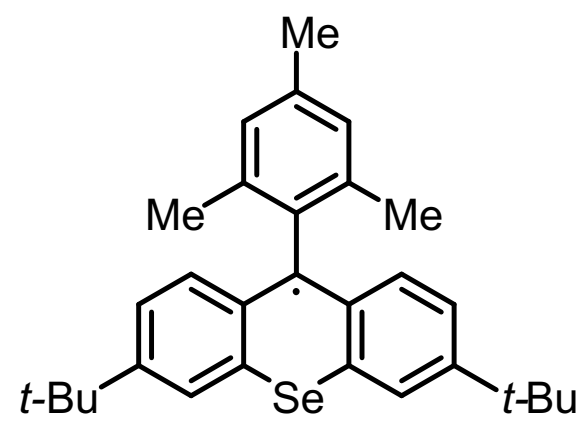

2

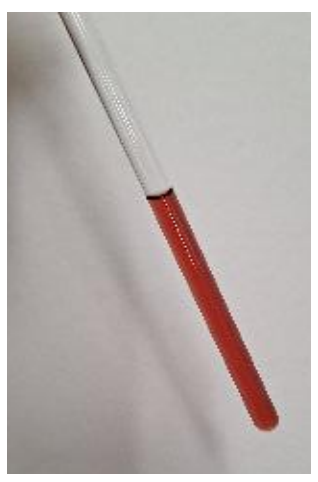

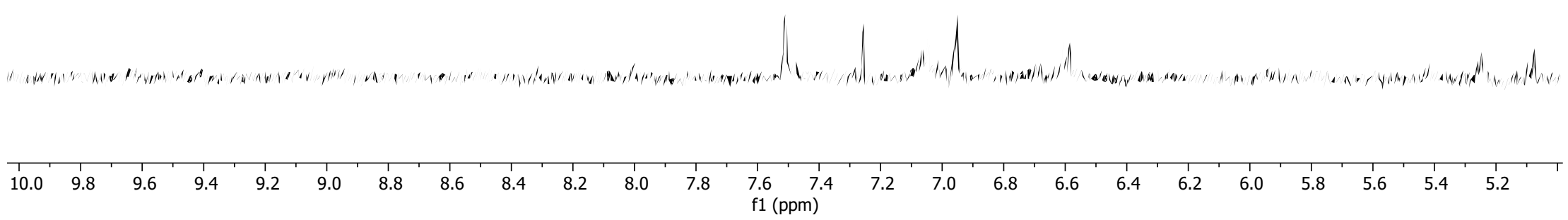

Figure S6. NMR sample of **2** and detail of ^{1}H NMR spectrum at 3.3 mM.

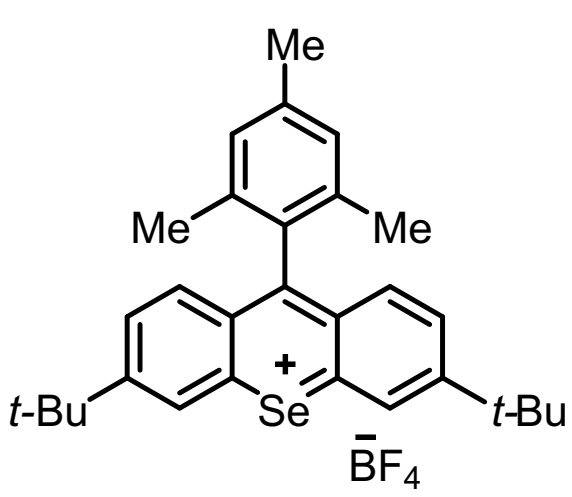

1(BF$_4$)

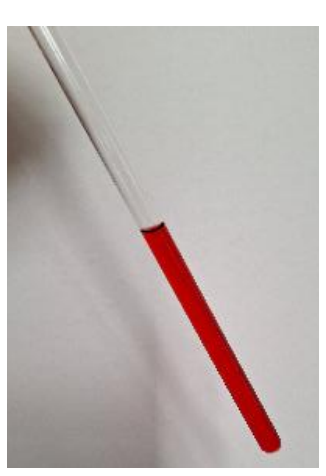

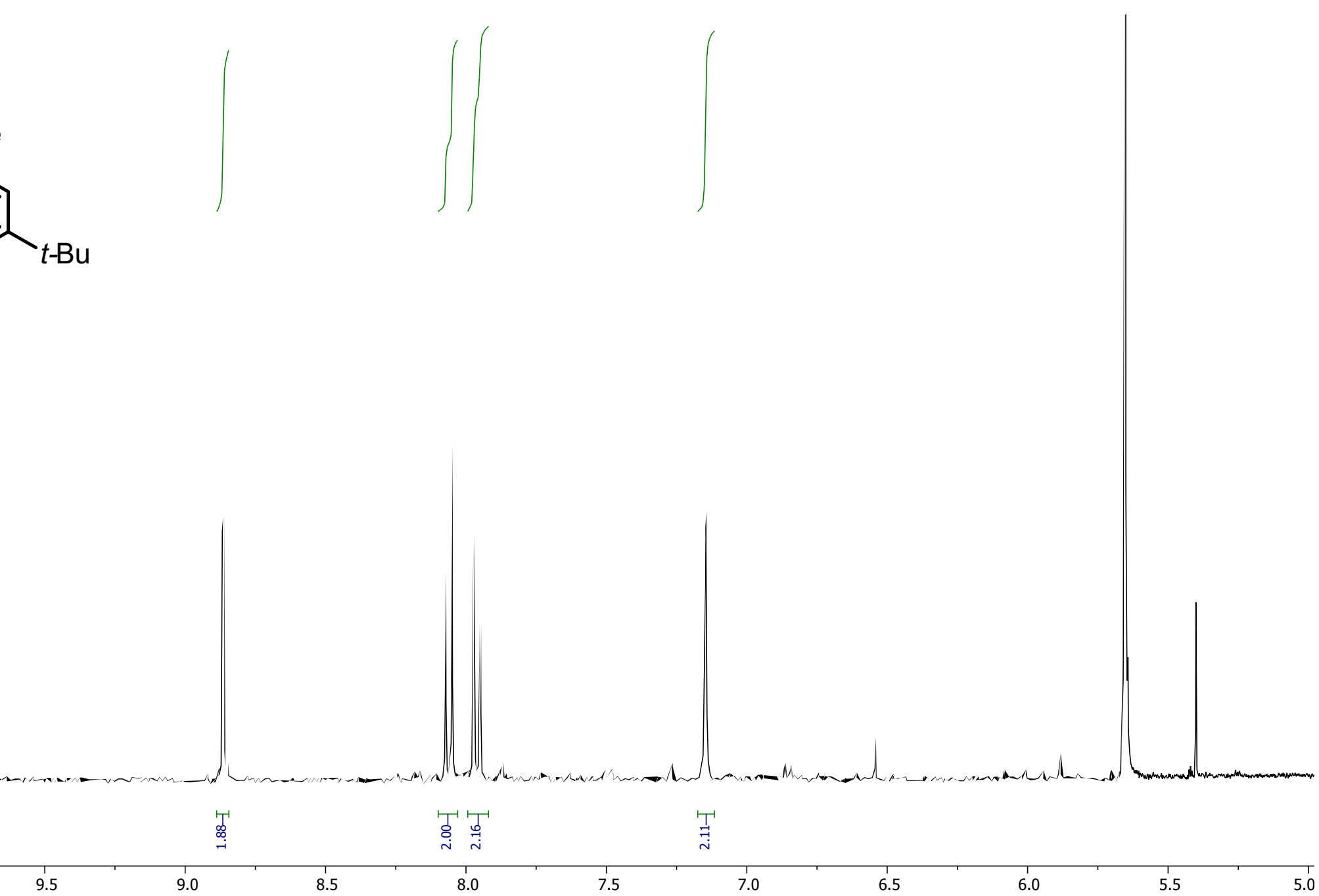

Figure S7. NMR sample of **1(BF$_4$)**, formed *in situ* by oxidation of **2** with nitrosonium tetrafluoroborate, and detail of ^{1}H NMR spectrum at 3.3 mM.

SPECTROPHOTOMETRY

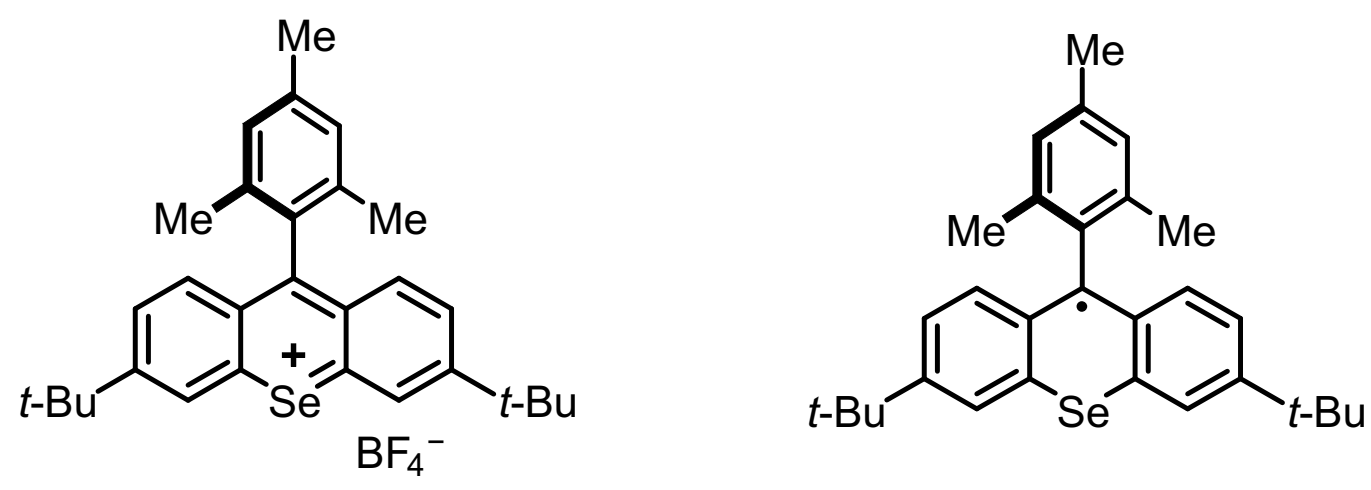

1(BF₄) **2**

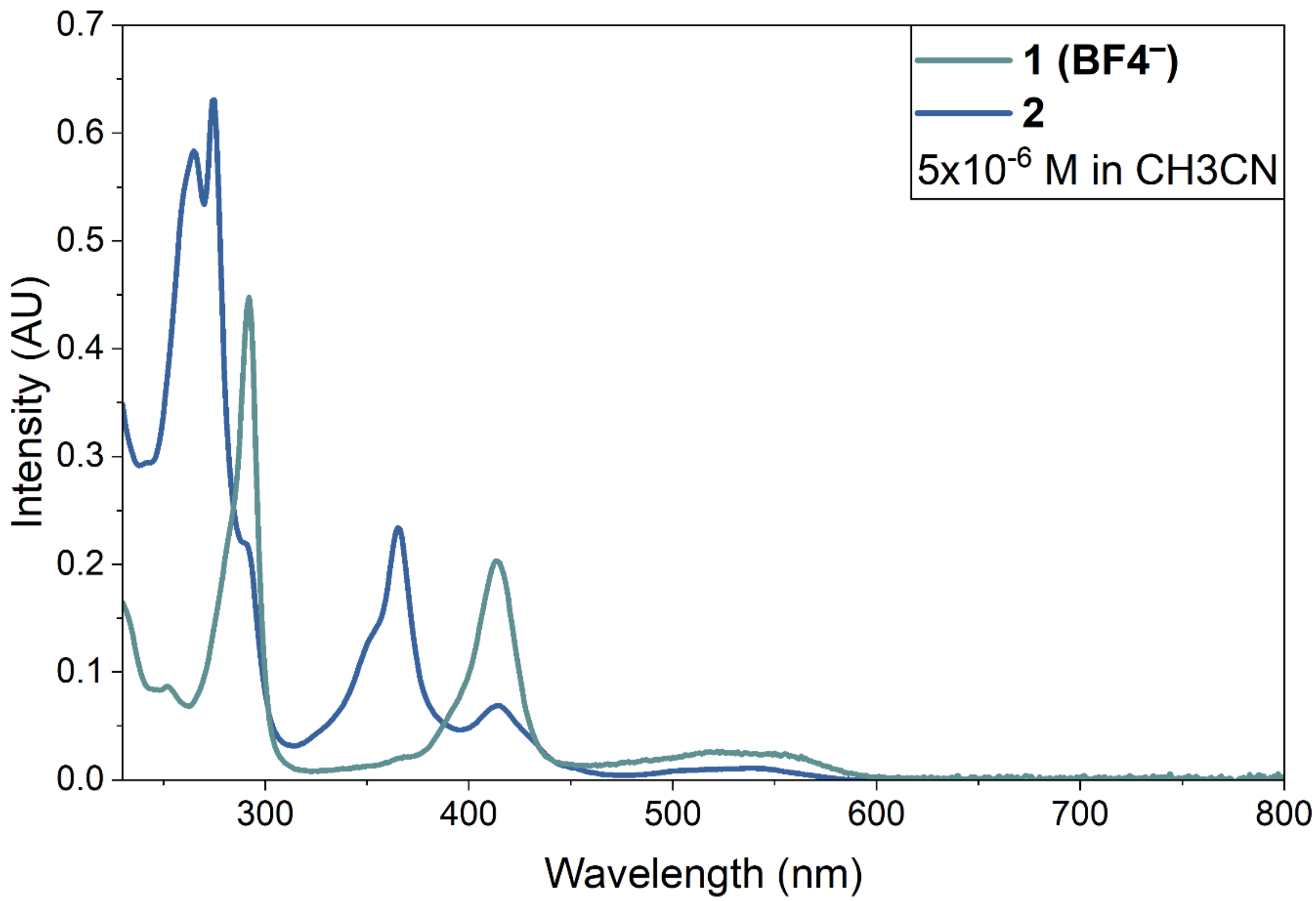

Figure S8. Absorption of **1 (BF_4)** (green traces) and **2** (blue traces) at concentration of $5 \cdot 10^{-6}$ M in CH_3CN.

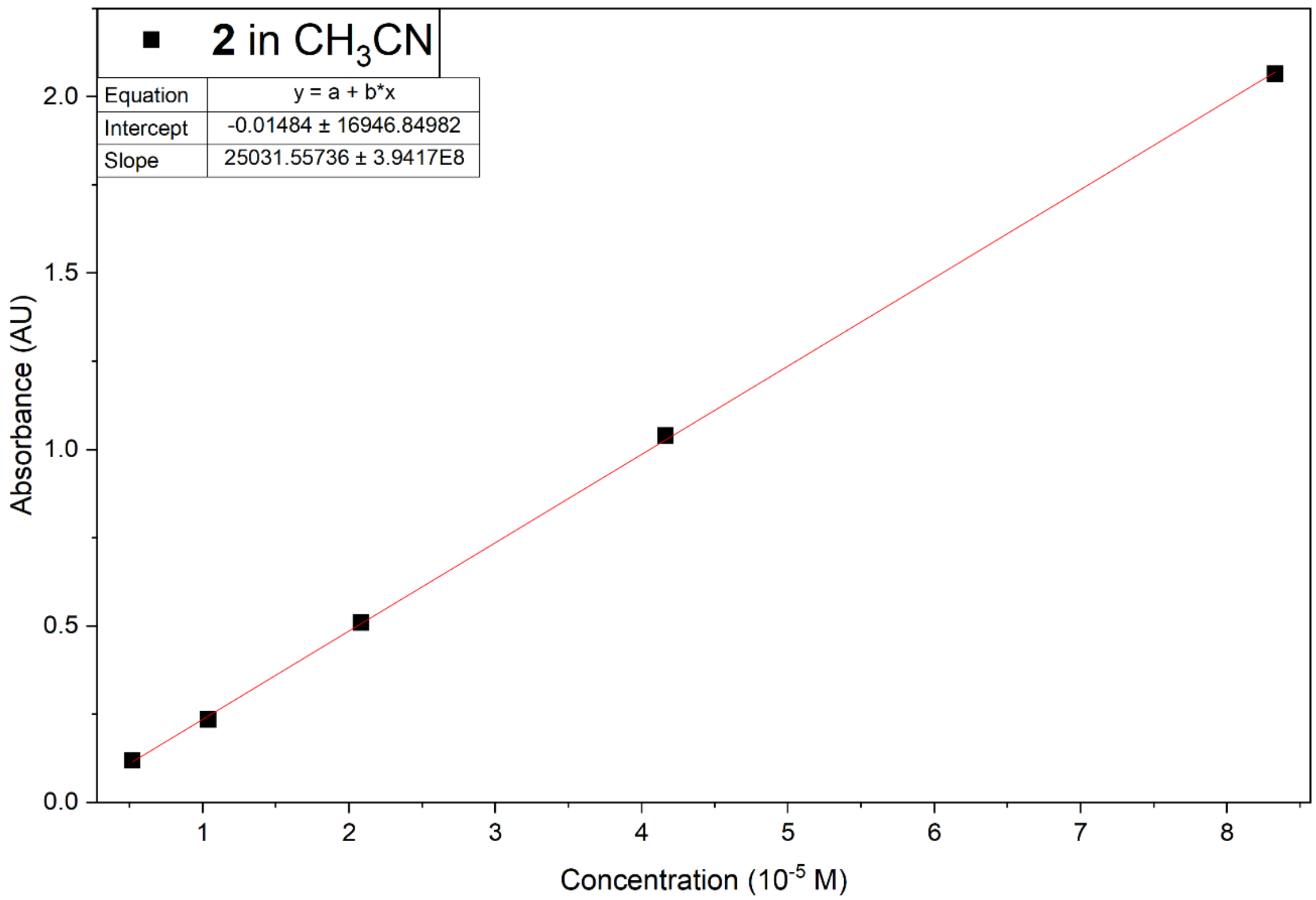

Figure S9. Linear fit of absorbances of **2** measured at 365 nm in CH_3CN.

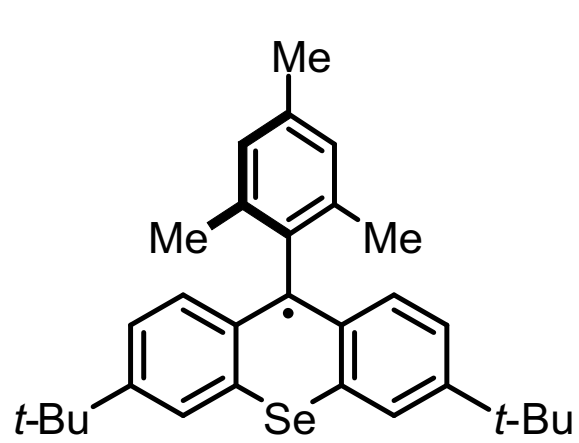

2

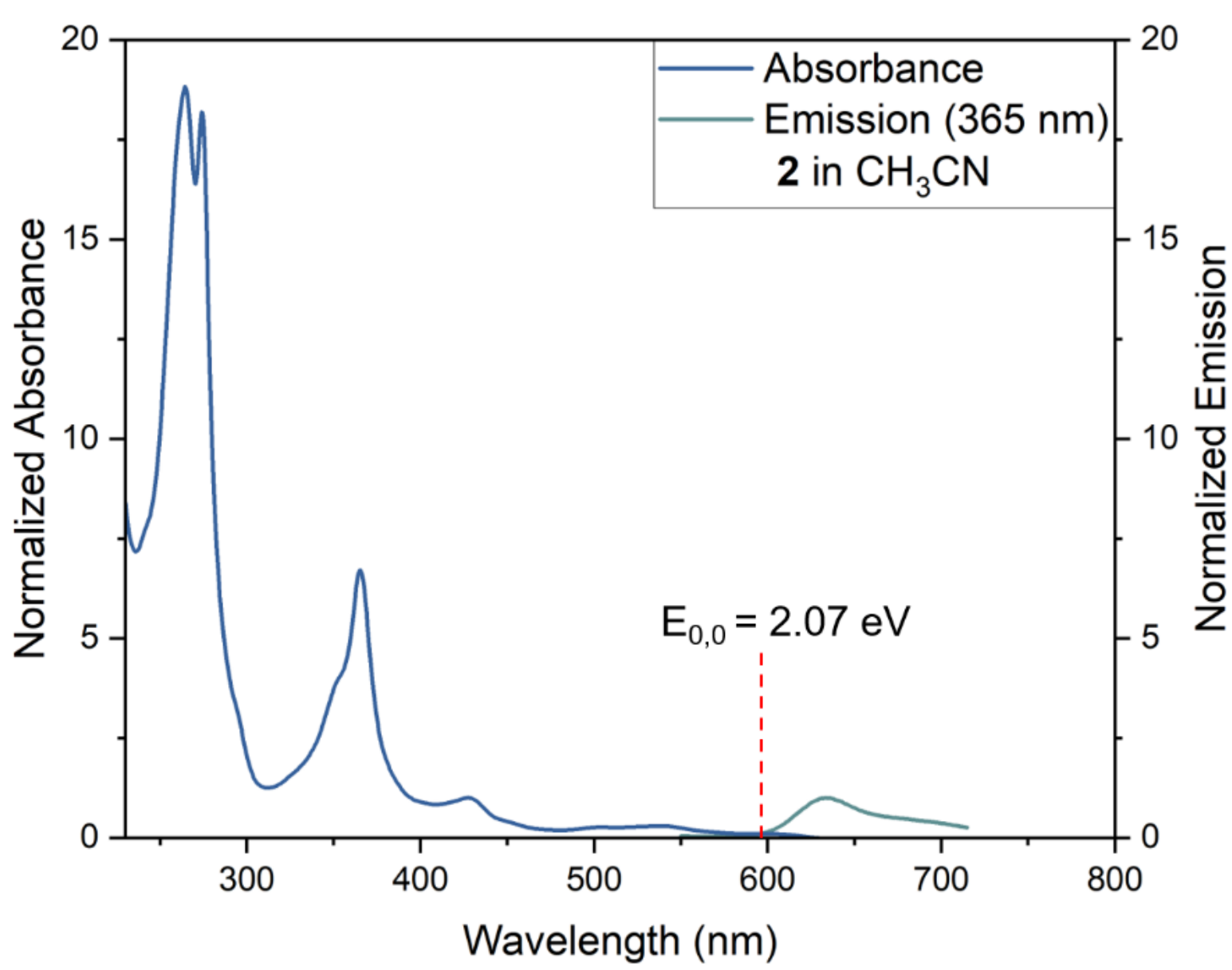

Figure S10. Absorbance (blue traces) and emission (excitation λ = 365 nm) spectrum of **2** in CH_3CN.

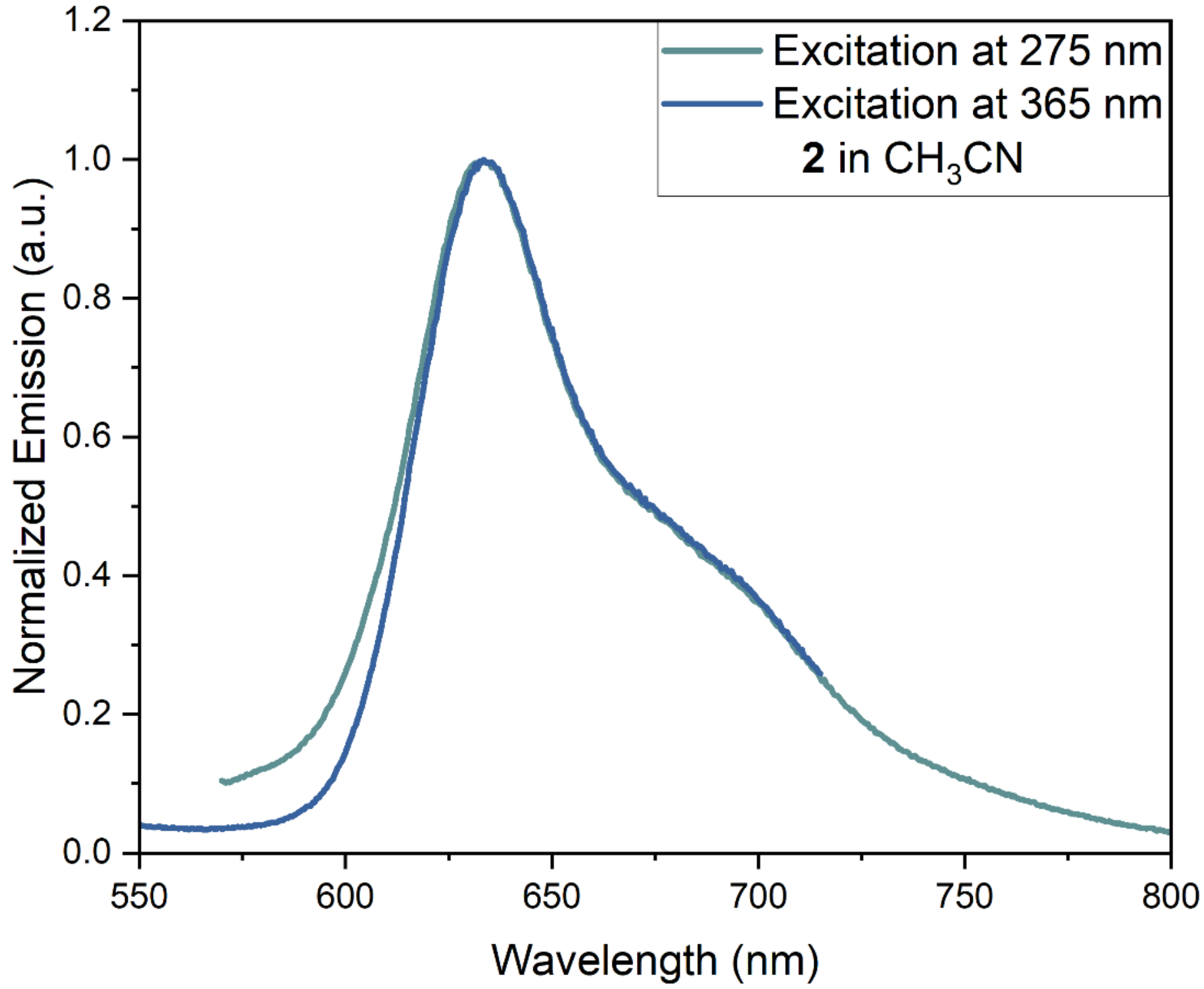

Figure S11. Emission spectra with excitation at λ = 275 nm (green traces) and λ = 365 nm (blue traces) spectrum of **2** in CH_3CN.

Summary

ε (365 nm) = 25031 M^{-1} cm^{-1}

E_{ox} (**[4a]**$^{\cdot}$/**[4a]**$^{*+}$) = −0.07 V vs SCE

E^{*}_{ox} (**[4a]**$^{\cdot}$/**[4a]**$^{*+}$) = −0.07 V – 2.07 V = **−2.14 V vs SCE**

Computational Data

Comparison between TD-DFT and experimental absorption spectra

Selenoxanthylyl radical 2

Optimized structure

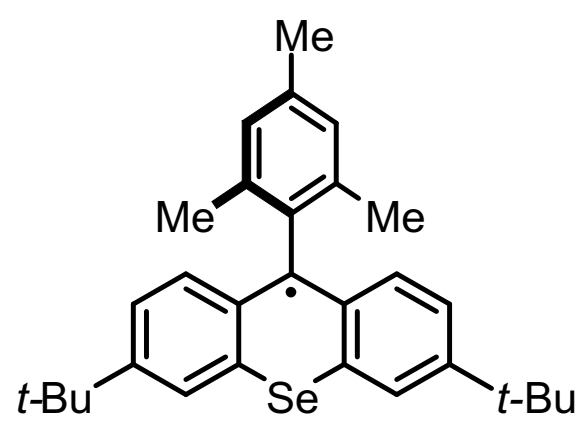

2

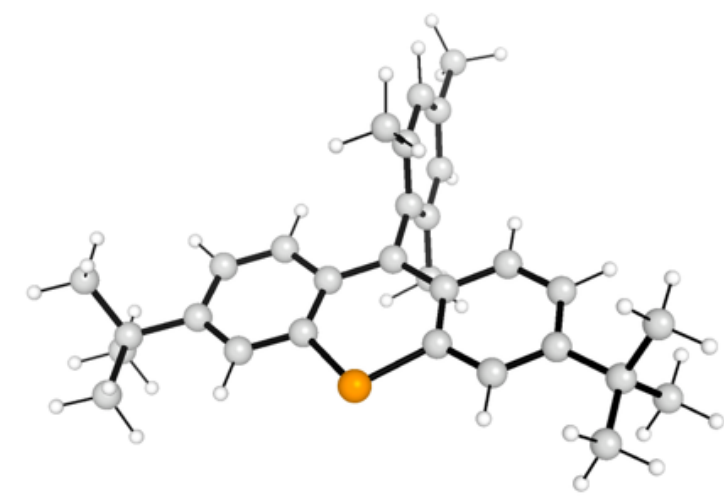

Spin density

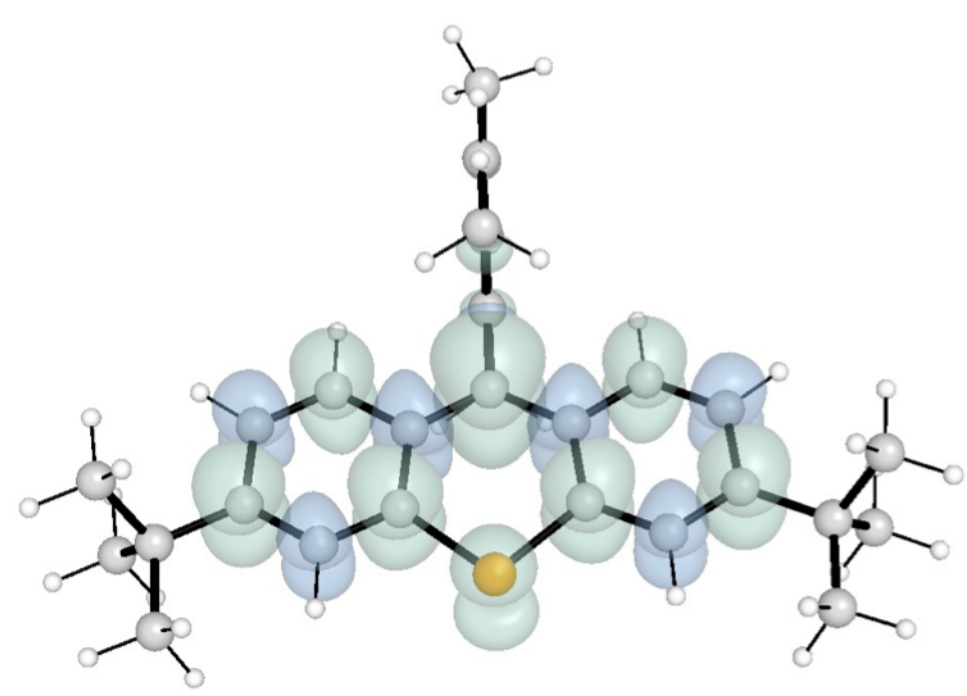

TD-DFT benchmarking

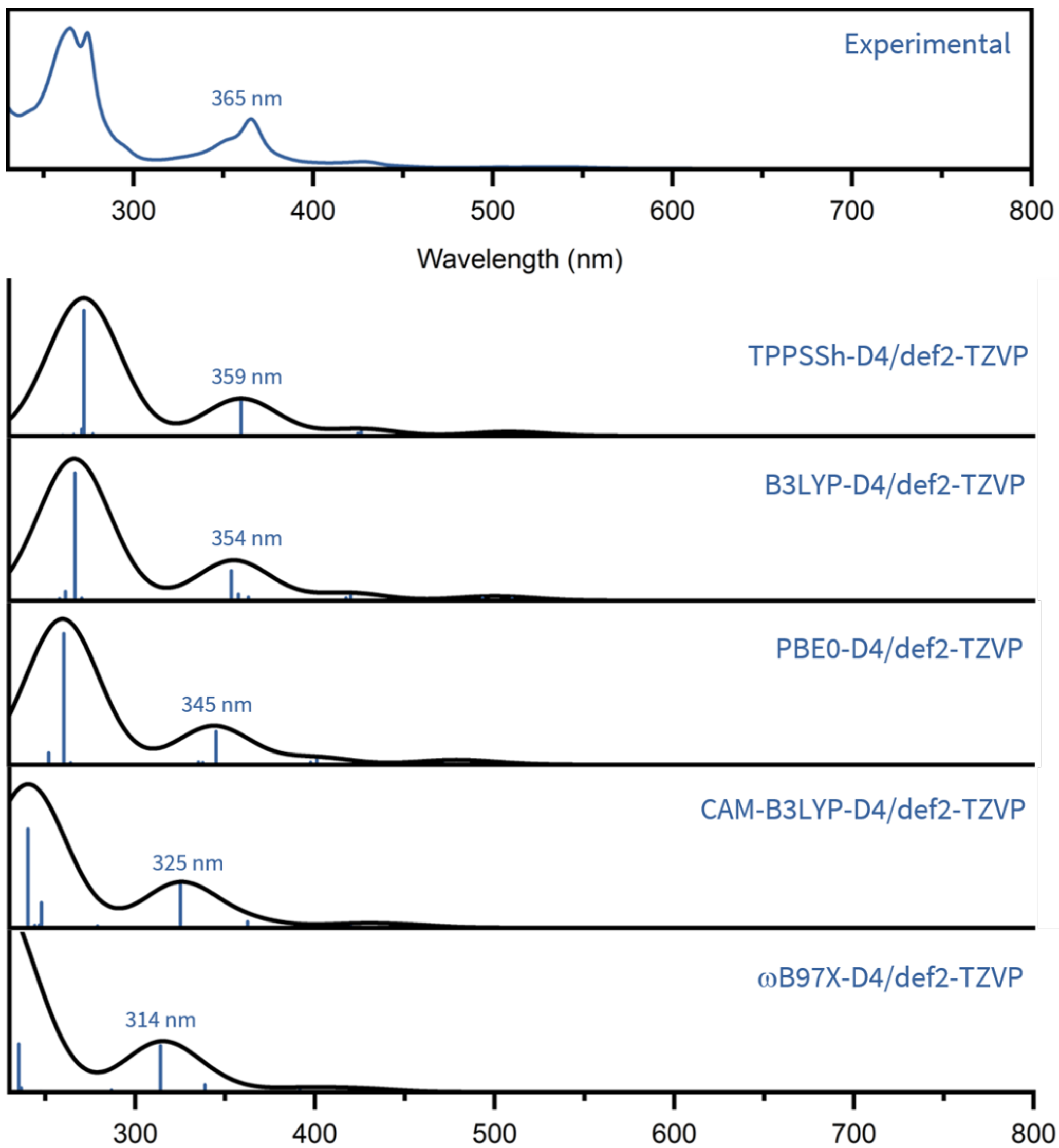

Figure S12. Benchmark of TD-DFT functionals for reproducing the experimental UV–Vis absorption spectrum of **2** in CH_3CN. Calculated spectra were obtained at the TPSSh-D4/def2-TZVP, B3LYP-D4/def2-TZVP, PBE0-D4/def2-TZVP, CAM-B3LYP-D4/def2-TZVP, and ωB97X-D4/def2-TZVP levels of theory. The TD-DFT calculations included 30 excited-state roots. Simulated absorption spectra were constructed by Gaussian broadening of the calculated vertical excitations (σ = 15 nm) and normalized to their maximum intensity. Blue sticks represent individual doublet-doublet transitions with heights proportional to their oscillator strengths.

Calculated transitions

State	***Energy (eV)***	***λ (nm)***	***f_{osc}***	***Dominant orbital transition***
D_1	*2.402*	*516*	*0.018*	*SOMO → LUMO (94.17%)*
D_2	*2.464*	*503*	*0.026*	*HOMO → LUMO (84.13%)*
D_3	*2.910*	*426*	*0.042*	*SOMO → LUMO+1 (83.02%)*
D_4	*2.923*	*424*	*0.030*	*SOMO → LUMO+2 (82.85%)*
D_5	*3.047*	*407*	*0.000*	*HOMO–1 → LUMO (98.77%)*
D_6	*3.087*	*402*	*0.000*	*SOMO → LUMO+3 (98.57%)*
D_7	*3.265*	*380*	*0.001*	*HOMO–2 → LUMO (90.23%)*
D_8	*3.285*	*378*	*0.005*	*SOMO → LUMO+4 (79.48%)*
D_9	*3.453*	*359*	*0.366*	*HOMO –3 → LUMO (69.71%)*

Table S1. TD-DFT results for **2** at the TPSSh-D4/def2-TZVP level of theory. Reported values include vertical excitation energies, absorption wavelengths, oscillator strengths (f_{osc}), and the dominant one-electron orbital transitions with their corresponding weights.

Coordinates for selenoxanthylyl radical 2

Se	12.14136249150700	6.12925589714894	16.14623251875816
C	8.59852146487430	5.80876323969633	19.35333321078053
C	8.72072121460366	6.45739796147048	20.59661591160815
C	7.68036665828727	6.35430672221014	21.52626464765654
H	7.78261008478829	6.85902555569064	22.49358624973438
C	6.51959784833637	5.62449989351869	21.25393456697716
C	6.41966542331070	4.98515031459481	20.01421713896706
H	5.52108097283360	4.40257204064506	19.78150752779974
C	7.43863743148887	5.06595564723245	19.05978977757655
C	9.70311447330003	5.90469011014410	18.34597295866049
C	9.65413786648570	6.99280853513366	17.39570225304379
C	10.61979873459787	7.22377316008158	16.38581952454051
C	11.85337419574999	4.85407214447861	17.51140057059174
C	10.74200948959437	4.90059200828252	18.38829621689688
C	9.95890496842430	7.25047088555512	20.92673201683211
H	10.85969061075006	6.61570870070772	20.89806527731152
H	9.88885245045389	7.69682122333724	21.92939164530830
H	10.11817051348386	8.06456615583354	20.20093500359071
C	5.39610496861560	5.55065520968510	22.25473484437794
H	4.65233742345519	6.34383757242799	22.06947617506457
H	5.76464094770785	5.68061042413498	23.28335274104186
H	4.86690512036947	4.58756179163389	22.19454359356298
C	7.29676080582762	4.36263614250109	17.73459376452380
H	7.35419890050498	5.07539027220330	16.89574728275361
H	6.33504580488690	3.83347052028958	17.66674741801675

H	8.10299087623945	3.62609296162905	17.58418378265987
C	8.57857965504101	7.92440894380580	17.43516609573880
H	7.80346508560369	7.79696638954486	18.19436483428118
C	8.47830774809981	8.98184516947621	16.55171362294358
H	7.62359758430653	9.65892981272054	16.64214933662637
C	9.44536389786407	9.20008106550956	15.54913578376981
C	10.50786053305989	8.29789194478177	15.49364801530269
H	11.28852023588118	8.41794446881262	14.73735335250839
C	12.82209928333717	3.84652081960709	17.60671628477196
H	13.65393700218344	3.86903072975557	16.89716201349017
C	12.75025958892361	2.83659776736363	18.56625233523286
C	11.64904287176063	2.87320569784015	19.44595703523642
H	11.54164176134491	2.10572076128519	20.21837933384323
C	10.68790331826324	3.86201638199675	19.36019498744893
H	9.85249459489838	3.84734158379020	20.06395061731155
C	9.30209050230436	10.38398230058101	14.58644860035521
C	9.29304729129507	11.69173228703568	15.39497860221501
H	10.23213558515314	11.81623396571353	15.95881966816130
H	8.45871100407516	11.72273747472777	16.11342854989209
H	9.18499979925561	12.55698252828973	14.72015909367314
C	13.79652875101535	1.72319406178380	18.68772556144949
C	14.91188121267016	1.86507701270959	17.64791154845767
H	15.63887597782733	1.04664005239338	17.77142526687931
H	15.45896013232877	2.81509615413571	17.75864066114547
H	14.52343153791249	1.81150591998185	16.61823889336845
C	7.97690730481198	10.25127391129531	13.81821834602760

H	7.85269402497101	11.09746612002638	13.12228276980988
H	7.10931031312779	10.24725903709407	14.49676992144460
H	7.95138077590491	9.32026340105651	13.22837763604231
C	10.44919000968636	10.44432459466694	13.57369491105796
H	10.49109643551507	9.53992952829900	12.94569433374719
H	11.42707202371488	10.56467947994361	14.06686842257224
H	10.30657763229330	11.30743724627209	12.90406435845536
C	13.10882247475916	0.36297591762160	18.48545085645181
H	12.32169604595471	0.18687576621631	19.23562829169606
H	13.84430936279464	-0.45364087398839	18.57438268265740
H	12.64689866493454	0.29647355966595	17.48668006303485
C	14.42555476797097	1.77268001108271	20.08978000608751
H	14.92715117338994	2.73847505959759	20.26573102940354
H	15.17849684372496	0.97458439098494	20.19858335505820
H	13.67317701903813	1.63167433430600	20.88177425809670

NMR Spectroscopic Data

*^{1}H NMR of 1-tosyl-1*H*-benzo[*d*]imidazole (**S1**) – $CDCl_3$, 23 ºC*

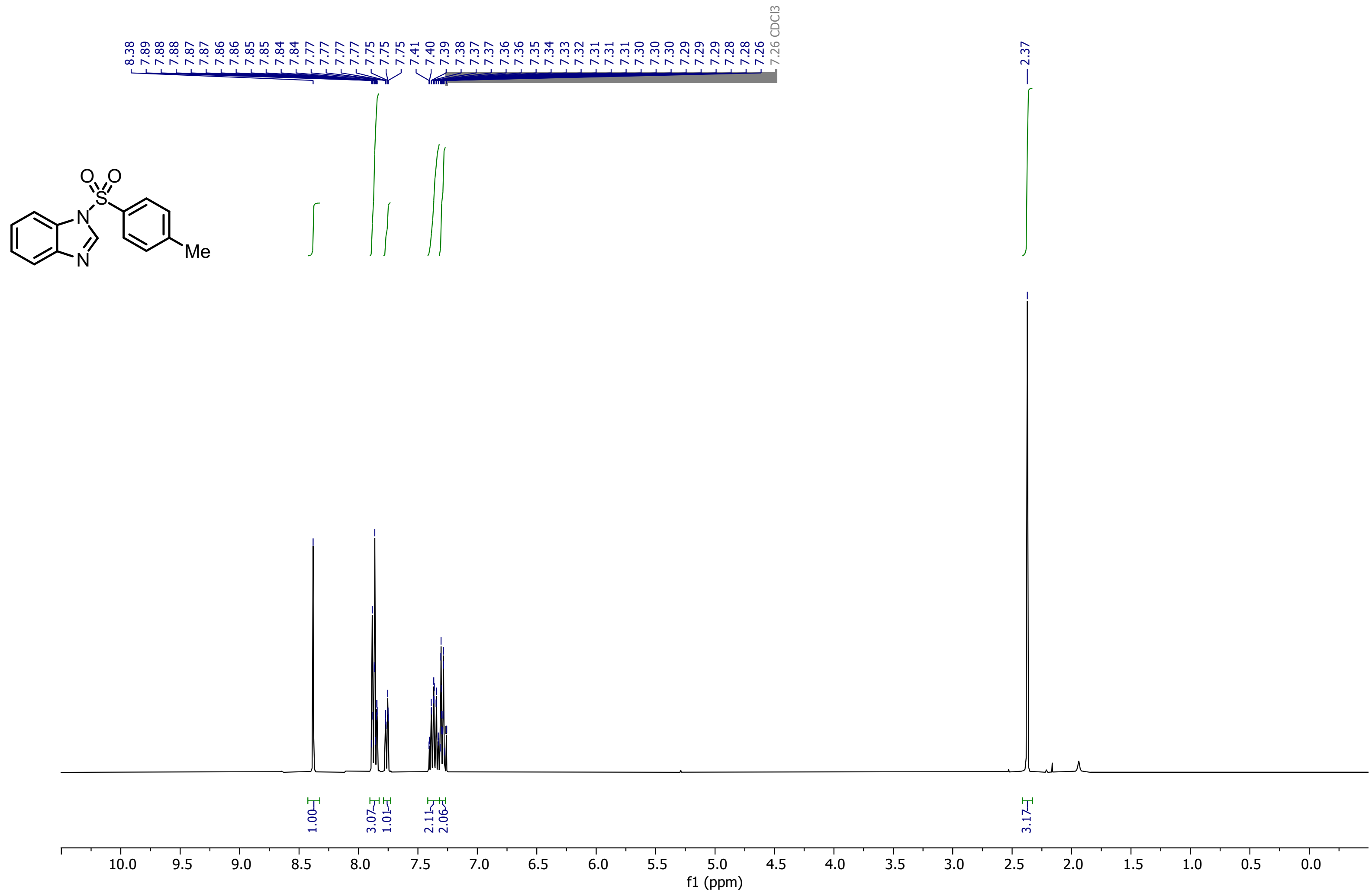

*^{13}C NMR of 1-tosyl-1*H*-benzo[*d*]imidazole (**S1**) – $CDCl_3$, 23 ºC*

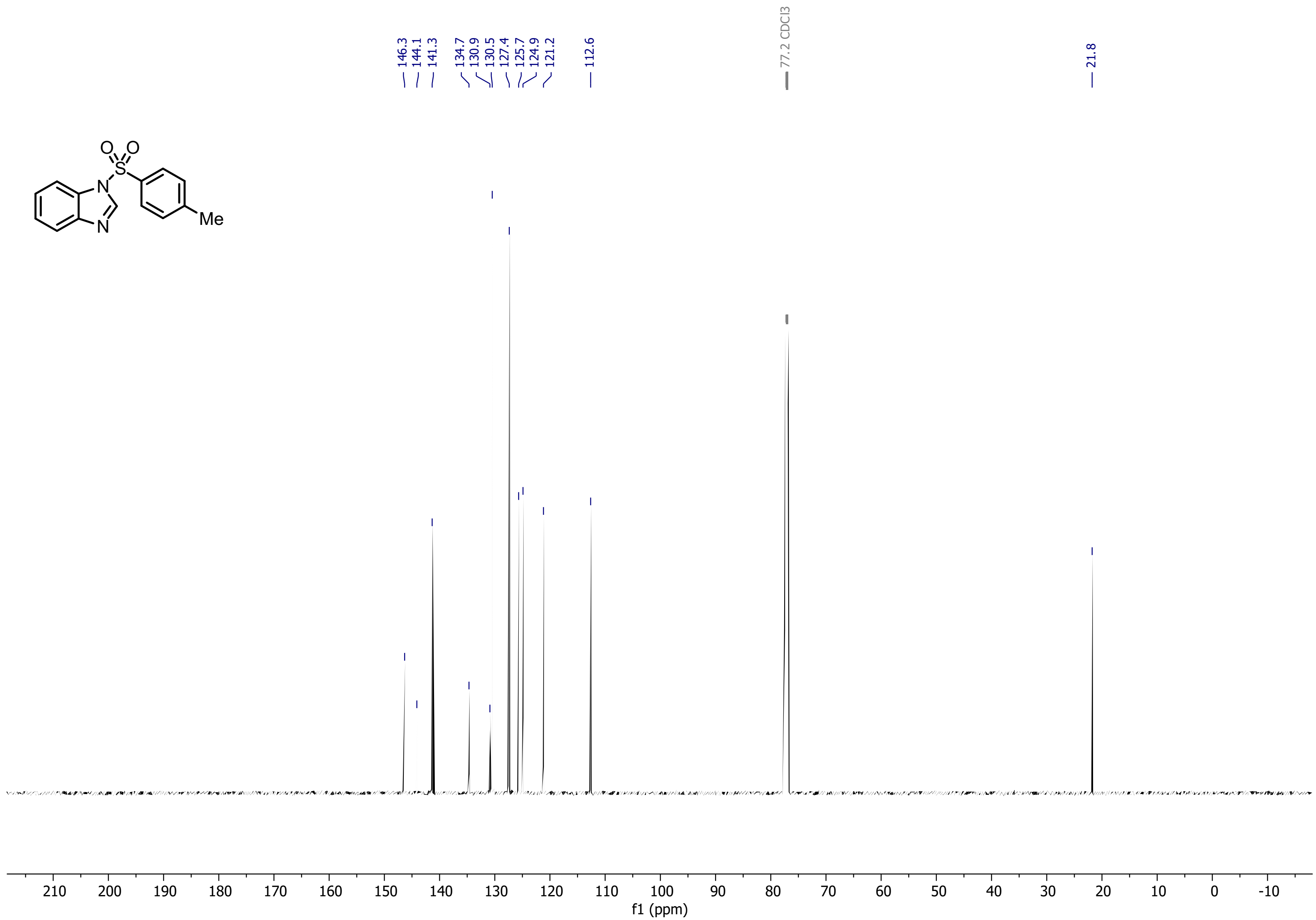

*^{1}H NMR of 1-tosyl-1*H*-indole (**S2**) – CDCl$_3$, 23 ºC*

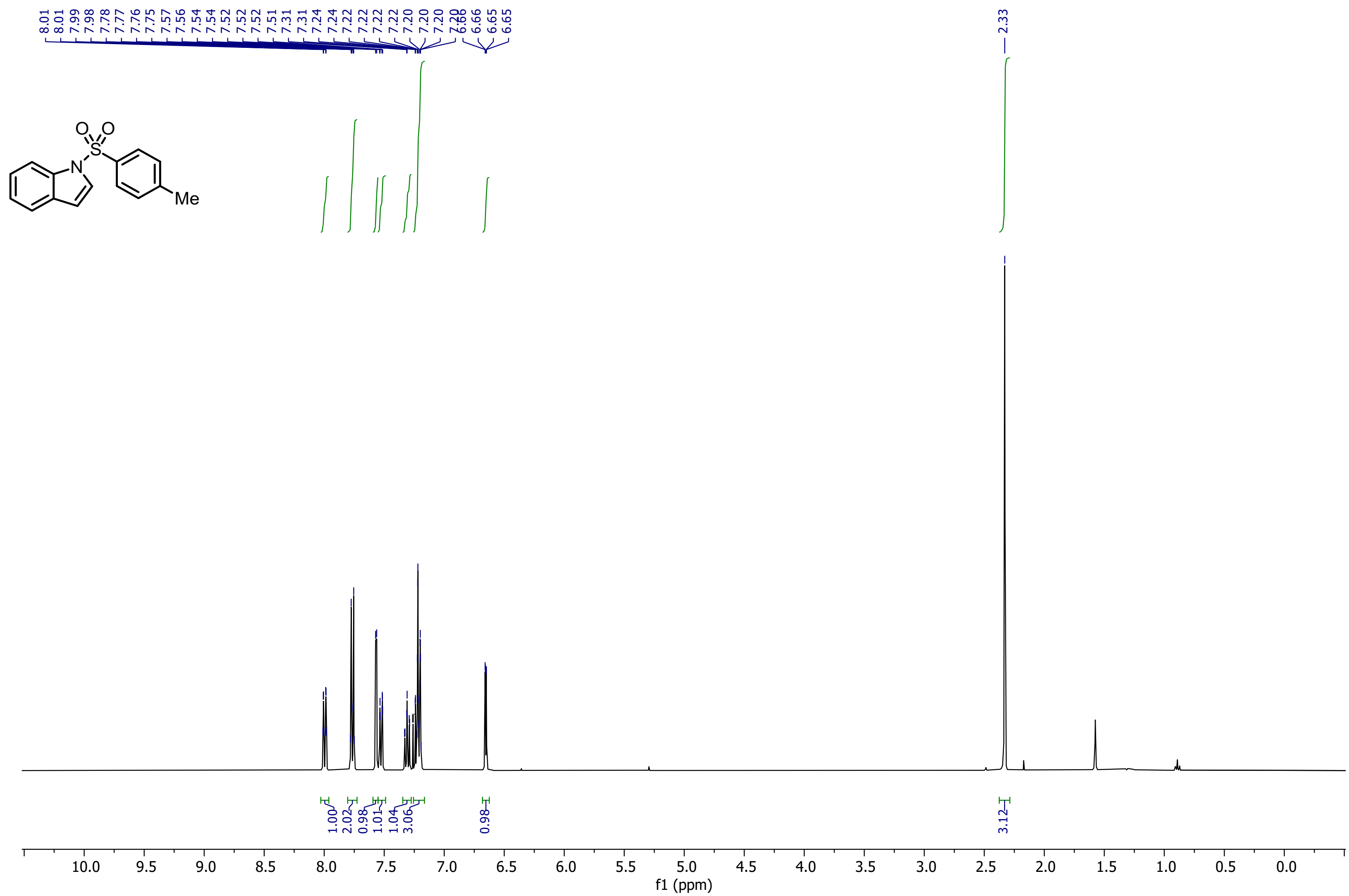

^{13}*C NMR of 1-tosyl-1*H*-indole (**S2**) – CDCl$_3$, 23 ºC*

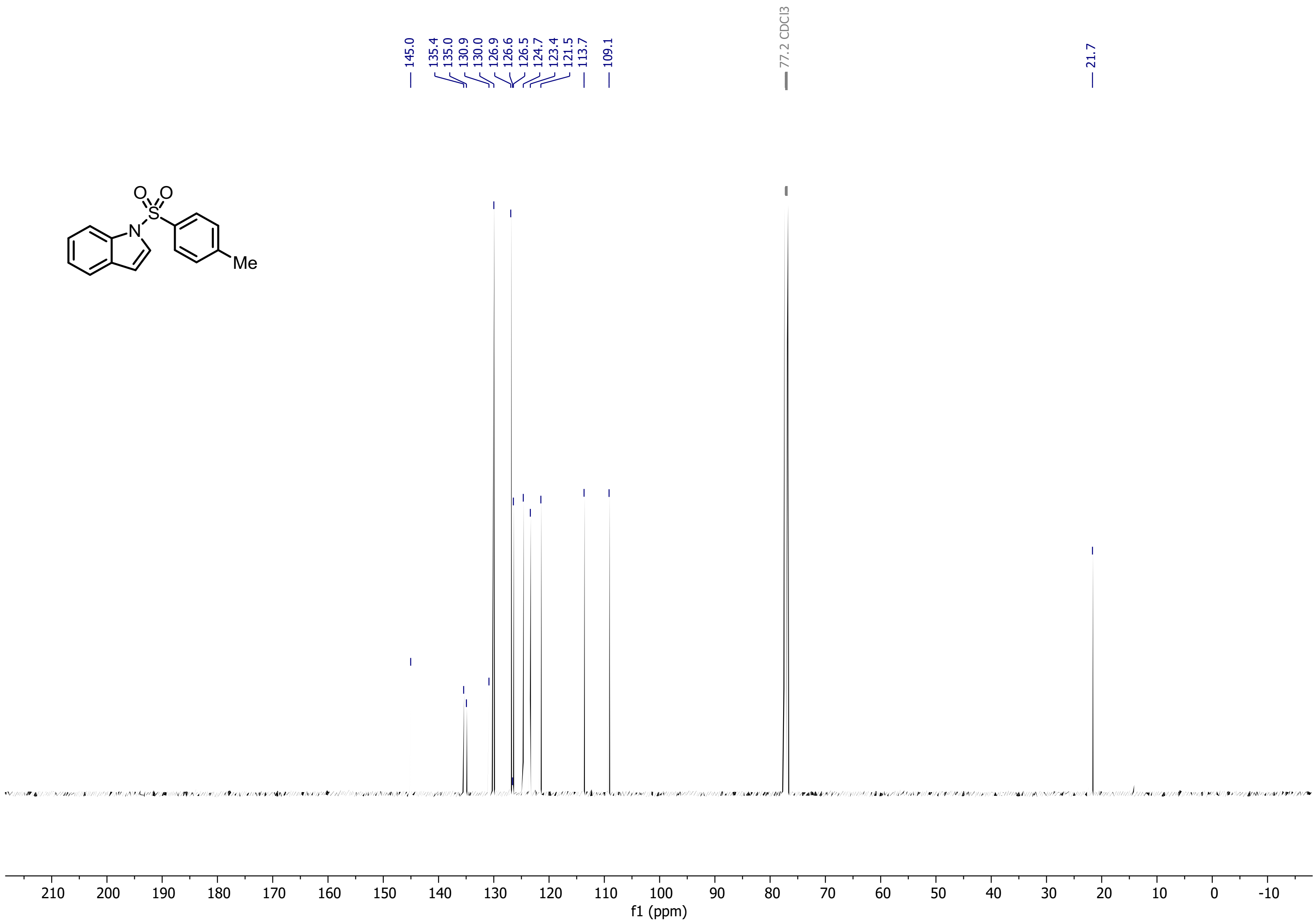

*^{1}H NMR of 1-tosyl-1*H*-indole-3-carbonitrile (**S3**) – $CDCl_3$, 23 ºC*

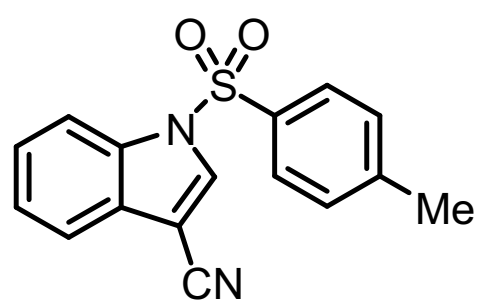

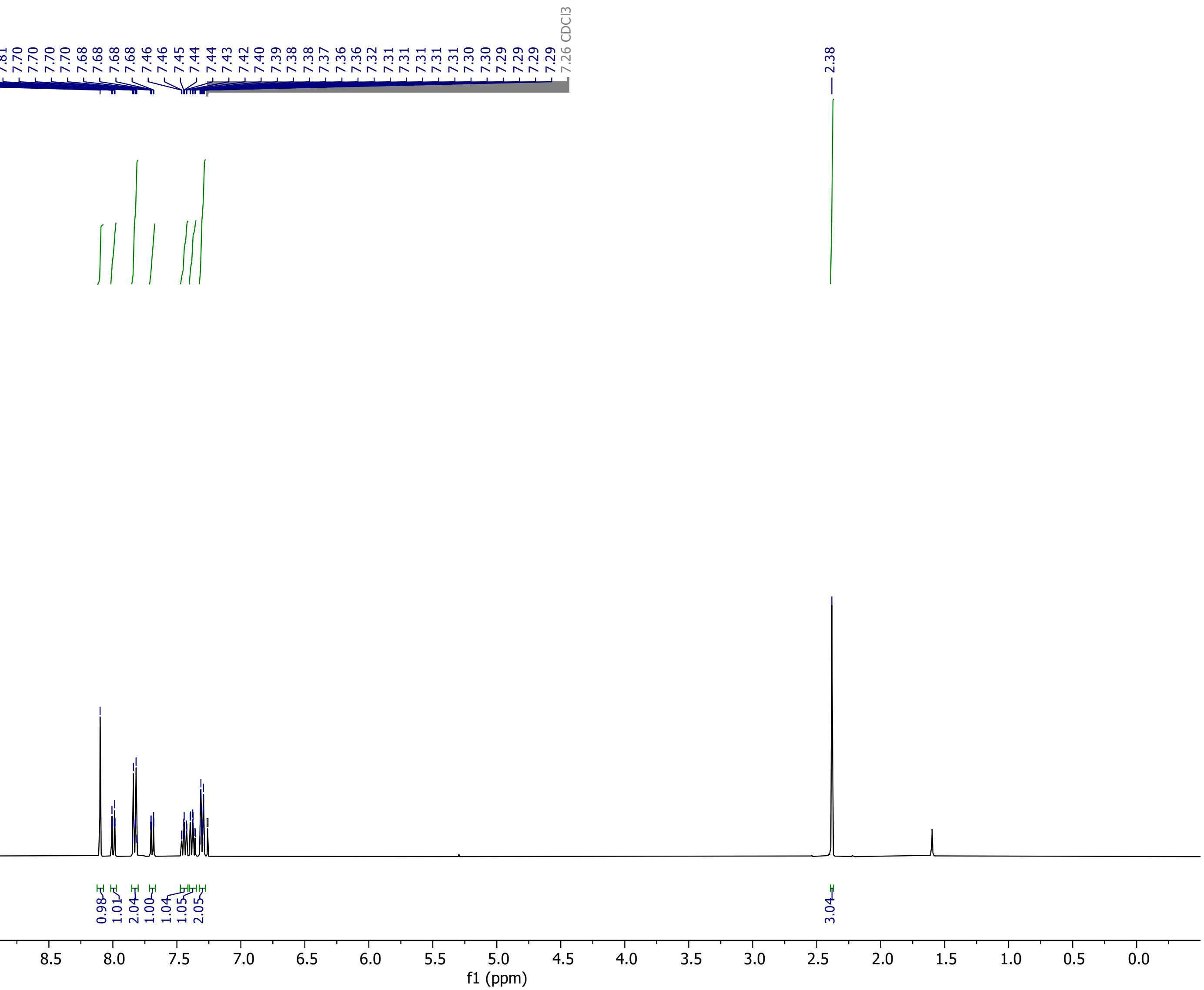

^{13}C NMR of 1-tosyl-1H-indole-3-carbonitrile (**S3**) – $CDCl_3$, 23 ºC

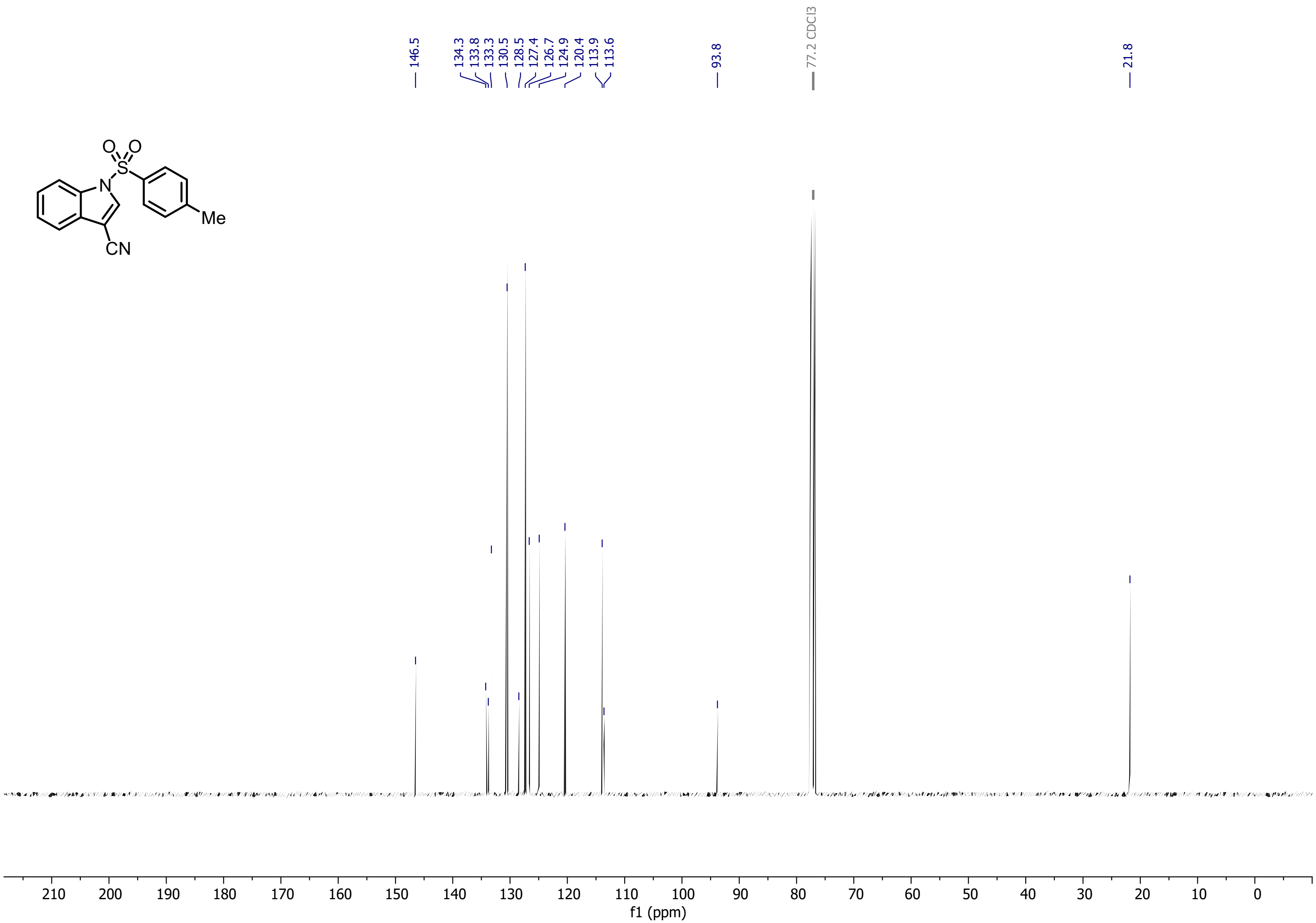

[1] *H NMR of 1-tosyl-1*H*-indazole (**S4**) – CDCl$_3$, 23 ºC*

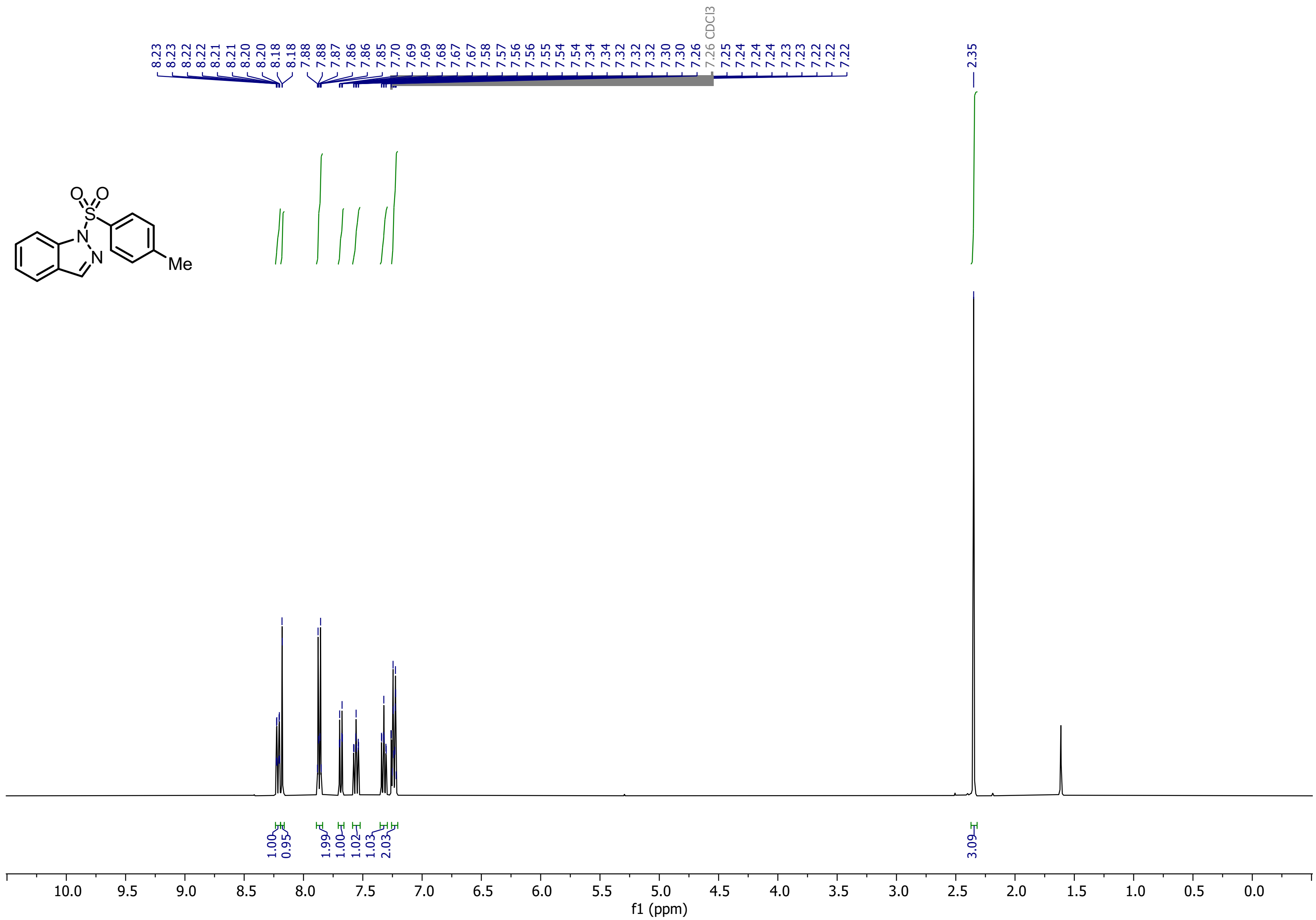

*^{13}C NMR of 1-tosyl-1*H*-indazole (**S4**) – $CDCl_3$, 23 ºC*

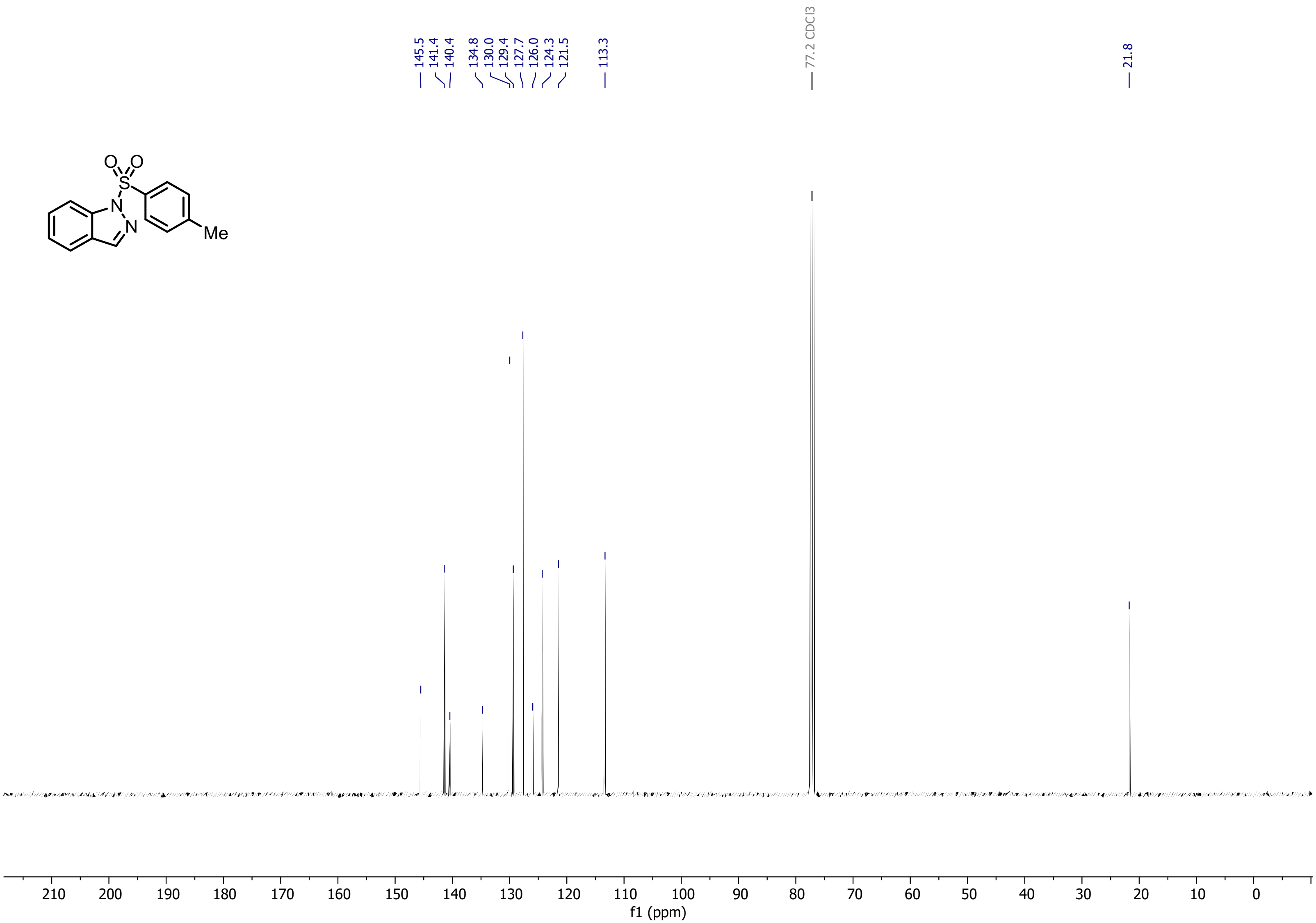

^{1}H NMR of 9-tosyl-9H-carbazole (**S5**) – $CDCl_3$, 23 ºC

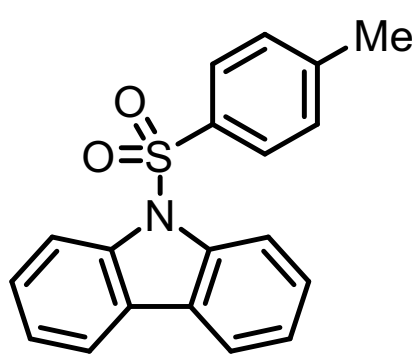

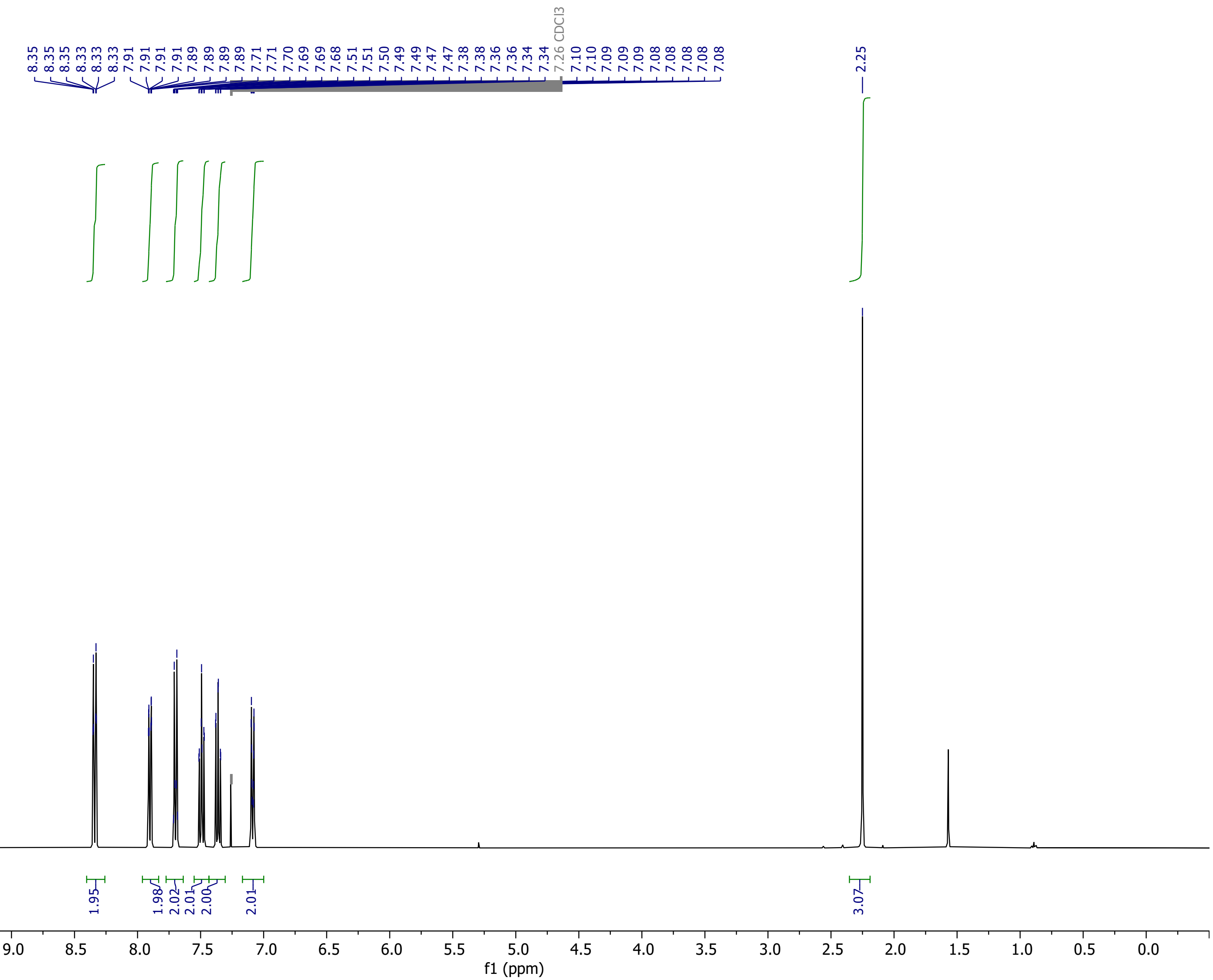

*^{13}C NMR of 9-tosyl-9*H*-carbazole (**S5**) – $CDCl_3$, 23 ºC*

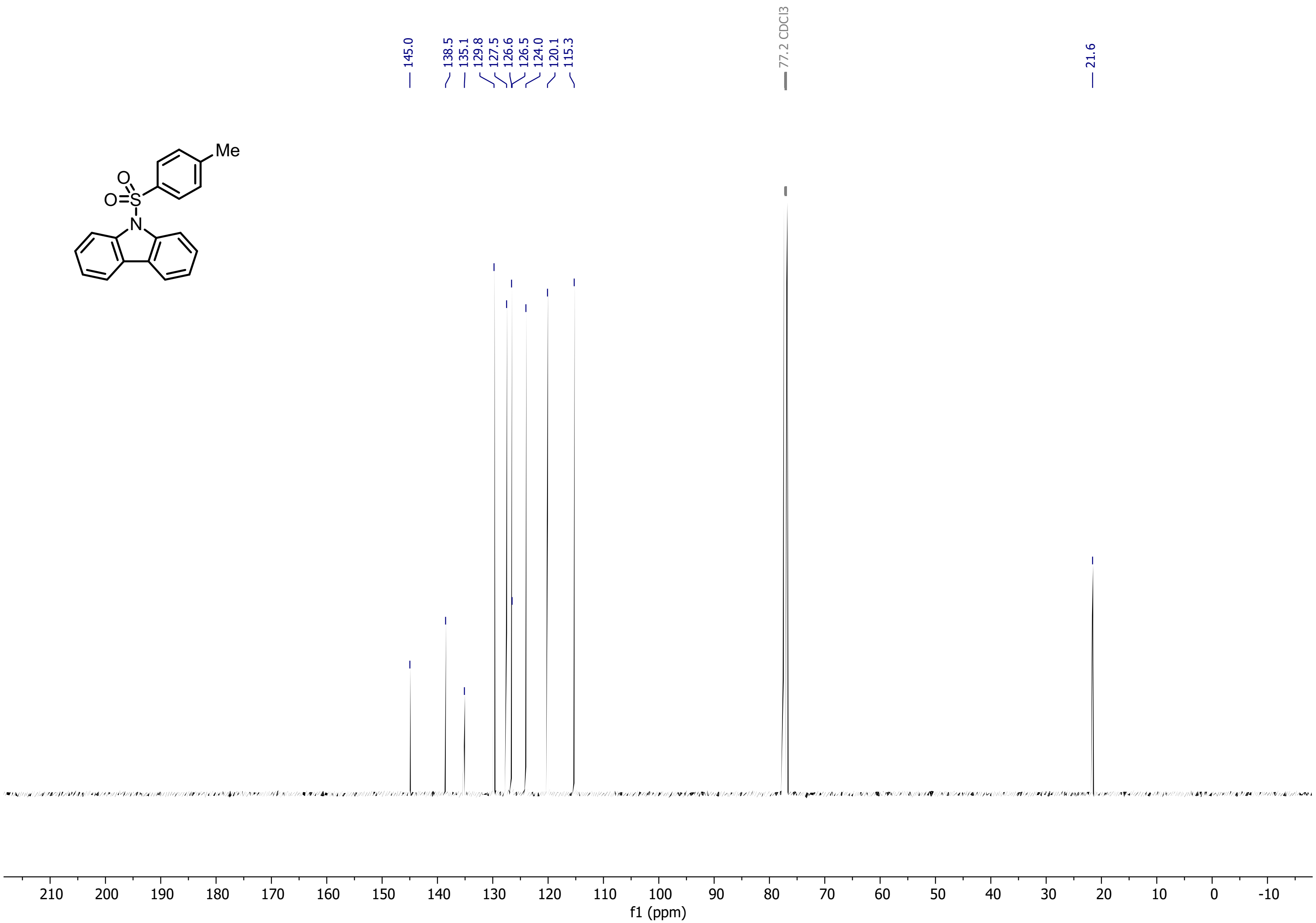

*^{1}H NMR of 4-methyl-*N*-phenyl-*N*-tosylbenzenesulfonamide (**S6**) – $CDCl_3$, 23 ºC*

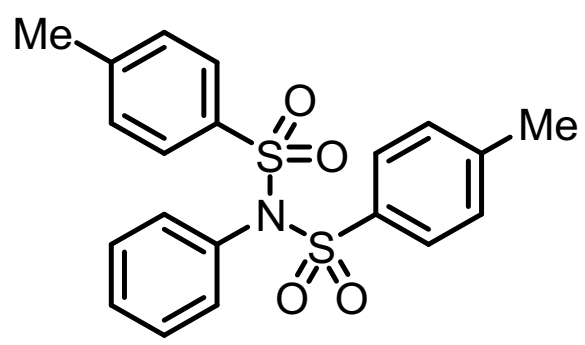

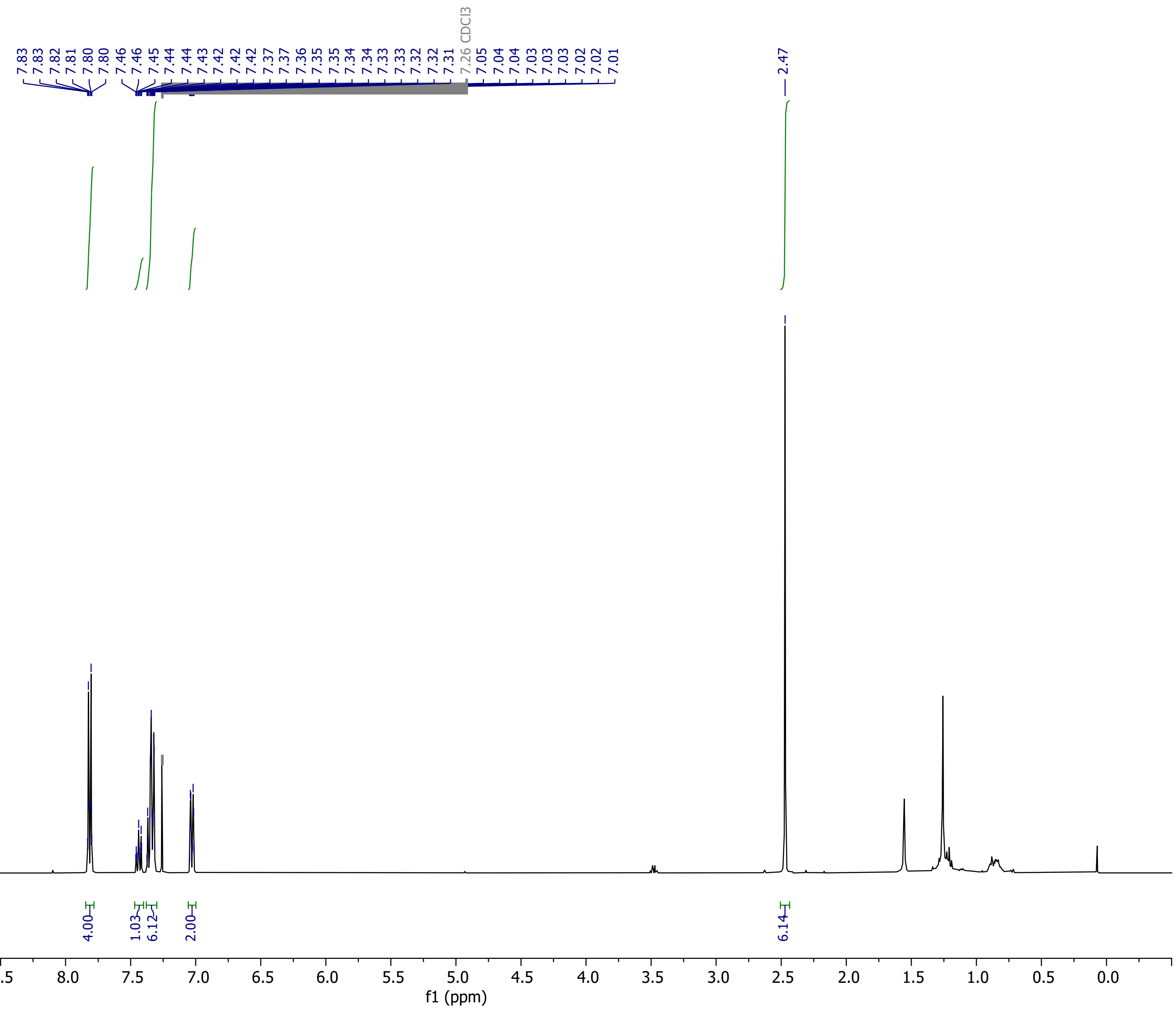

^{13}*C NMR of 4-methyl*-N-*phenyl*-N-*tosylbenzenesulfonamide* (***S6***) – *CDCl*$_3$*, 23 ºC*

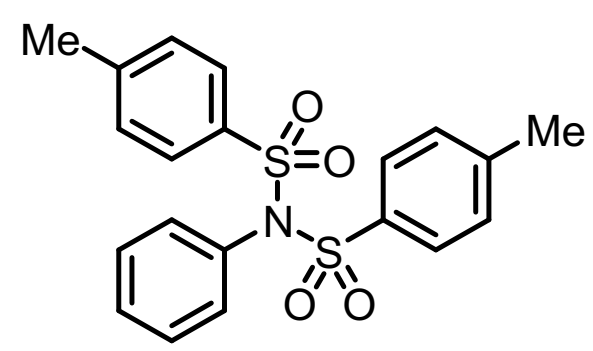

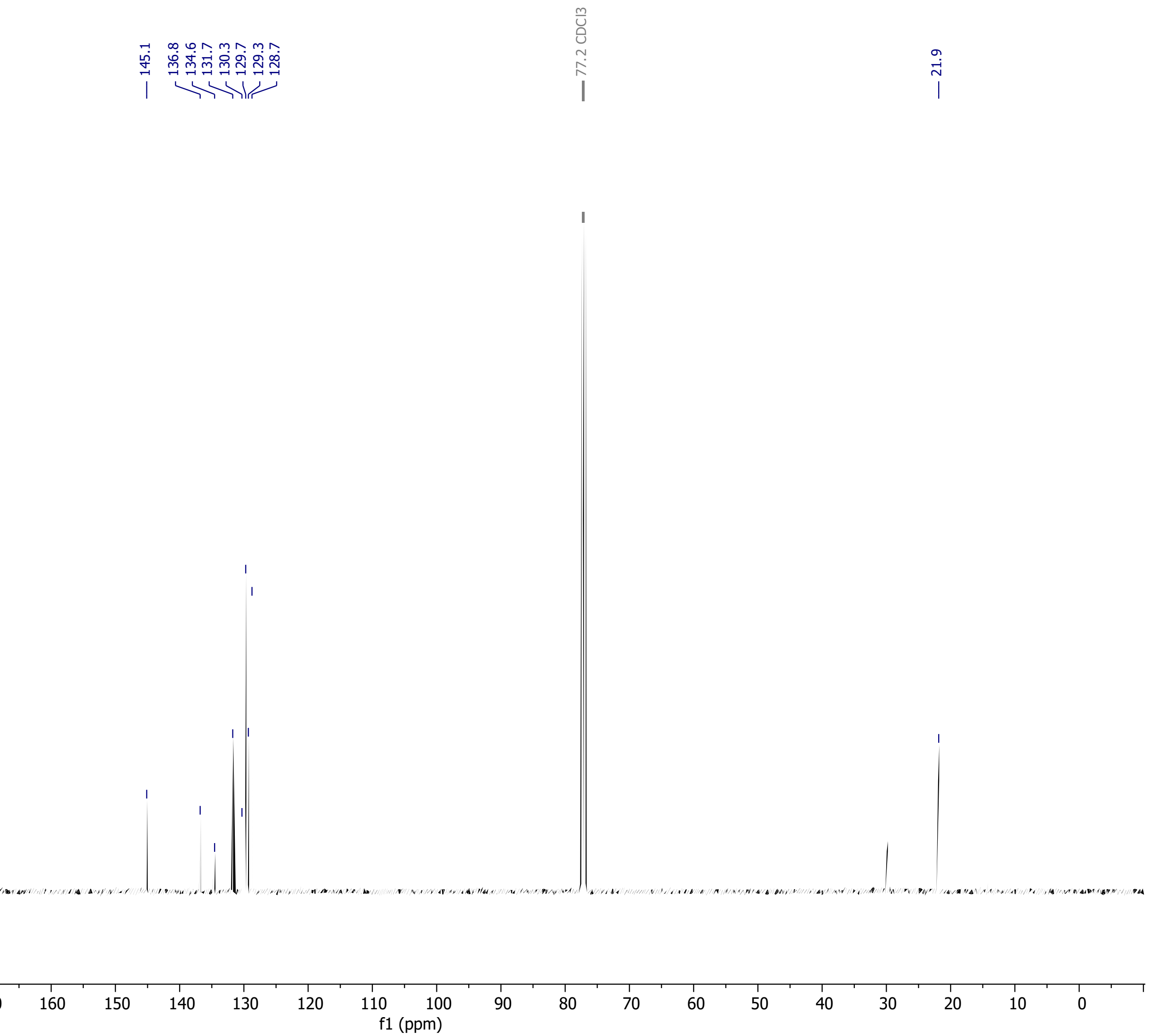

*^{1}H NMR of 4-methyl-*N*-phenylbenzenesulfonamide (**S7**) – $CDCl_3$, 23 ºC*

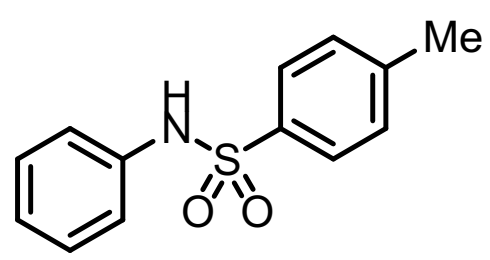

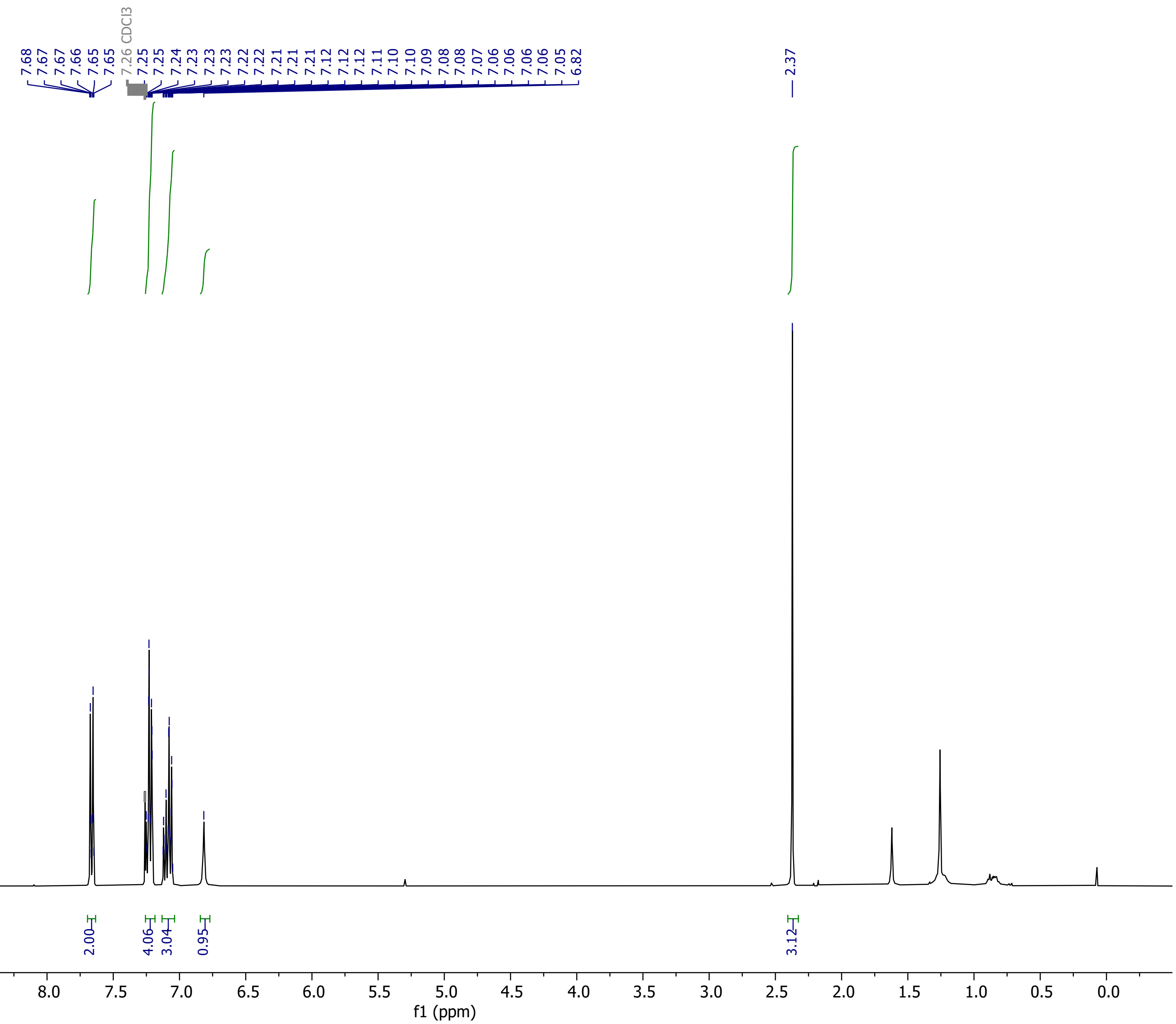

*^{13}C NMR of 4-methyl-*N*-phenylbenzenesulfonamide (**S7**) – $CDCl_3$, 23 ºC*

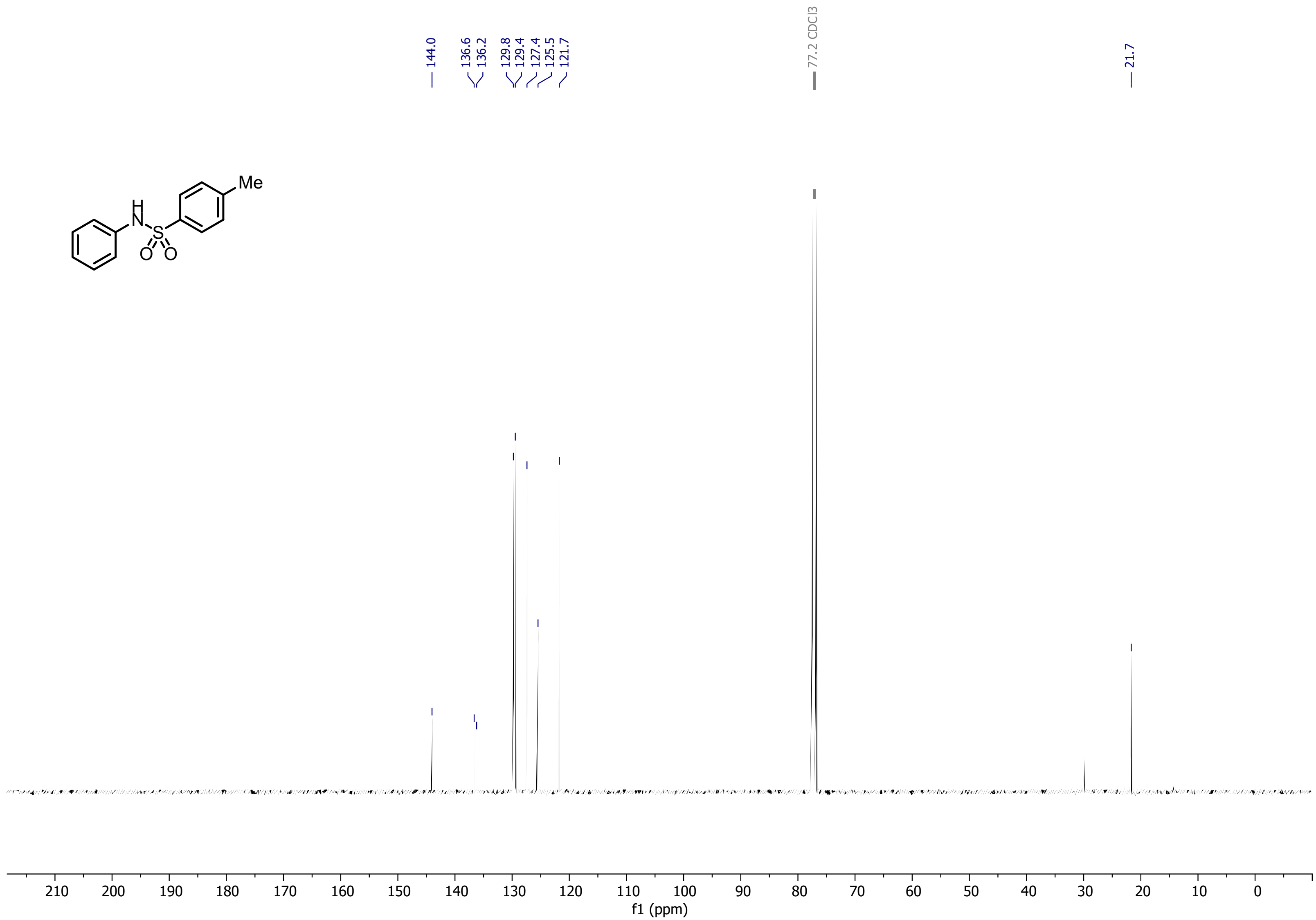

^{1}H NMR of 4-methyl-N-(p-tolyl)benzenesulfonamide (**S8**) – $CDCl_3$, 23 °C

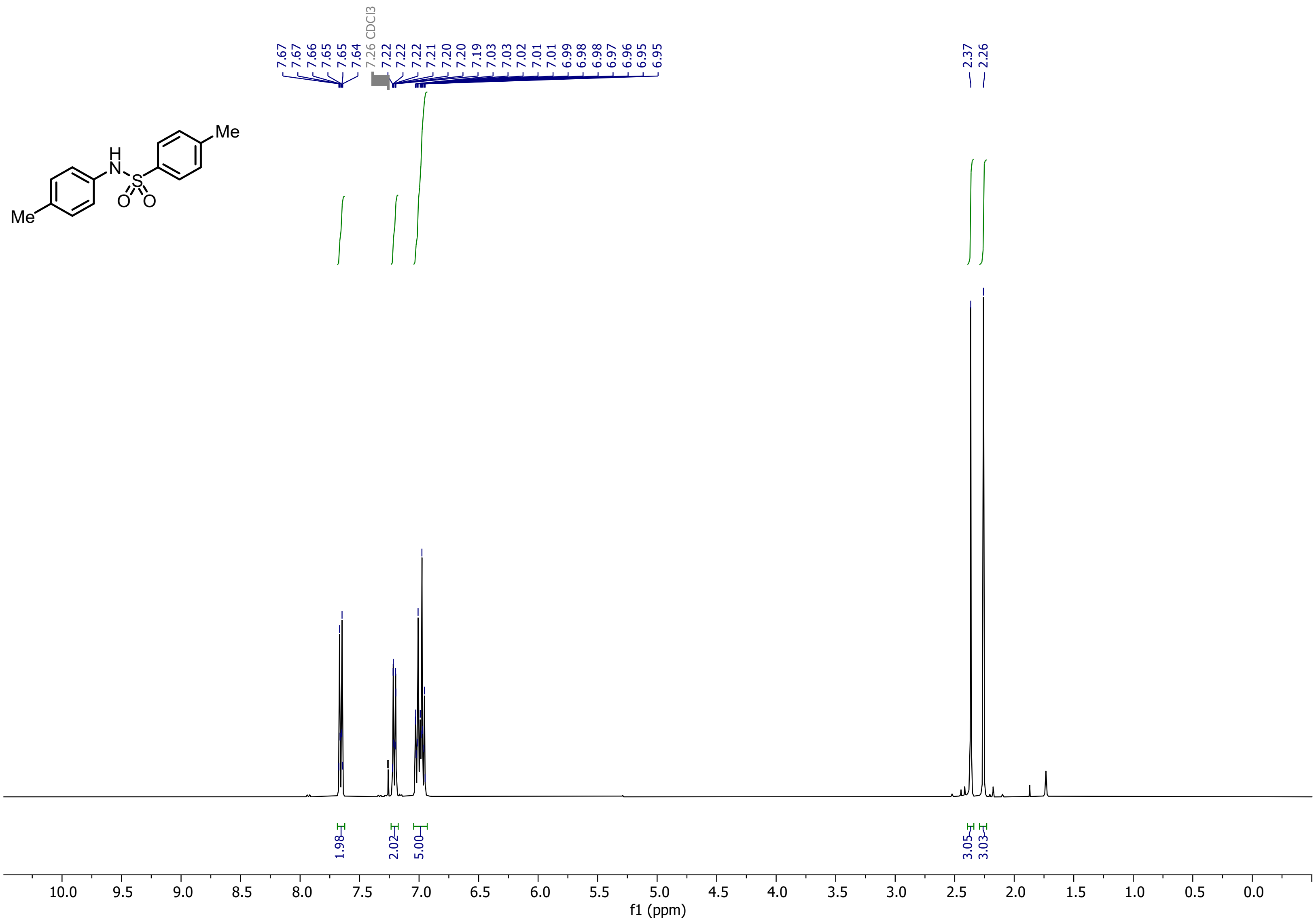

*^{13}C NMR of 4-methyl-*N*-(p-tolyl)benzenesulfonamide (**S8**) – $CDCl_3$, 23 ºC*

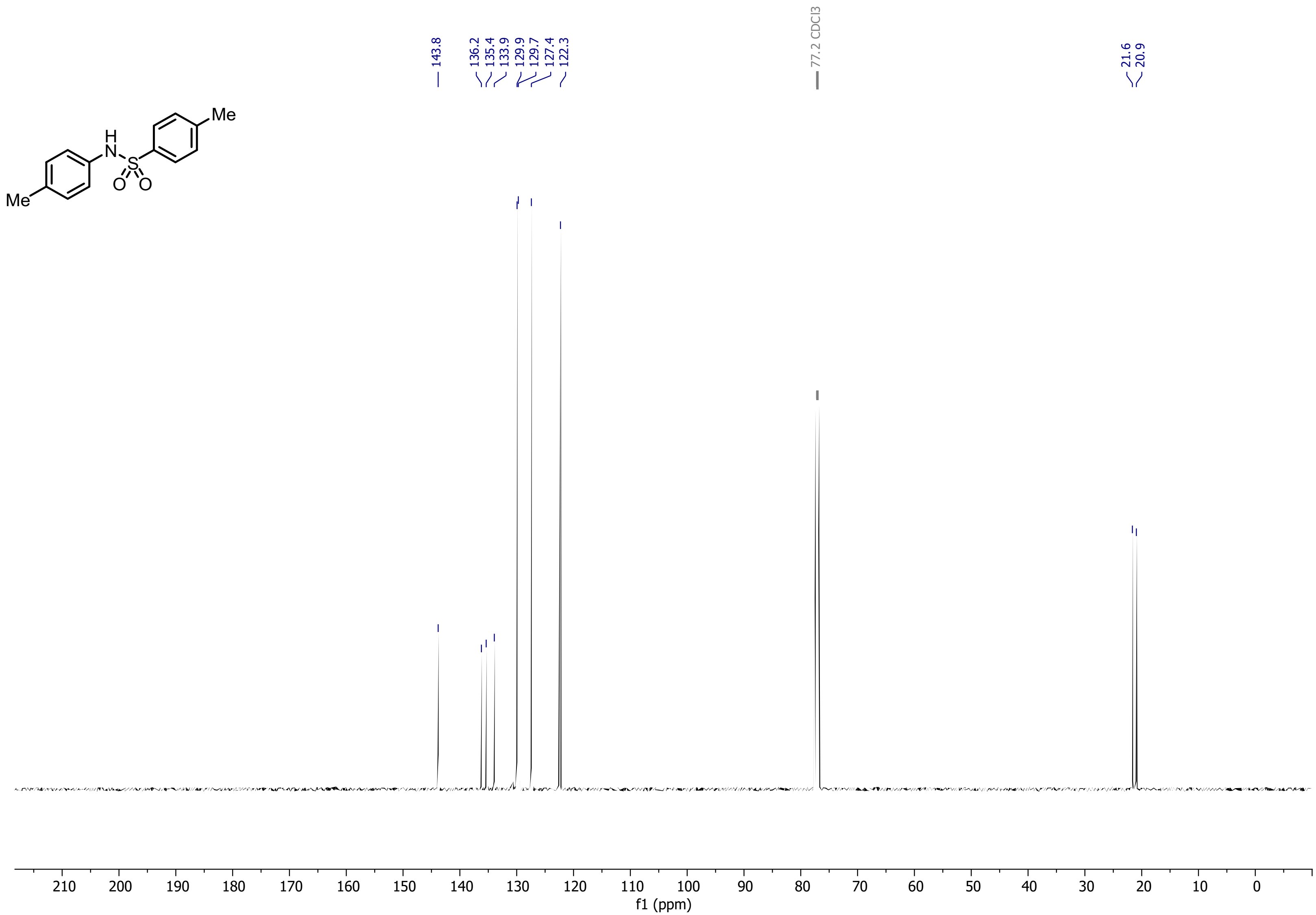

^{1}H NMR of N-*(4-(*tert-*butyl)phenyl)-4-methylbenzenesulfonamide (**S9**) – $CDCl_3$, 23 ºC*

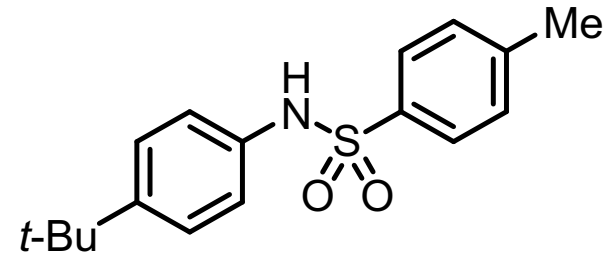

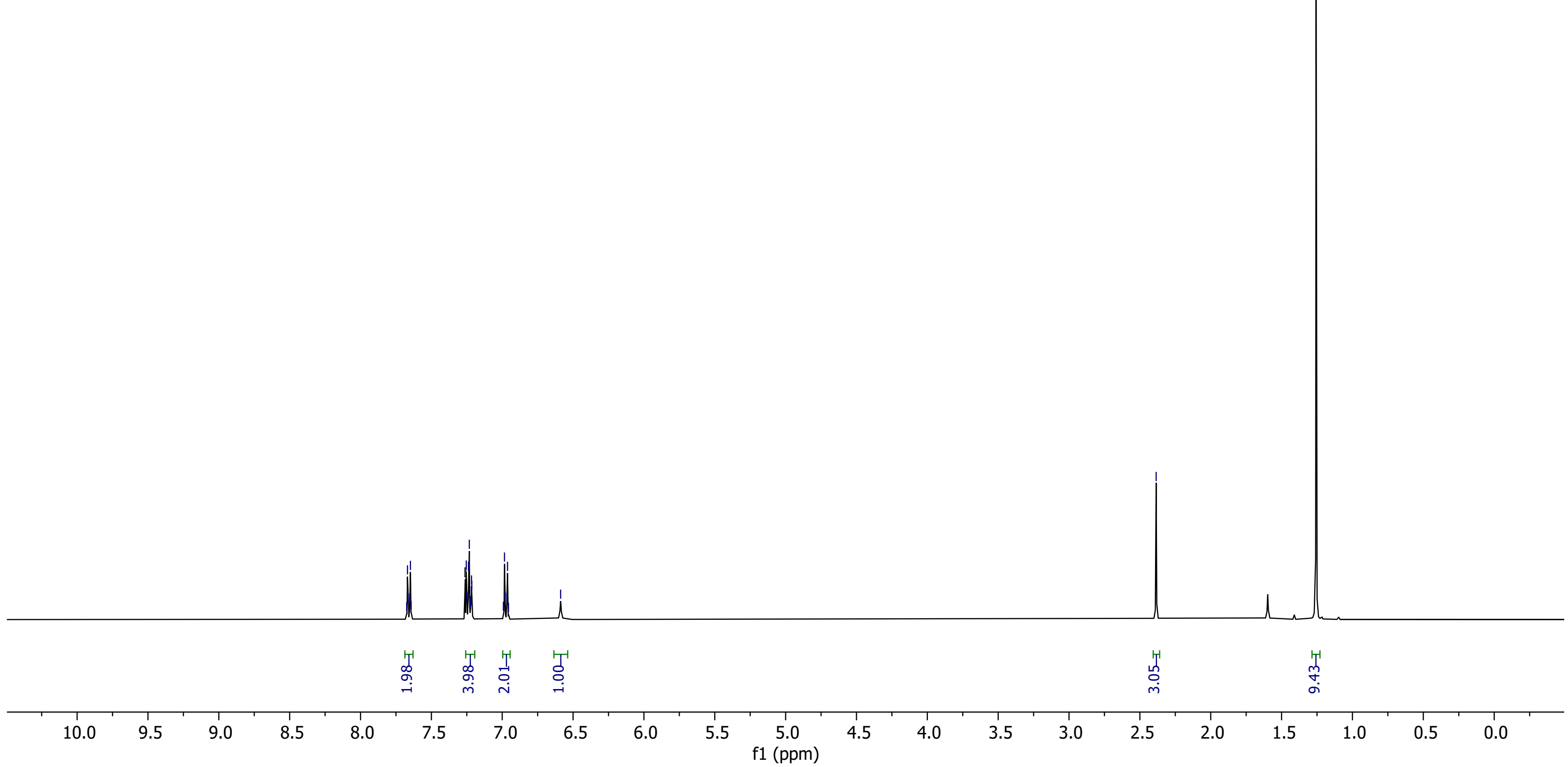

^{13}C NMR of N-(4-(tert-butyl)phenyl)-4-methylbenzenesulfonamide (**S9**) – $CDCl_3$, 23 ºC

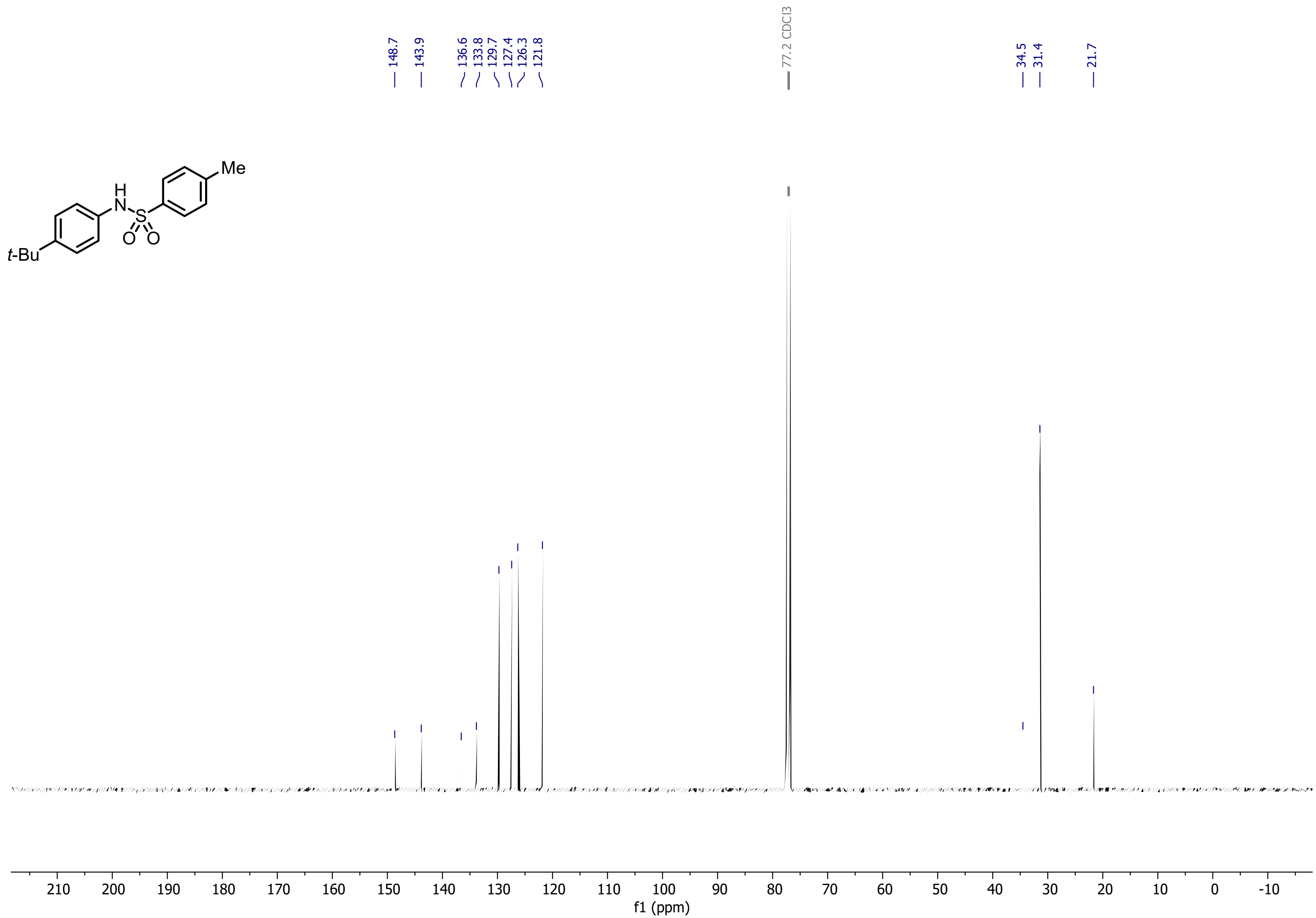

^{1}H NMR of 2-tosyl-1,2,3,4-tetrahydroisoquinoline (***S10***) – $CDCl_3$, 23 °C

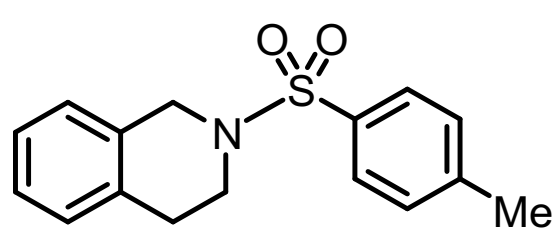

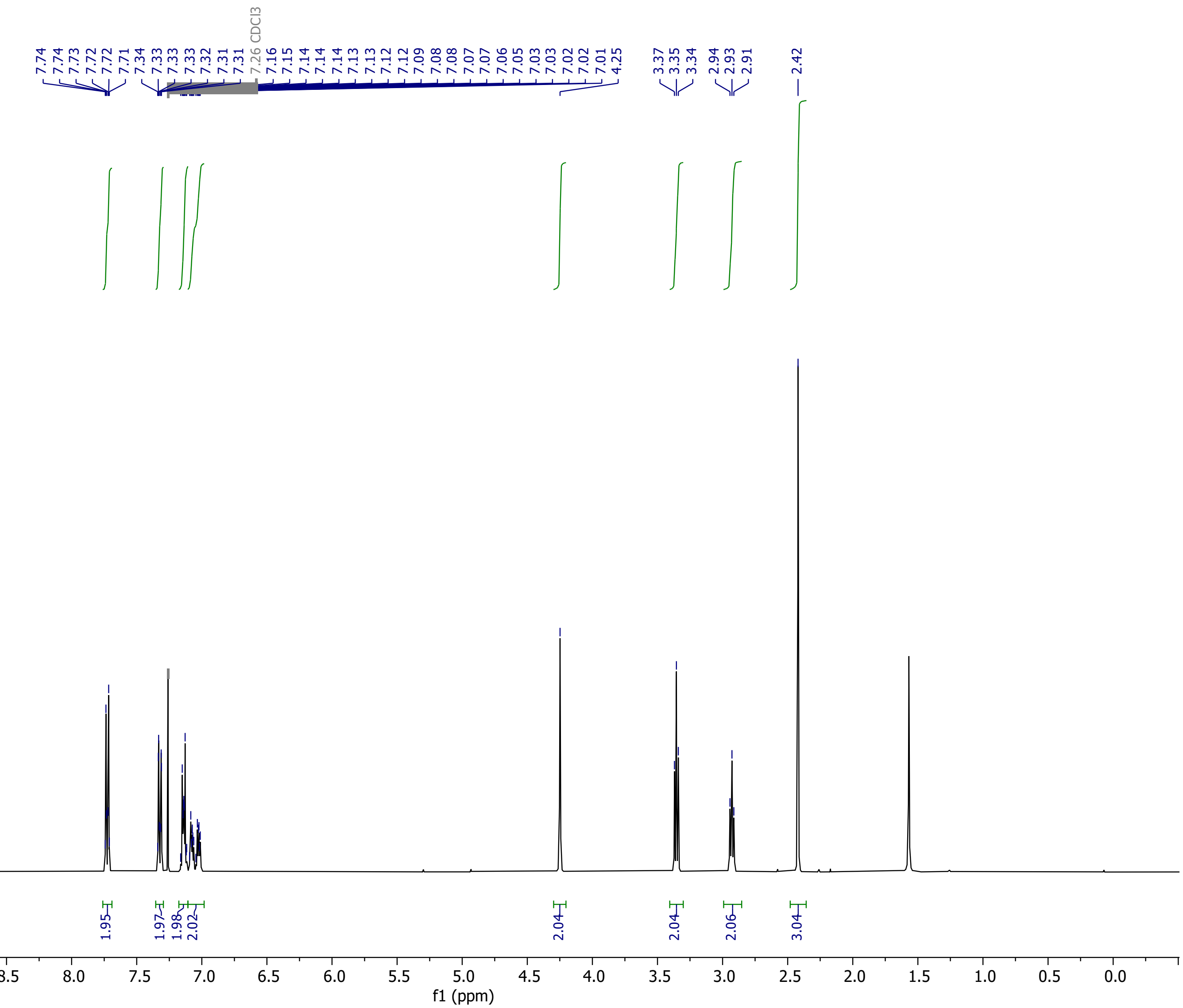

^{13}*C NMR of 2-tosyl-1,2,3,4-tetrahydroisoquinoline (**S10**) – CDCl$_3$, 23 ºC*

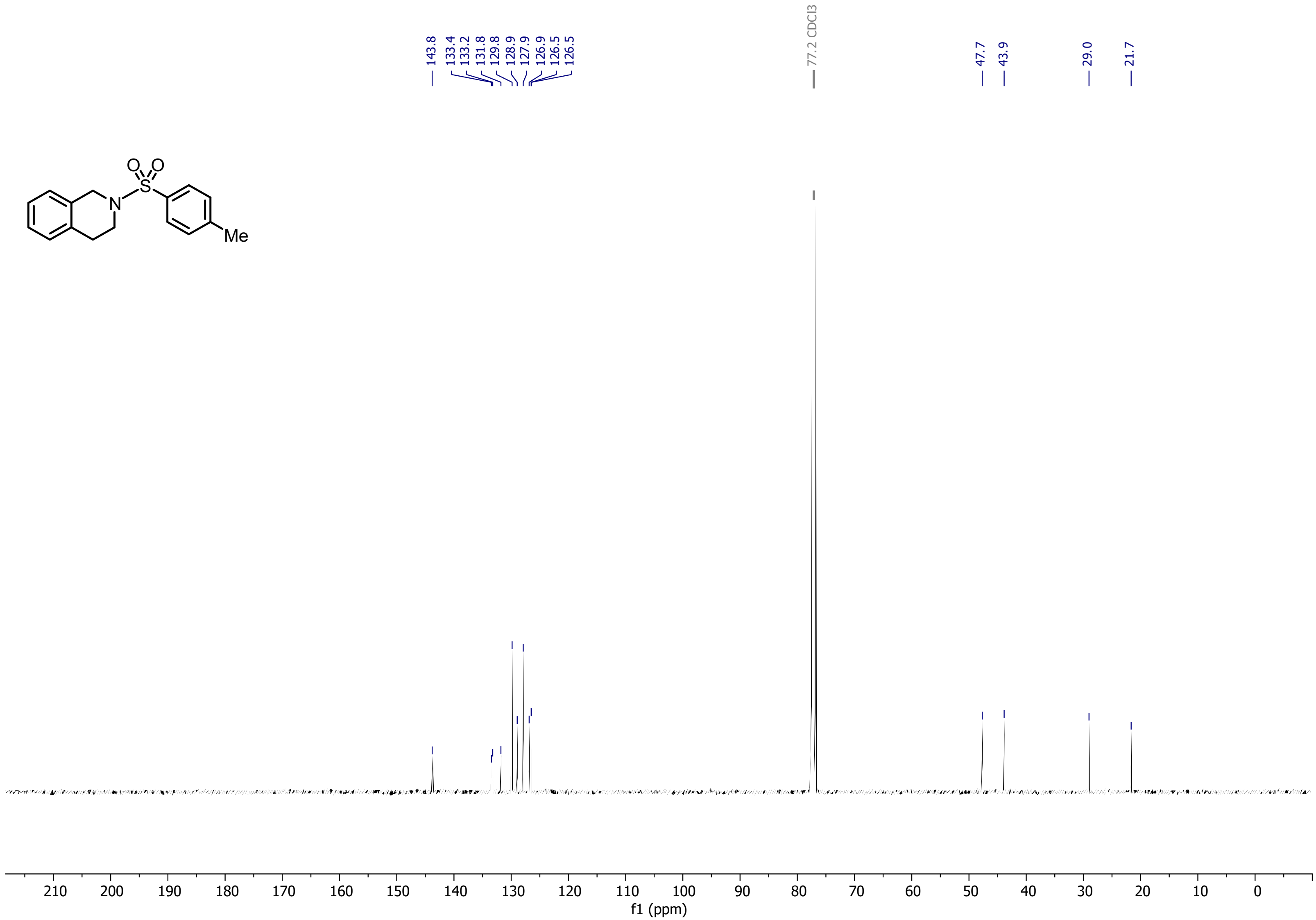

^{1}H NMR of benzimidazole (**3**) – DMSO-d6, 23 ºC

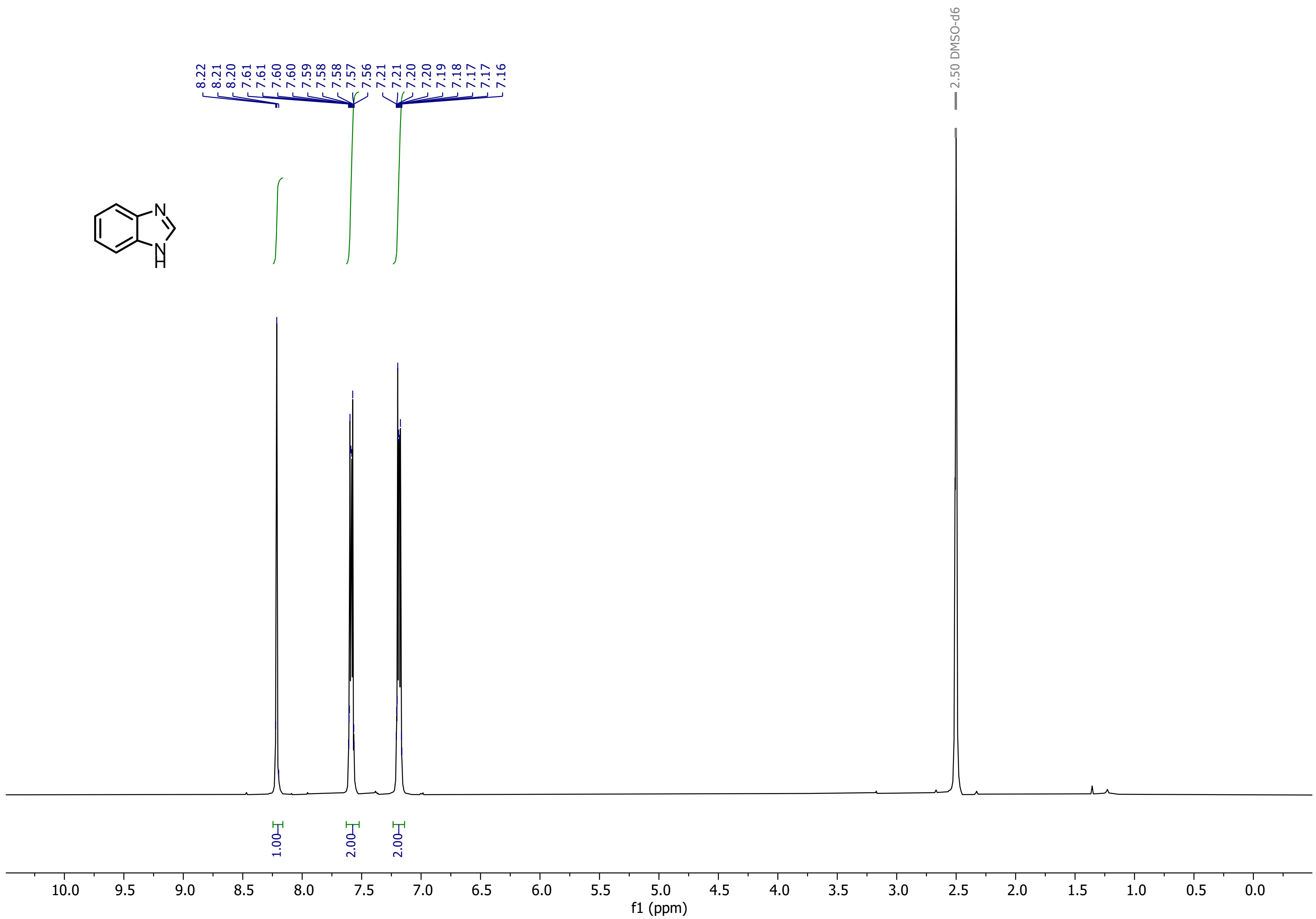

^{13}C NMR of benzimidazole (**3**) – DMSO-d6, 23 ºC

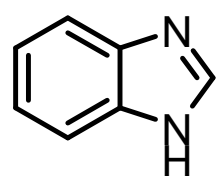

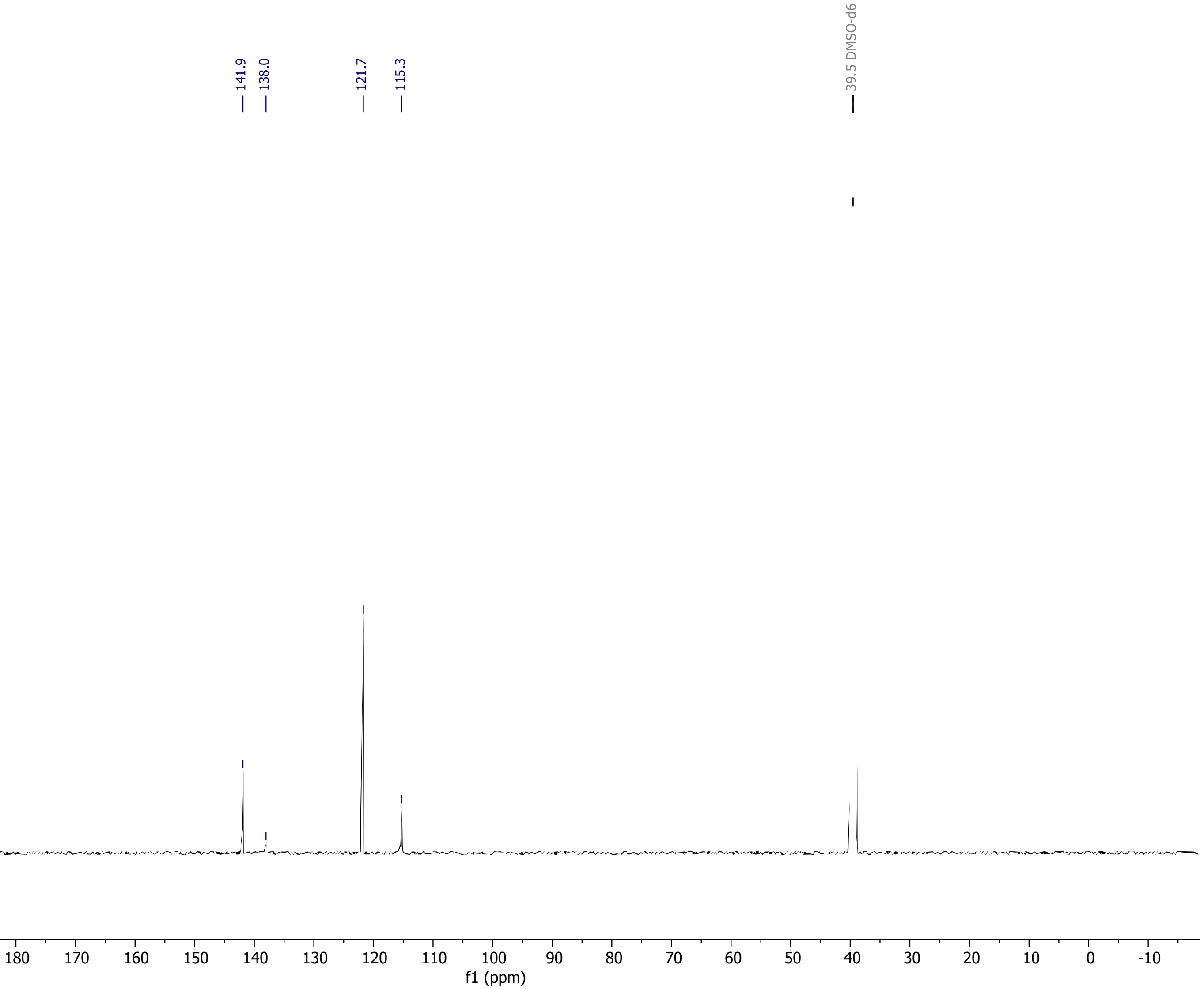

^{1}H NMR of indole (**4**) – $CDCl_3$, 23 °C

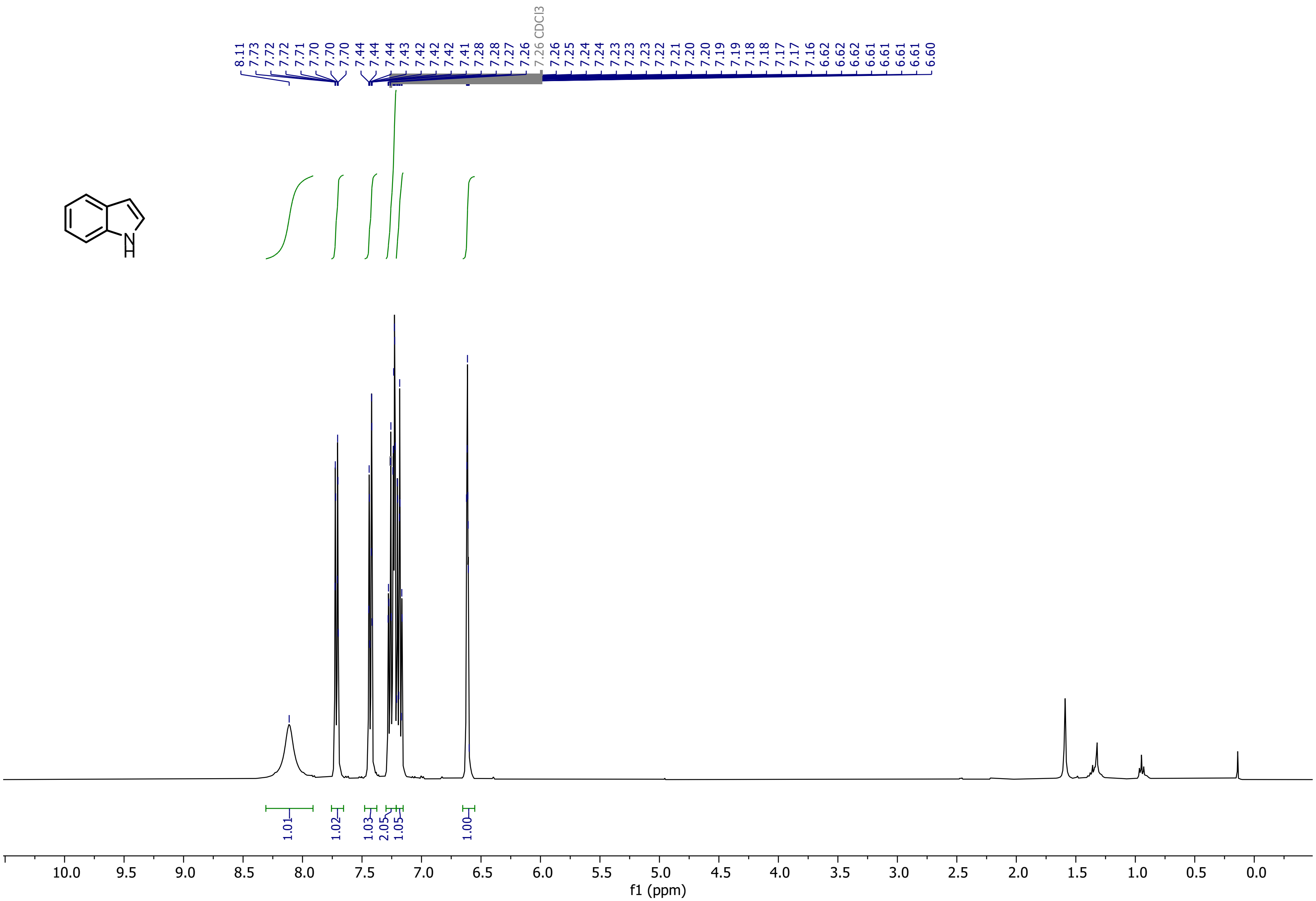

^{13}C NMR of indole (**4**) – DMSO-d6, 23 ºC

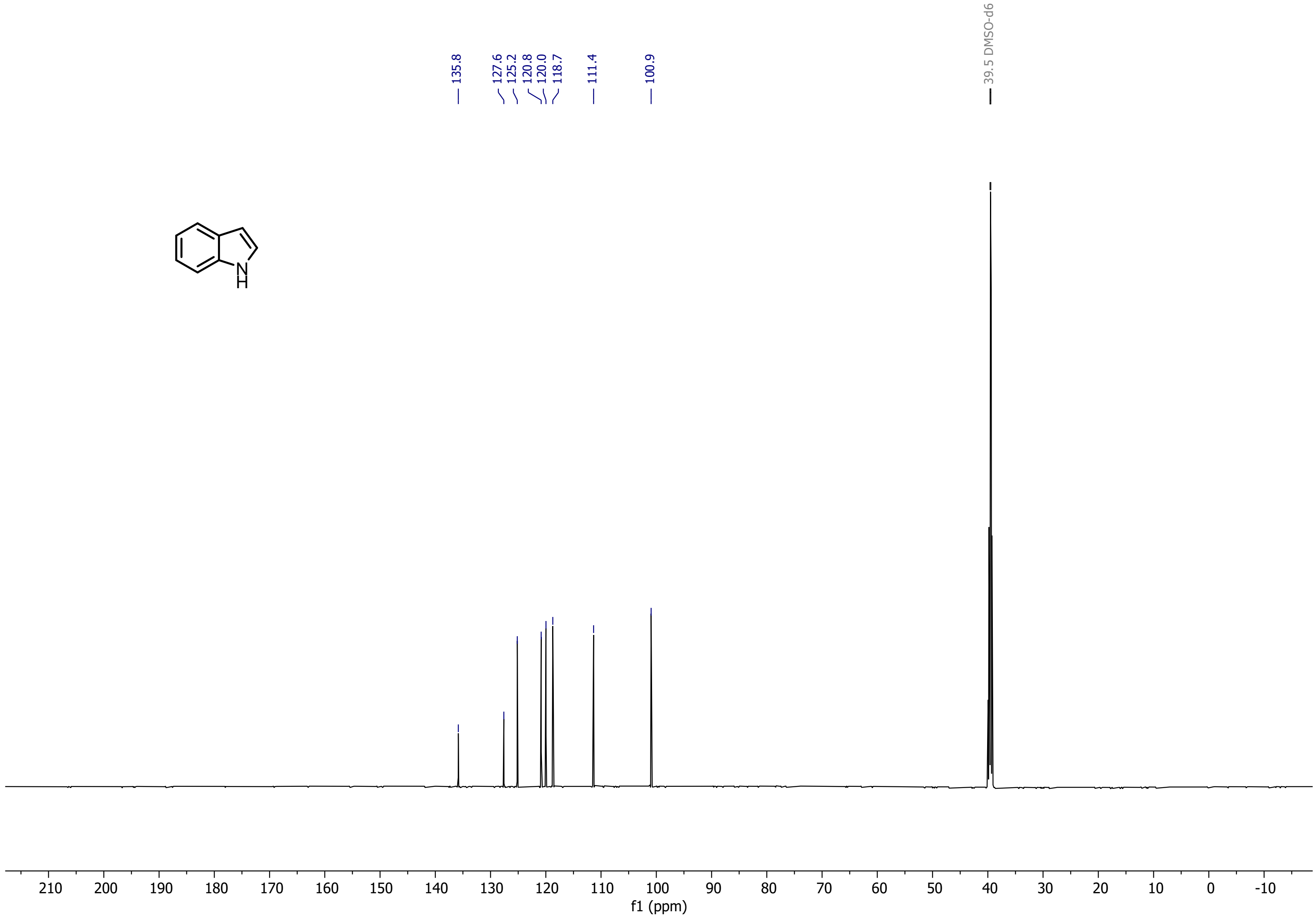

*^{1}H NMR of 1*H*-indole-3-carbonitrile (**5**) – DMSO-d6, 23 ºC*

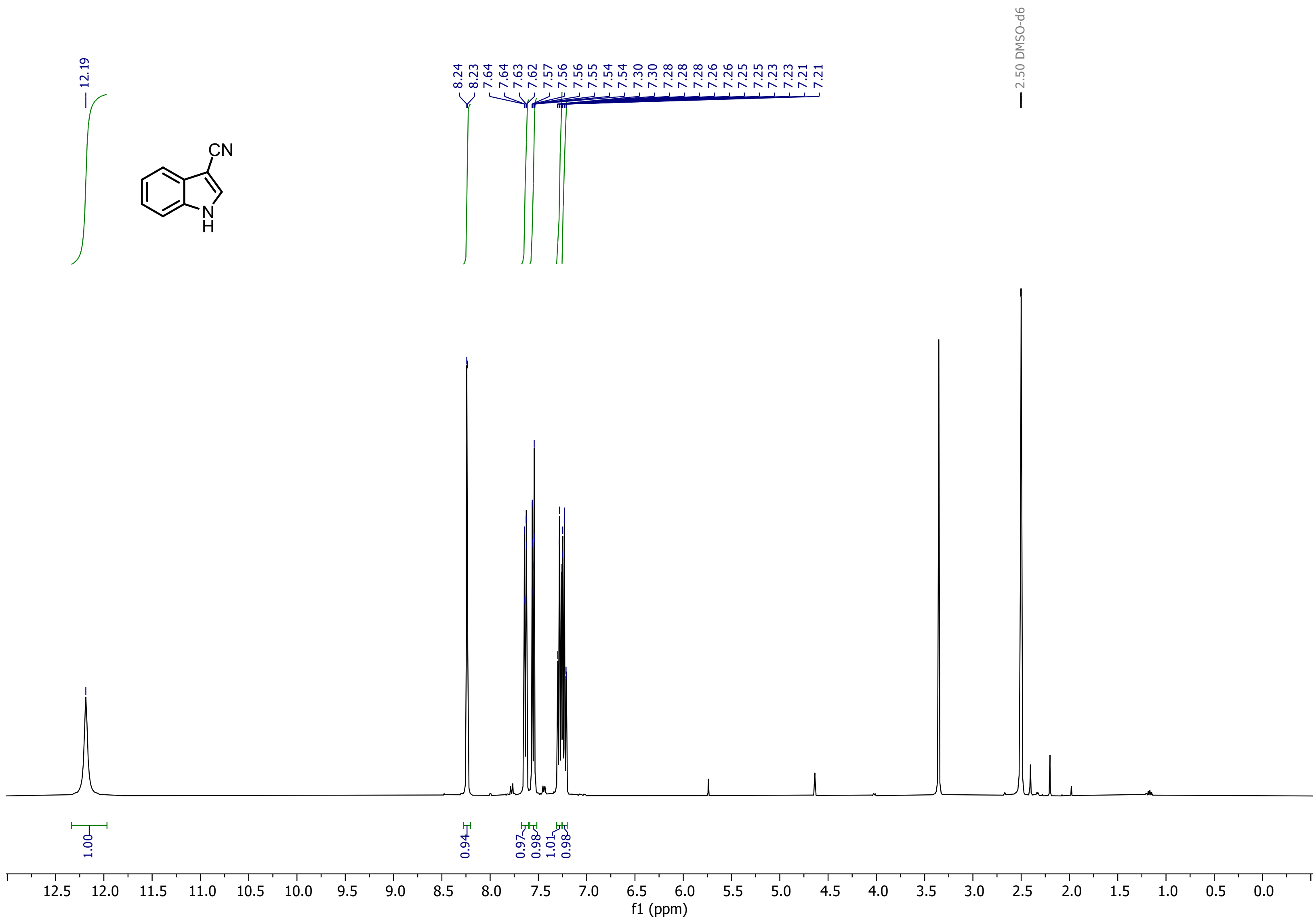

*^{13}C NMR of 1*H*-indole-3-carbonitrile (**5**) – DMSO-d6, 23 ºC*

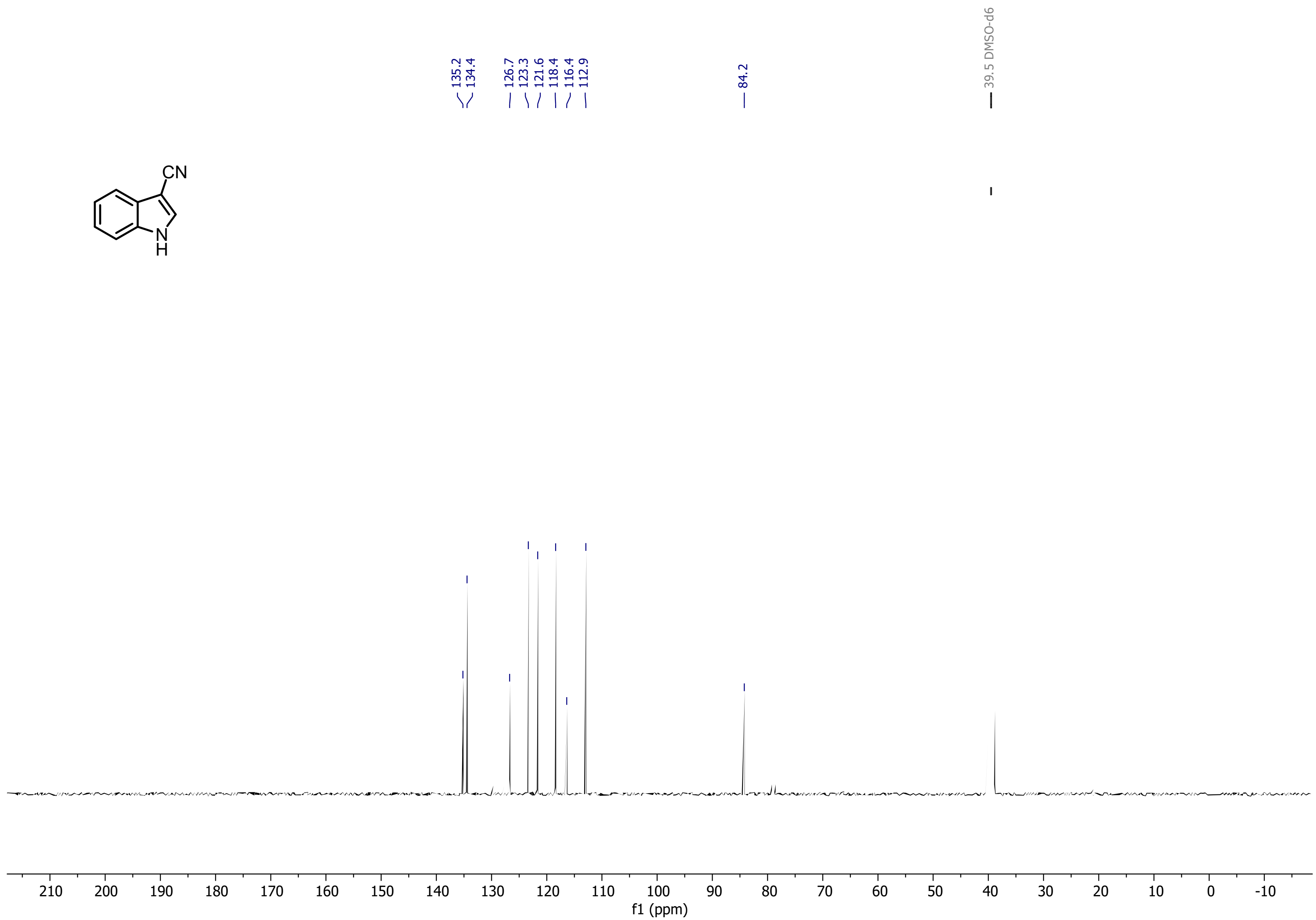

^{1}H NMR of indazole (**6**) – $CDCl_3$, 23 ºC

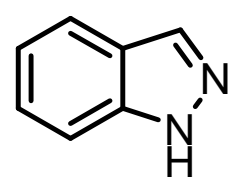

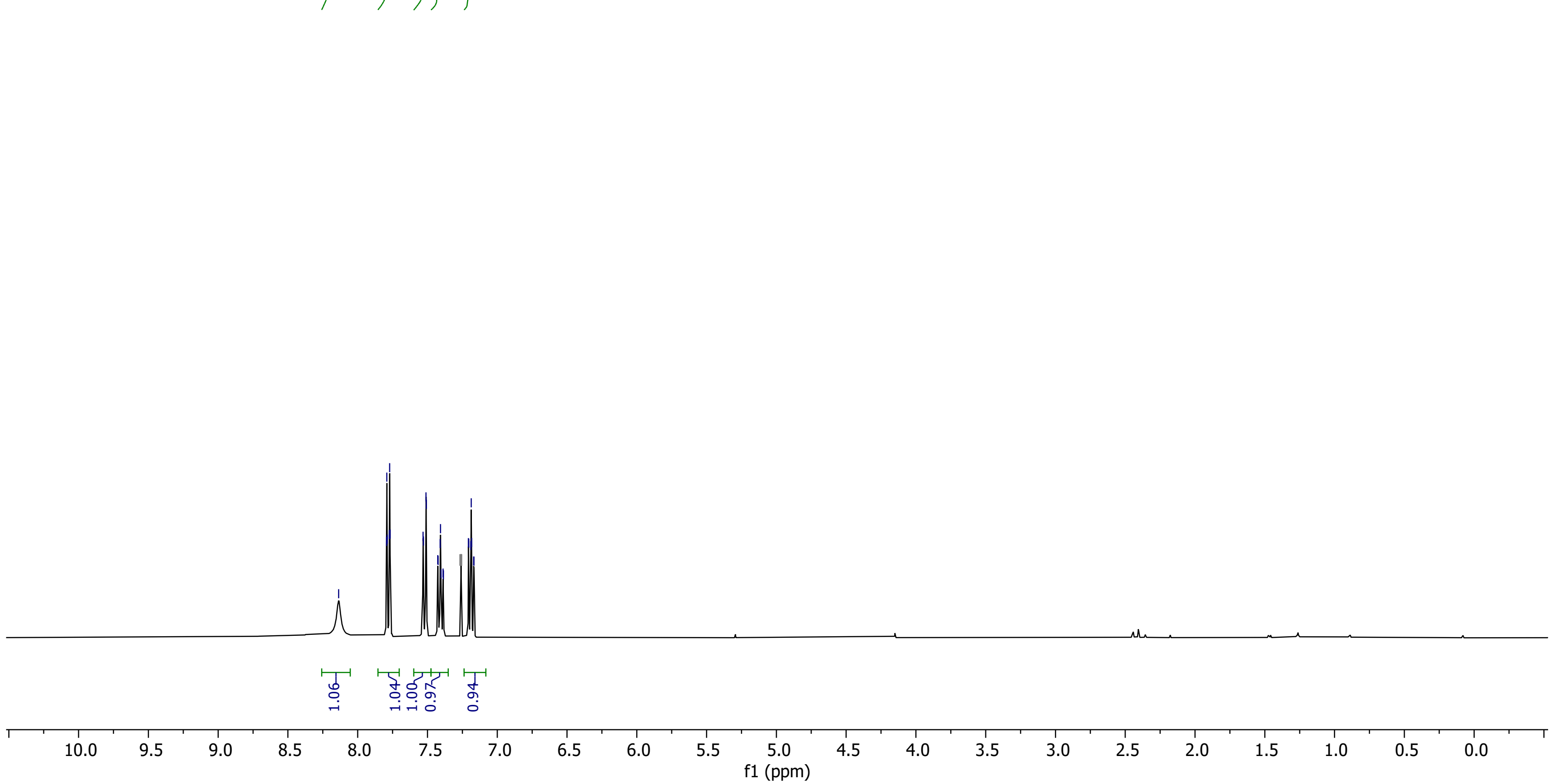

*^{13}C NMR of indazole (**6**) – $CDCl_3$, 23 ºC*

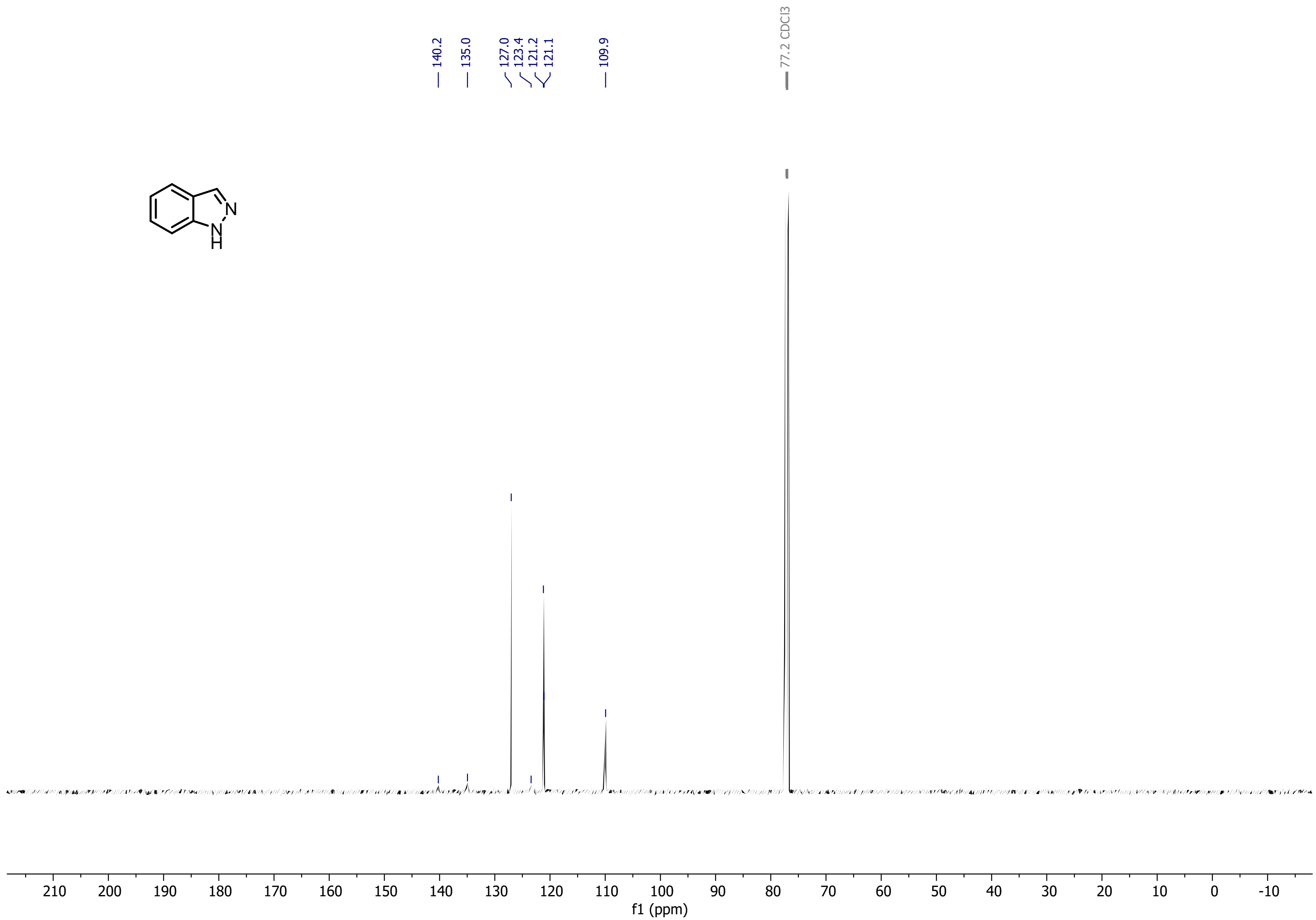

^{1}H NMR of carbazole (**7**) – DMSO-d6, 23 ºC

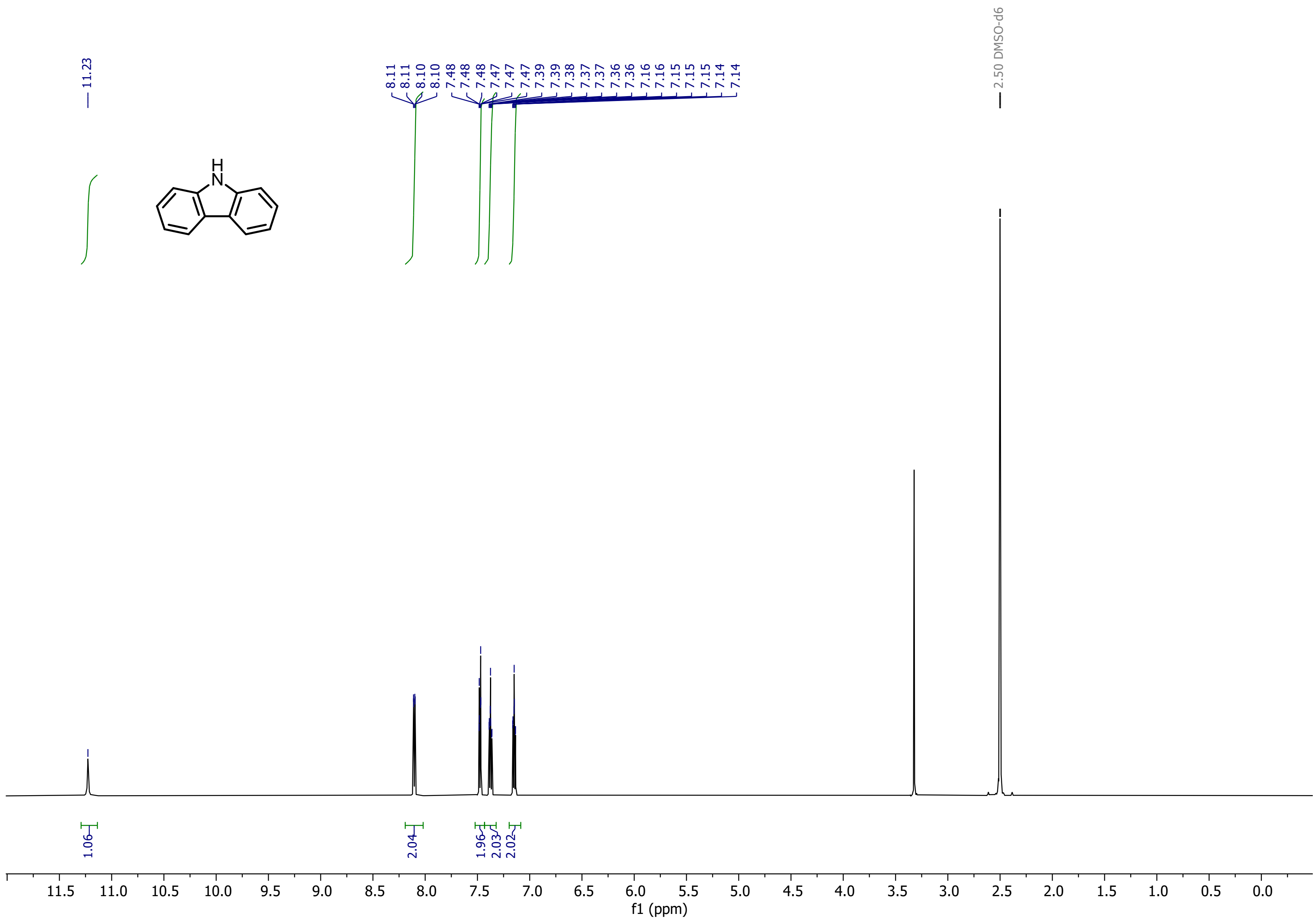

^{13}C NMR of carbazole (**7**) – DMSO-d6, 23 ºC

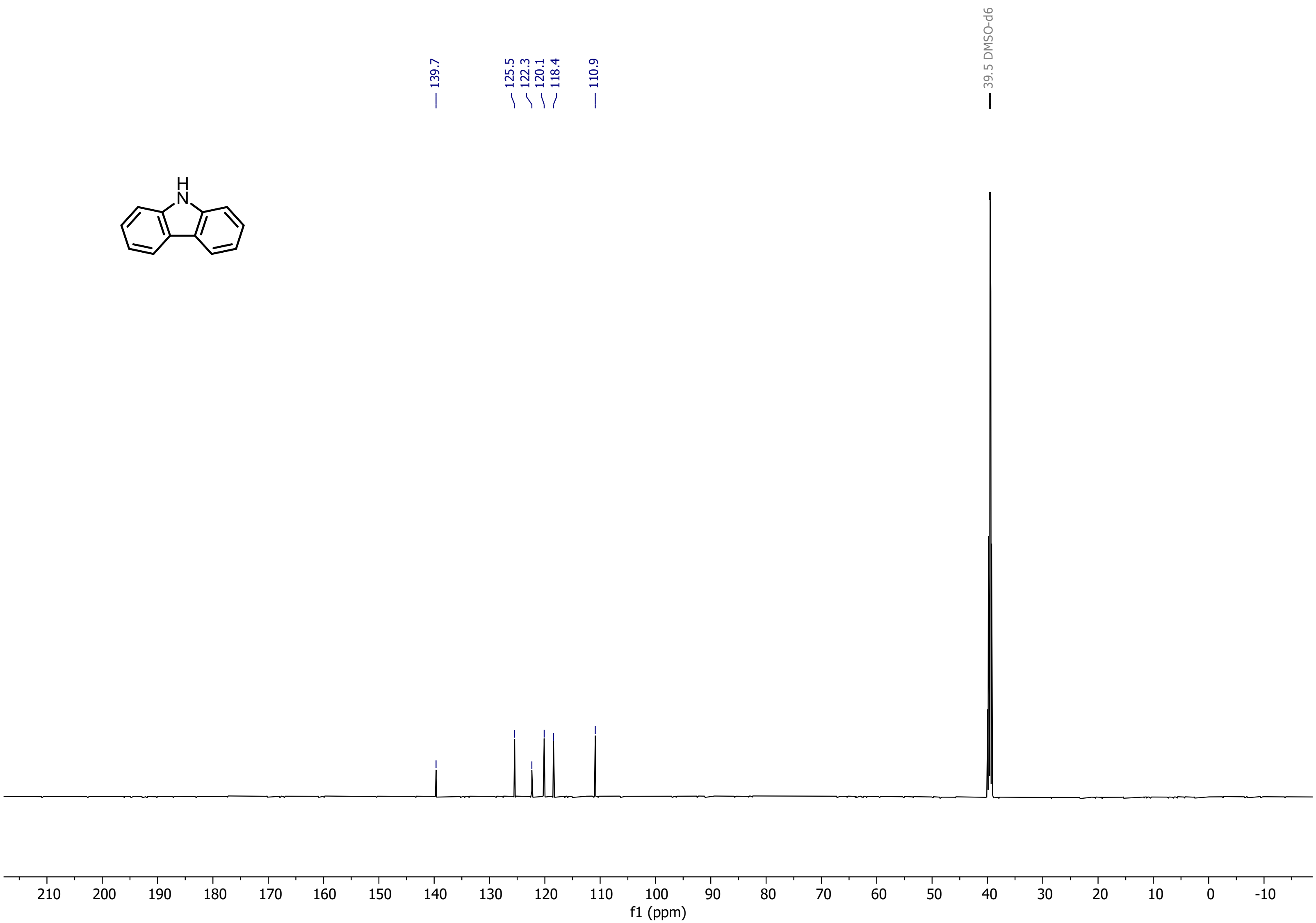

References

(1) Harris, R. K.; Becker, E. D.; De Menezes, S. M. C.; Granger, P.; Hoffman, R. E.; Zilm, K. W. Further Conventions for NMR Shielding and Chemical Shifts (IUPAC Recommendations 2008): International Union of Pure and Applied Chemistry Physical and Biophysical Chemistry Division. *Magnetic Reson in Chemistry* **2008**, *46* (6), 582–598. https://doi.org/10.1002/mrc.2225

(2) Pavlishchuk, V. V.; Addison, A. W. Conversion Constants for Redox Potentials Measured versus Different Reference Electrodes in Acetonitrile Solutions at 25°C. *Inorganica Chimica Acta* **2000**, *298* (1), 97–102. https://doi.org/10.1016/S0020-1693(99)00407-7

(3) Stoll, S.; Schweiger, A. EasySpin, a Comprehensive Software Package for Spectral Simulation and Analysis in EPR. *Journal of Magnetic Resonance* **2006**, *178* (1), 42–55. https://doi.org/10.1016/j.jmr.2005.08.013

(4) Neese, F. Software Update: The ORCA Program System—Version 6.0. *WIREs Comput Mol Sci* **2025**, *15* (2), e70019. https://doi.org/10.1002/wcms.70019

(5) Weigend, F.; Ahlrichs, R. Balanced Basis Sets of Split Valence, Triple Zeta Valence and Quadruple Zeta Valence Quality for H to Rn: Design and Assessment of Accuracy. *Phys. Chem. Chem. Phys.* **2005**, *7* (18), 3297. https://doi.org/10.1039/b508541a

(6) Caldeweyher, E.; Mewes, J.-M.; Ehlert, S.; Grimme, S. Extension and Evaluation of the D4 London-Dispersion Model for Periodic Systems. *Phys. Chem. Chem. Phys.* **2020**, *22* (16), 8499–8512. https://doi.org/10.1039/D0CP00502A

(7) Pollak, P.; Weigend, F. Segmented Contracted Error-Consistent Basis Sets of Double- and Triple-ζ Valence Quality for One- and Two-Component Relativistic All-Electron Calculations. *J. Chem. Theory Comput.* **2017**, *13* (8), 3696–3705. https://doi.org/10.1021/acs.jctc.7b00593

(8) Kutzelnigg, W.; Liu, W. Quasirelativistic Theory Equivalent to Fully Relativistic Theory. *The Journal of Chemical Physics* **2005**, *123* (24), 241102. https://doi.org/10.1063/1.2137315

(9) *Prudent Practices in the Laboratory: Handling and Management of Chemical Hazards, Updated Version*; National Academies Press: Washington, D.C., 2011; p 12654. https://doi.org/10.17226/12654

(10) MacKenzie, I. A.; Wang, L.; Onuska, N. P. R.; Williams, O. F.; Begam, K.; Moran, A. M.; Dunietz, B. D.; Nicewicz, D. A. Discovery and Characterization of an Acridine Radical Photoreductant. *Nature* **2020**, *580* (7801), 76–80. https://doi.org/10.1038/s41586-020-2131-1

(11) Leclair, A.; Wang, Q.; Zhu, J. Two-Carbon Ring Expansion of Cyclobutanols to Cyclohexenones Enabled by Indole Radical Cation Intermediate: Development and Application to a Total Synthesis of Uleine. *ACS Catal.* **2022**, *12* (2), 1209–1215. https://doi.org/10.1021/acscatal.1c05621

(12) Hicks, J.; Juckel, M.; Paparo, A.; Dange, D.; Jones, C. Multigram Syntheses of Magnesium(I) Compounds Using Alkali Metal Halide Supported Alkali Metals as Dispersible Reducing Agents. *Organometallics* **2018**, *37* (24), 4810–4813. https://doi.org/10.1021/acs.organomet.8b00803
